# Finding minimum gene subsets with heuristic breadth-first search algorithm for robust tumor classification

**DOI:** 10.1186/1471-2105-13-178

**Published:** 2012-07-25

**Authors:** Shu-Lin Wang, Xue-Ling Li, Jianwen Fang

**Affiliations:** 1 College of Information Science and Engineering, Hunan University, Changsha, Hunan, 410082, China; 2 Intelligent Computing Laboratory, Hefei Institute of Intelligent Machines, Chinese Academy of Sciences, Hefei, Anhui, 230031, China; 3 Applied Bioinformatics Laboratory, the University of Kansas, 2034 Becker Drive, Lawrence, KS, 66047, USA

**Keywords:** Gene expression profiles, Gene selection, Tumor classification, Heuristic breadth-first search, Power-law distribution

## Abstract

**Background:**

Previous studies on tumor classification based on gene expression profiles suggest that gene selection plays a key role in improving the classification performance. Moreover, finding important tumor-related genes with the highest accuracy is a very important task because these genes might serve as tumor biomarkers, which is of great benefit to not only tumor molecular diagnosis but also drug development.

**Results:**

This paper proposes a novel gene selection method with rich biomedical meaning based on Heuristic Breadth-first Search Algorithm (HBSA) to find as many optimal gene subsets as possible. Due to the curse of dimensionality, this type of method could suffer from over-fitting and selection bias problems. To address these potential problems, a HBSA-based ensemble classifier is constructed using majority voting strategy from individual classifiers constructed by the selected gene subsets, and a novel HBSA-based gene ranking method is designed to find important tumor-related genes by measuring the significance of genes using their occurrence frequencies in the selected gene subsets. The experimental results on nine tumor datasets including three pairs of cross-platform datasets indicate that the proposed method can not only obtain better generalization performance but also find many important tumor-related genes.

**Conclusions:**

It is found that the frequencies of the selected genes follow a power-law distribution, indicating that only a few top-ranked genes can be used as potential diagnosis biomarkers. Moreover, the top-ranked genes leading to very high prediction accuracy are closely related to specific tumor subtype and even hub genes. Compared with other related methods, the proposed method can achieve higher prediction accuracy with fewer genes. Moreover, they are further justified by analyzing the top-ranked genes in the context of individual gene function, biological pathway, and protein-protein interaction network.

## Background

Tumor involves many pathways, distinct genes and exogenous factors, and is considered as systems biology diseases [1]. Despite tremendous efforts in research, the mechanism of tumor genesis and development has not been thoroughly known yet. Treatment of later stage cancers is often not therapeutically effective, and medical experts agree that early diagnosis of tumor is of great benefit to successful therapies. However, early tumor detection is extremely difficult using traditional tumor mass detection techniques such as X-ray imaging. Furthermore, different subtypes of tumor show very different responses to therapy, indicating that they are molecularly distinct entities. Thus, accurate classification of tumor samples based on molecular signatures is essential for efficient cancer treatment. Since the first paper on the classification of leukemia subtype based on Gene Expression Profiles (GEP) was published [2], this research field has been studied extensively and become a research hotspot [3-8]. Many datasets on different tumors have been published such as colon tumor [9], Small Round Blue Cell Tumor (SRBCT) [10], Diffuse Large B-Cell Lymphomas (DLBCL) [11], and prostate tumor [12], etc. All of the published tumor datasets have very high dimensionality and small sample size mainly due to limited resources and the time required for collecting and genotyping specimens [13]. Many supervised classification methods in pattern recognition, such as Support Vector Machines (SVM) [14,15], Artificial Neural Networks (ANN) [16-20], *k*-Nearest Neighbor (KNN) [12,21], and nearest shrunken centroids [22], have been successfully applied to GEP-based tumor classification over the last decade. All these studies have shown that GEP-based tumor classification methods hold great promises for early diagnosis and clinical prognosis of tumor. However, due to the challenges from the curse of dimensionality that the number of genes far exceeds the size of sample set, dimensionality reduction including feature extraction such as total principal component regression [23] and gene selection [2] should be performed before constructing classification model [24]. Compared to feature extraction, gene selection do not alter the original representation of genes, so it can not only improve the performance of tumor classification by removing redundant and irrelevant genes but also select informative gene subsets that may serve as cancer biomarkers and potential drug targets. More importantly, it may provide insight into the underlying molecular mechanism of tumor development. Therefore gene selection plays a very important role in tumor classification [25].

Generally, gene selection can be classified into two categories: Filters and Wrappers [26]. Filters are independent from the following classification stage. They evaluate the discriminability of genes by using only the intrinsic information of data themselves and subclass information, such as relative entropy [27], information gain and *t*-test [28], as well as Minimum Redundancy-Maximum Relevance (mRMR) [29]. Because gene selection is not associated to any specific classifiers, the gene subsets selected by Filters can avoid over-fitting phenomena. The advantage of Filters is that they can be easily catered to very high-dimensional datasets, and are computationally simple and fast [25]. On the contrary, Wrappers evaluate the discriminability of each gene subset using the evaluation function of learning algorithm, such as Genetic Algorithm (GA)/SVM method [30] and GA/KNN method [21]. Wrappers often deliver better performance than Filters in gene selection [26] because they utilize the feedback information of classification accuracy. However, their computational cost must be seriously taken into account [31] due to the fact that hunting for the smallest feature sets in a high-dimensional space is an NP-complete problem [32,33]. Practically for all Wrappers a good solution is to adopt heuristic method in a condensed search space to approximately find out the smallest feature sets. One example is to adopt GA to find the most informative gene subsets [21,34]. Another example is to combine gene ranking with clustering analysis to select a small set of informative genes [35].

Three general modes are commonly adopted in gene selection strategies: Increasing Mode, Decreasing Mode, and Hybrid Mode, which are respectively introduced as follows. 1) Increasing Mode selects a gene subset starting from empty set until a gene subset with the highest classification accuracy is selected through appending potential genes into the gene subset, such as Sequential Forward Search (SFS) [36]. 2) Decreasing Mode starts from the whole gene set to remove irrelevant and redundant genes, and keeps the least gene subset among the subsets with the same classification accuracy, such as the well-known Support Vector Machine-Recursive Feature Elimination (SVM-RFE) [14] that selects informative genes in a sequential backward elimination manner by starting with the whole gene set and eliminating one or several redundant gene in each iteration, and the extension of SVM-RFE(MSVM-RFE) [37] that solves the multi-class gene selection problem by simultaneously considering all subclasses during the gene selection process. 3) Hybrid Mode, such as Sequential Forward Floating Search (SFFS) algorithm [36] and Markov blanket-embedded genetic based gene selection algorithm [34], combines Increasing Mode with Decreasing Mode by starting from an arbitrary gene set. However, Reunanen [38] proved that intensive search strategies such as SFFS do not necessarily outperform a simpler and faster method like SFS, provided that the comparison is done properly.

In fact, due to the characteristics of GEP, more complex methods are not obviously superior to the simpler ones and the loss of biomedical meaning derived from the over-complex methods may be not sufficiently compensated by the little improvement of predictive performance [39]. Therefore, designing biologically interpretable methods that obtains minimum gene subsets with the highest or nearly highest classification accuracy is very important for robust tumor classification. Furthermore, identifying minimum gene subsets means discarding most noise and redundancy in dataset to the utmost extent, which may not only improve classification accuracy but also decrease the tumor diagnosis cost by suggesting the fewest biomarkers in clinical application as suggested by [35,40,41]. However, the curse of dimensionality from GEP implicates two problems in selecting a small gene subset with the highest or nearly highest accuracy from thousands of genes: over-fitting and selection bias, because it may be just by chance to find a small gene subset with perfect classification performance from such tremendous gene space even in random dataset [42-44]. So the over-fitting and selection bias problems must be avoided in order to obtain robust classification performance.

Ambroise *et al.*[45] found that overoptimistic results incurred by selection bias could happen if test set is not thoroughly excluded from gene selection process. Therefore, test set must be independent of the training process of classifier. Wang L.P. *et al.*[46] further pointed out that many previous studies, such as [47] and [10], had gained overoptimistic performance according to this criterion, and they proposed a simple method with resultant accurate tumor classification by using a very few genes. This method combines gene ranking with exhaustive search method to find minimum gene subsets so as to achieve the unbiased accuracy. Although their methods achieve good and unbiased classification results, the high computational cost makes it infeasible when the number of initially selected genes is very large (e.g., more than 300). Our previous work [48] designed a gene selection approach that was used to find the minimum gene subsets with the highest classification accuracy, but seriously upward bias occurred because that initially selecting differentially expressed genes on whole dataset and over-fitting is performed in gene selection stage. In this study, based on the Heuristic Breadth-first Search Algorithm (HBSA), we further construct a HBSA-based ensemble classifier and design a HBSA-based gene ranking method by counting its occurrence frequency on the basis of gene subsets selected only on training set so as to avoid over-fitting case and selection bias. Our novel method manages to simultaneously achieve the two conflict goals [49]: 1) Design a simple classifier to achieve nearly highest and unbiased prediction accuracy; and 2) Mine as many important tumor-related genes as possible, which may provide insight into the mechanism of tumor genesis and help find diagnosis biomarkers and new therapeutic targets [50].

In this following section, we firstly describe the classification problem and introduce the search strategy of HBSA. The implementation of HBSA is given, and its biomedical interpreter is also illustrated. Then two methods including HBSA-based ensemble classification and HBSA-based gene ranking are designed to obtain unbiased prediction accuracy and find important tumor-related genes. The results obtained on nine actual tumor datasets including three pairs of cross-platform datasets demonstrate the feasibility and effectiveness of our method. Comparison with other related methods also indicate the superiority of our method. The biomedical analysis of the selected genes in the context of individual gene function, pathway analysis and Protein-Protein Interaction (PPI) network further justify our methods.

## Methods

### Problem description

Let *G* = {*g*_1_, ⋯, *g*_*n*_} be a set of genes and *S* = {*s*_1_, ⋯, *s*_*m*_} be a set of samples. |*G*| = *n* denotes the number of genes, and |*S*| = *m* denotes the number of samples. The corresponding GEP can be represented as matrix *X* = (*x*_*i*,*j*_)_*mn*_, 1 ≤ *i* ≤ *m*, 1 ≤ *j* ≤ *n*, where *x*_*i*,*j*_ is the expression level of gene *g*_*j*_ in sample *s*_*i*_, and usually *n* ≫ *m*. Each vector *s*_*i*_ in the gene expression matrix can be regarded as a point in *n*-dimensional space. And each of the *m* rows consists of an *n*-element expression vector for a single sample. Let *L* = {*c*_1_, ⋯, *c*_*k*_} denote the label set and |*L*| = *k* denote the number of subclasses. Usually, the subclass of each sample is known, so *S* × *L* = {(*s*_*i*_, *l*_*i*_)|*s*_*i*_ ∈ *R*^*n*^, *l*_*i*_ ∈ *R*^*n*^, *l*_*i*_ ∈ *L*, *i* = 1, 2, ⋯, *m*} denotes the labeled sample space.

Selecting an informative gene subset *T* with the highest classification accuracy from gene space *P*(*G*) (the power set of *G*) is a crucial problem, but it is an NP-complete problem [33]. Moreover, which and how many genes are relevant to a specific tumor subtype are not clear for biomedical scientists so far. We therefore assume that the gene subsets with powerful classification ability are relevant to a specific tumor subtype. Let *Acc*(*T*) denote the classification ability of a gene subset *T* on sample set, which is usually measured by the accuracy of a classifier. We hope that the selected informative gene subset *T* simultaneously satisfies the following two goals:

(1) $$minimize_{T\in P(G)}(|T|)$$

(2) $$miximize_{T\in P(G)}(Acc(T))$$

where |*T*| denotes the cardinal number of gene subset *T*. The gene subset simultaneously satisfying (1) and (2) is called an optimal gene subset T^*^. Note that usually more than one optimal gene subset T^*^ may exist in that the genes belonging to the same pathway in a cell usually have similar expression pattern and function. Optimal subsets A^*^ comprise all of the optimal gene subsets T^*^, i.e., A^*^ = {T^*^|T^*^ ⊂ GT^*^ simultaneously satisfies (1) and (2)}. Although finding only one gene subset T^*^ is sufficient for tumor classification, finding as many optimal gene subsets as possible is very useful to gain an insight into tumor dataset structure and discover more important tumor-related genes.

Due to a large |*G*| = *n* (e.g., one sample usually includes 2 000 ~ 30 000 genes), it is impractical to apply an exhaustive search method to find out A^*^ in the space of 2^*n*^ gene subsets. A good solution is to adopt a heuristic method in a condensed search space to approximately find out A^*^. However, different gene subsets with different cardinal numbers may be selected by using different methods, so it is difficult to determine the minimum number of optimal gene subset for a specific tumor dataset by only designing methods. Thus we must balance the minimum number and the classification accuracy. Jain *et al.*[51] suggested a criterion (3) that the number of training samples per subclass is at least five times the number of features in designing a classifier to avoid the curse of dimensionality, i.e.

(3) $$(m_{t}/k)/n_{s}>5$$

where *k* is the number of subclasses, *m*_*t*_ is the number of training samples, and *n*_*s*_ denotes the number of the selected genes, and for more complex classifier the ratio of sample size to dimensionality should be larger. For example, we should consider at most eight informative genes for two-subclass tumor dataset with only 80 training samples to design a classifier with acceptable generalization performance [52]. Considering that very high accuracy can always be obtained by selecting sufficient genes in a small size of sample set, we aim to find minimal gene subsets with nearly maximal accuracy rather than to obtain maximal accuracy with much more genes. Therefore, those gene subsets approximately satisfying (1) and (2) are also included into our optimal gene subsets A^*^. Based on these optimal gene subsets, how to obtain more reliable accuracy and find more important tumor-related genes are two key problems.

### Gene pre-selection

It is widely accepted that tumor-related genes are differentially expressed ones, so the Filters-based gene ranking techniques are usually used to pre-select the differentially expressed genes from the original gene space even though those differentially expressed genes are not always tumor-related ones due to the noises in dataset. Its main idea is to assign each gene a single score that denotes the significance of each gene according to a certain scoring criterion. Many single variable methods such as *t*-test and Bhattacharyya distance are extensively used as discrimination criterions. However, these methods require the dataset to follow Gaussian distribution. Otherwise, these methods may not achieve optimal experimental performance. Deng *et al.*[53] reported that usually tumor datasets do not follow Gaussian distribution and showed that Wilcoxon rank sum test (WRST) is superior to *t*-test method in gene selection on three binary tumor datasets. However, WRST is only suitable for the binary classification problem. Kruskal-Wallis rank sum test (KWRST) is suitable for multi-class problem. The WRST or KWRST-based gene selection method was reported to perform very well in GEP-based tumor classification on the basis of the extensive comparison studies [24,54]. Taking it into consideration that KWRST does not require a certain distribution of data and is also suitable for small dataset, in our experiments we use KWRST to pre-select an initial informative gene set G^*^ = {*g*_1_, ⋯*g*_*p*_}, which contains *p* candidate genes with good discriminating ability.

### Heuristic breadth-first search

#### Search strategy

We aim at finding as many optimal gene subsets as possible. When *p*, the number of the informative genes pre-selected by KWRST, is small, breadth-first search algorithm can realize our goals (1) and (2). However, when *p* is very big (e.g. *p* = 300), the required CPU time of such a search algorithm is intolerable. We therefore design a heuristic breadth-first search algorithm (HBSA) with heuristic information measured by *Acc*(*T*) to find the optimal gene subsets A^*^, which can drastically reduce the search space.

**Figure 1:**
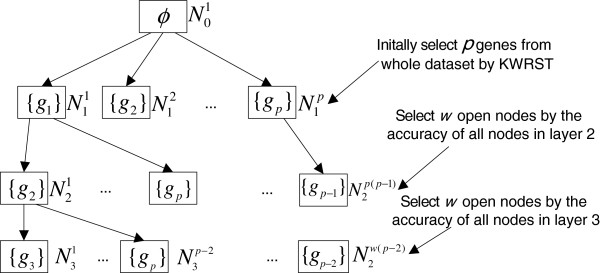
A diagram of the expanded tree generated by HBSA.

Usually, in the process of search an expanded tree is generated by HBSA from G^*^ = {*g*_1_,.,*g*_*p*_}, which is the differentially expressed gene set pre-selected by KWRST, as shown in Figure 1, where *N*_i_^j^ denotes a node with *i* representing the layer of the node (0 ≤ *i* ≤ *p*) and *j* the serial number of the node in layer *i*. The data structure of each node is defined as follows:

Node = Begin

* set*;

* parent*;

* path*;

* c*;

End

where *N*_i_^j^.*set* denotes a set only containing single gene, *N*_i_^j^.*parent* the parent node of the node *N*_i_^j^, and *N*_i_^j^.*path* a gene set containing all genes on the path from the root node *N*_0_^1^ to the node *N*_i_^j^ itself. Obviously the length of the gene set *N*_i_^j^.*path* is *i*, i.e., *N*_i_^j^.*path* = *N*_i_^j^.*parent*.*path* ∪ *N*_i_^j^.*set*. Let *N*_i_^j^.*c* = Acc(*N*_i_^j^.*path*) denote the classification accuracy of the gene subset *N*_i_^j^.*path*, serving as the heuristic information to guide the node selection in layer *i*, evaluated by SVM and KNN classifiers here. For the root node *N*_0_^1^, *N*_0_^1^.*set* = ∅ (∅ is empty set), *N*_0_^1^.*path* = ∅, *N*_0_^1^.*parent* = *nil*, and *N*_0_^1^.*c* = 0. The root node is expanded to *p* child nodes guided by the heuristic information *KWRST*(*g*), where we set *N*_i_^j^.*set* = {*g*_*j*_} and *N*_0_^1^.*path* = {*g*_*j*_}, *g*_*j*_ ∈ G^*^, 1 ≤ *j* ≤ *p*. Next all *p* nodes are expanded again in next layer. Each node *N*_i_^j^(1 ≤ *j* ≤ *p*) in layer 1 is expanded to *p* − 1 child nodes, thus there are *p*(*p* − 1) nodes in layer 2, where *N*_2_^*j*^.*set* = {*g*_*i*_}, *N*_2_^*j*^.*path* = *N*_2_^*j*^.*set* ∪ *N*_2_^*j*^.*parent*.*path*, *N*_2_^*j*^.*c* = *Acc*(*N*_2_^*j*^.*path*), *g*_*i*_ ∈ G^*^ ∧ *g*_*i*_*∉ N*_2_^*j*^.*parent*.*path*, where 1 ≤ *j* ≤ *p*(*p* − 1), 0 ≤ *i* ≤ *p*. Then we descendingly rank all nodes in layer 2 by their *N*_2_^*j*^.*c*, and examine whether *Acc*_*max*_(2) = max_1 ≤ *j* ≤ *p*(*p*−1)_(*N*_2_^*j*^.*c*) is greater than a given threshold *Acc_Max* or not, where *Acc*_*max*_(2) denotes the maximal accuracy in layer 2.If *Acc*_*max*_(2) ≥ *Acc_Max*, where *Acc_Max* is a given threshold, which indicates that at least one optimal gene subset is found, the searching process is stopped. Otherwise in layer 2 we select the *w* top-ranked nodes as open nodes to be expanded in next layer, where the parameter *w* denotes the search breadth. In fact, whatever *w* is set to, *w* always takes *p* value in layer 1. The rest may be deduced by analogy. Note that some gene subsets on different paths are possibly the same regardless of gene order in these gene subsets. Except nodes in layer 0 and 1, if the classification accuracy of a node has been previously computed, the accuracy of this node is set to zero so as to avoid the unnecessary expansion and this node is called closed node that will not be expanded in next layer. Finally, when the search process is stopped, all gene subsets in the *w* top-ranked nodes in last layer are selected into the optimal gene subsets A^*^. An example of HBSA is illustrated in Additional file 1: Figure S1.

The goal of HBSA is to select as many optimal gene subsets as possible only on training set. For each gene subset in A^*^ constructed from empty set, its classification accuracy monotonously increases with the increase of its size, so when the classification accuracy of the gene subset achieves *Acc_Max* threshold or the maximal value (100%), the size of the gene subset obtained is minimal. It, therefore, is apparent that the optimal gene subsets in A^*^ just approximately satisfy the two goals in (1) and (2). If the search breadth *w* is set appropriately, the error prone of searching process can be avoided to some extent so that as many optimal gene subsets as possible can be selected.

Obviously, the search breadth of increase with the increase HBSA does not exponentially of search depth. Thus our HBSA is a beam search algorithm, or an optimization of best-first search that searches a graph by ordering all partial solutions according to some heuristic information. As a result, only the best partial solutions of the predetermined number are kept as candidates. That is, only the most promising nodes are retained for further expanding at each layer of the search tree, while the remaining nodes are pruned off permanently [55]. Generally speaking, in local view the HBSA-based gene selection belongs to the Increasing mode, while in global view such gene selection belongs to Hybrid mode in that most of the gene combinations with lower classification accuracy are discarded in the search process. The HBSA can be implemented more flexibly. For example, it is unnecessary to select fixed *w* top-ranked nodes to be expanded in each layer, that is, *w* can be set to different values in different layers. There are two modes to set *w*. 1) For each layer, *w* can be determined by the distribution of the classification accuracy of all nodes in the corresponding layer. 2) Set different *Acc_Max* thresholds for different layers, and the given threshold of each layer must be less than that of its next layer, which leads to different numbers of the selected nodes in different layers. Thus, one advantage of HBSA is its adaptability.

Another advantage of HBSA is its biomedical interpretation. Suppose *T*_*i*_ = {*g*_1_^′^, ⋯, *g*_*i*_^′^} is a selected gene subset with high accuracy in the *i*-th layer, where *g*_*j*_^′^ ∈ G^*^, 1 ≤ *j* ≤ *i*. If *g*’_*i*+1_ ∈ G^*^ could be appended into *T*_*i*_ to make *Acc* (*T*_*i*+1_ = {*g*’_1_, ⋯, *g*’_*i*_, *g*’_*i*+1_}) increase maximally, *g*’_*i*+1_ should be independent of or very weakly related to the genes in gene subset *T*_*i*_ ideally. Otherwise, if *Acc* (*T*_*i*+1_) increases only a little or even decreases, the subset *T*_*i*+1_ will be discarded in layer *i* + 1. Therefore, ideally, all genes in the optimal set T^*^ should be independent of each other, and each optimal gene subset T^*^ selected should be an independent variable group. It implies that those genes in subset T^*^ should be on the different regulatory pathways, but, due to much noise in GEP, the gene latterly appended into the gene subset might be weakly tumor-related.

**Figure 2:**
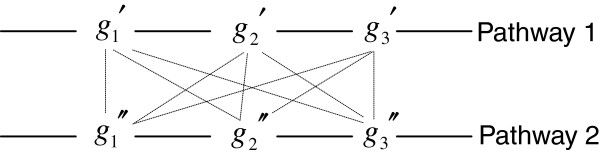
**A diagram of two regulatory pathways.** The dotted lines represent all possible combinations of genes on different pathways.

Moreover, to distinguish which genes are more important ones, the significance of a gene is measured by its occurrence frequency counted in the optimal set A^*^. The bigger the occurrence frequency of a gene, the more important the gene. This definition also has its biomedical interpretation. For example, given two three-gene subsets *G*_1_ = {*g*_1_^′^, *g*_2_^′^, *g*_*3*_^′^} and *G*_2_ = {*g*_1_^″^, *g*_2_^″^, *g*_*3*_^″^}, where we assume that all genes in *G*_1_ are on pathway 1 and all genes in *G*_2_ are on pathway 2, as shown in Figure 2, and that the tumor-related strengths of the genes in *G*_1_ and *G*_2_ decrease with their orders in *G*_1_ and *G*_2_, respectively. Generally, gene subsets such as {*g*_1_^′^, *g*_2_^′^} and {*g*_1_^″^, *g*_2_^″^} might not be selected by HBSA because both *Acc*({*g*_1_^′^, *g*_2_^′^}) and *Acc*({*g*_1_^″^, *g*_2_^″^}) might be lower than that of other irrelevant gene combinations such as {*g*_1_^′^, *g*_2_^″^} due to the expression similarity of genes on the same pathways. Thus the potential gene combinations include nine gene subsets possibly selected: {*g*_1_^′^, *g*_1_^″^}, {*g*_1_^′^, *g*_2_^″^}, {*g*_1_^′^, *g*_3_^″^}, {*g*_2_^′^, *g*_1_^″^}, {*g*_2_^′^, *g*_2_^″^}, {*g*_2_^′^, *g*_3_^″^}, {*g*_3_^′^, *g*_1_^″^}, {*g*_3_^′^, *g*_2_^″^} and {*g*_3_^′^, *g*_3_^″^}. Particularly, such gene subsets including *g*_1_^′^ and *g*_1_^″^ tend to be selected by HBSA, while those gene subsets including *g*_3_^′^ and *g*_3_^″^ incline to be discarded by HBSA, which results in high occurrence frequency of those important tumor-related genes such as *g*_1_^′^ and *g*_1_^″^ in gene set A^*^. Thus, the resultant occurrence frequency of a gene is a reasonable measure of its importance from this point of view.

#### Implementation

In practice, there is no need to construct searching tree to obtain the optimal gene subsets A^*^. It is enough to preserve the potential gene subsets and their classification accuracy in the searching process. To conveniently implement HBSA, a classification matrix *CM* = (*a*_*i,j*_)_w×p_ is defined as follows:

(4) $$CM=\left[\begin{array}{cccc} \; & \left\{g_{1}\right\} & \cdots & \left\{g_{p}\right\}\\ T^{1} & a_{1,1} & \cdots & a_{1,p}\\ \vdots & \vdots & \ddots & \vdots \\ T^{w} & a_{w,1} & \cdots & a_{w,p} \end{array}\right]$$

Adopting row label vector *Row* = (*T*^1^, *T*^2^, ⋯*T*^*W*^) to label every row of *CM* in turn, where *T*^*i*^(1 ≤ *i* ≤ *w*) denotes the selected gene subsets. Adopting column label vector *Column* = ({*g*_1_}, ⋯ {*g*_*j*_}, ⋯ {*g*_*p*_}) to label each column of *CM* in turn, where *g*_*j*_ ∈ G^*^,and *a*_*i,j*_ = *Acc*(*Row*[*i*] ∪ *Column*[*j*]), where *Row*[*i*] denote the gene subset of the *i*-th row in *CM* matrix and *Column*[*j*] denote the single gene set of the *j*-th column in *CM* matrix, 1 ≤ *i* ≤ *w,* 1 ≤ *j* ≤ *p*. The framework of HBSA is shown in Algorithm 1, where *Acc*(*T*) is defined as the classification accuracy of gene subset *T*. For example, if *Row*[5] = {*g*_1_,*g*_4_} and *Column*[3] = {*g*_6_}, *a*_5,3_ = *Acc*(*Row*[5] ∪ *Column*[3]) = *Acc*({*g*_1_,*g*_4_,*g*_6_}), which is the prediction accuracy of the gene subset {*g*_1_,*g*_4_,*g*_6_}.

**Algorithm 1**: HBSA(*M*, *p*, *w*, *Acc_Max*, *Depth*)

**Input:***M* denotes gene expression profiles, *p* the number of pre-selected genes, *w* the number of the selected gene subsets in each layer (searching breadth), *Acc_Max* a given maximal accuracy threshold, and *Depth* the upper bound of searching depth.

**Output:** A set of optimal gene subsets A^*^.

1: **For** each gene *g*_*j*_ ∈ *G* do

2: *B*[*j*]: = *KWRST*(*g*_*j*_); //Compute *p*-value for each gene by Kruskal-Wallisrank sum test.

3: **End For**

4: *B*: = *Sort*(*B*); //Rank *B* by ascending order.

5: G^*^: = *Selected*(*G*, *B*, *p*); //Select the *p* top-ranked genes as initial informative gene set G^*^from original gene set *G* according to *B*.

6: **For** each i ∈ {1,2,⋯, *p*}

7: *Column*[*i*]: = {*g*_*i*_};

8: *Row*[*i*]: = *Column*[*i*];

9: **End For**

10: *iter*: = 1; //The times of iteration.

11: **Repeat** //If *CM* is firstly computed, *CM* is a symmetric matrix, so only the lower triangle matrix of *CM* is computed.

12: Construct the classification matrix *CM*, label each row of *CM* with each component of *Row* vector, and label each column of *CM* with corresponding component *Column* vector.

13: Compute classification matrix *CM*, where *a*_*i,j*_ = *Acc*(*Row*[*i*] ∪ *Column*[*j*]), 1 ≤ *i* ≤ *w,* 1 ≤ *j* ≤ *p*; //Before *a*_*i,j*_ is computed, the sample set labeled with *Row*[*i*] ∪ *Column*[*j*] must be normalized (where the sample mean is zero while the variance is 1); function *Acc*(.) is measured by SVM with Gaussian radial basis function (RBF) kernel or KNN classifier. Computing matrix *CM* is equivalent to doing the classification accuracy of all nodes in a layer shown in Figure 1.

14: Convert *CM* to the vector *V*: = (*v*_1_, *v*_2_, ⋯, *v*_*w*×*p*_), and set *V*[(*i* − 1) × *p* + *j*].*subset*: = *Row*[*i*] ∪ *Column*[*j*], and *V*[(*i* − 1) × *p* + *j*].c: = *a*_*i*,*j*_, 1 ≤ *i* ≤ *w,* 1 ≤ *j* ≤ *p*, then rank vector *V* by *V*.*c* in descending order. Select *w* top-ranked components to reconstruct label vector *Row*[*i*]: = *V*[*i*].subset, 1 ≤ *i* ≤ *w*, where the row dimensionality of matrix *CM* can be dynamically changed according to the requirement.

15: *Accurancy*: = *max*(*V*.*c*);

16: *iter*: = *iter* + 1;

17: **Until** (*Accurancy* ≥ *Acc*_*Max*) or (*iter* = *Depth*); //When the maximal classification accuracy is obtained or the iteration times is equal to *Depth*, the searching process ends.

18: Select all gene subsets with the highest or nearly highest accuracy and append them into the optimal gene subsets A^*^.

19: **Return** A^*^; //Return the optimal gene subsets A^*^, |A^*^| is the number of the optimal gene subsets, and ∪ A^*^ might be the tumor-related gene set.

Algorithm end

Three stopping criterions are predefined in HBSA:

1) When a gene subset whose accuracy on overall training set is no less than *Acc_Max* threshold is found, the algorithm ends.

2) If no gene subset with *Acc_Max* accuracy is found, the HBSA ends with the maximum iteration times *Depth*, which can guarantee the end of this algorithm. Usually, we do not know how to select an appropriate *Depth.* If *Depth* is set inappropriately, the selected gene subsets might not be optimal.

3) An alternative criterion is that the HBSA ends with the criterion |*Accuracy*_*iter*+1_ − *Accuracy*_*iter*_| < *δ*, where *δ* is set to a very small positive real number and *Accuracy*_*iter*_ denotes the maximum classification accuracy in the *iter*-th iteration.

The most time-consuming operation in the HBSA is to compute *Acc*(*T*). If we assume that computing *Acc*(*T*) only costs one unit time, the time complexity of computing the classification matrix *CM* is *O*(*w* × *p*), and the time complexity for the whole algorithm is *O*(*Depth* × *w* × *p*). Although HBSA is an algorithm of polynomial time complexity, it is still very time-consuming. However, since the task of finding optimal gene subset is mainly performed in laboratory phase and the clinical tumor diagnosis phase only uses the selected gene subsets, which takes only a little CPU time (e.g., within at most several seconds on general PC computer). Thus, our HBSA-based gene selection method is feasible.

### Evaluation criterion

We adopt two machine learning methods, KNN and SVM, to measure the classification accuracy, *Acc*(*T*), of a gene subset *T* in HBSA, respectively. KNN is a common non-parametric method. To classify an unknown sample *x*, KNN extracts the *k* closest vectors from training set by using similarity measures such as Euclidean distance, and decides the label of the unknown sample *x* by using the majority subclass label of the *k* nearest neighbors. *k* is set to an odd number to avoid tied votes. In our experiments Euclidean distance and five nearest neighbors are adopted to measure the similarity of samples and make decisions. The HBSA with KNN is called HBSA-KNN.

SVM [56] with Gaussian Radial Basis Function (RBF) *K*(*xy*) = *exp*(−γ||*x* − *y* ||^2^) (SVM-RBF) is also adopted to evaluate the classification performance of the selected gene subsets. LIBSVM [57] is used in the study, where the combinations of penalized parameter *C* and Gaussian kernel parameter γ need to be optimized when training SVM classifier. Parameter *C* is the penalty factor of the samples classified mistakenly, while parameter γ dominates the sensitivity to the change of input data. Because of the large search space, the general grid-search method (for example, *C* = 2^−5^, 2^−4^, ⋯, 2^15^*γ* = 2^−15^, 2^−14^, ⋯, 2^3^) [58,59] is time-consuming in finding the optimal parameter combinations (*Cγ*). Furthermore, we find that normalized tumor datasets are not sensitive to parameter *C*, and that search space can be reduced with parameter *γ* being set within the range of [10^-5^,10] and *C* being set to 200 and 400 or even fixed to 200. Specifically, if *γ* takes the value in *O*(10^-1^), *γ* may take 0.1, 0.2, ⋯, 0.9, respectively; if *γ* takes the value in *O*(10^-2^), *γ* may take 0.01, 0.02, ⋯, 0.09, respectively. And the others are set similarly. The HBSA with SVM is called HBSA-SVM.

The *k*-fold Cross-Validation (*k*-fold CV) is commonly used to evaluate classification model. Here it is applied only on training set to measure *Acc*(*T*). If *k* is set to *Tr*_*n*_ (the size of training set), the *k*-fold CV is called Leave-One-Out Cross-Validation (LOOCV). If *k* is set to 2, the *k*-fold CV is known as the holdout method. When *k* is set too low, the accuracy of *k*-fold CV tends to have high bias and low variance. On the contrary, when *k* is set too high (e.g., *k = Tr*_*n*_), the accuracy of *k*-fold CV will have low bias but high variance [51,60]. Breiman *et al.*[61] found that 10-fold CV method outperforms the LOOCV method to some extent. Ambroise *et al.*[45] and Asyali *et al.*[52] also recommended 10-fold CV methods in tumor classification, but whether 10-fold CV method outperforms LOOCV method depends on datasets. To balance the bias and variance, here we design a new method to evaluate the experimental results. Let *CV*(*k*) denote the accuracyof *k*-fold CV classification, where 2 ≤ *k* ≤ *m* and *m* is the total number of samples in training set. Then the mean of the accuracy is defined as:

(5) $$mean=\frac{1}{m-1}(\sum_{k=2}^{m}CV(k))$$

The standard deviation is defined as:

(6) $$std=\sqrt{\sum_{k=2}^{m}{\left(CV\left(k\right)-mean\right)}^{2}/(m-2)}$$

This method is called Full-fold CV method. The mean of the accuracy evaluated by this method is called Full-fold CV accuracy. Since the computational cost of HBSA would greatly increase by using Full-fold CV to compute *Acc*(*T*), 10-fold CV is still used to evaluate *Acc*(*T*) as the heuristic information of HBSA. While Full-fold CV method is only used to evaluate the resultant gene subsets in A^*^ with the highest or nearly highest 10-fold CV accuracy.

The implementation of HBSA-KNN is similar, but different in some ways, to that of HBSA-SVM. For HBSA-KNN, we randomly divide training set into 10 parts when using 10-fold CV method, but different divisions can slightly affect the experimental results. To eliminate the effects of different divisions, HBSA-KNN is performed five times with different divisions of training set, thus we could obtain five optimal sets A^*^. Then the occurrence frequency of each gene is counted from the obtained five optimal sets A^*^. However, for HBSA-SVM, the division of training set for 10-fold CV method, provided by LIBSVM, is definite in each run. It is sufficient to perform HBSA-SVM only once.

Usually, for HBSA-SVM, final prediction accuracy is evaluated on independent test set by SVM-RBF classifier constructed by optimizing parameter pair only on training set, which is called HBSA-SVM (Unbiased). However, more than one parameter pairs can make the constructed classifiers obtain the highest 10-fold CV accuracy on training set, while the classifiers constructed with these different parameter pairs obtain different prediction accuracy on independent test set. So, in contrast with HBSA-SVM (Unbiased), a biased HBSA-SVM, selecting the parameter pair that makes the constructed classifier obtain the highest prediction accuracy on test set, is also used to evaluate the performance of the selected gene subsets, which is called HBSA-SVM (Biased).

Receiver Operator Characteristics (ROC) analysis is a visual method for evaluating the performance of binary classification model [62].Usually, a few performance measures can be derived from the number of true positives (TP), true negatives (TN), false positives (FP) and false negatives (FN) in test set to measure the performance of classification model, i.e., the true-positive rate or sensitivity (TPR), the false-positive rate(FPR), positive predictive value(PPV), and negative predictive value(NPV). Here ROC curve that is a TPR (on the *y*_*axis*) versus FPR (on the *x*_*axis*) plot is used, and the Area Under ROC Curve (AUC) is used to measure the performance of classification model.

(7) Acc=accurancy=(TP+TN)/(TP+TN+FP+FN)

(8) SP=specificity=TN/(FP+TN)

(9) TPR=sensitivity=TP/(TP+FN)

(10) FPR=(1−specificity)=FP/(FP+TN)

(11) PPV=TP/(TP+FP)

(12) NPV=TN/(TN+FN)

### Analysis framework

#### Flowchart of analysis

**Figure 3:**
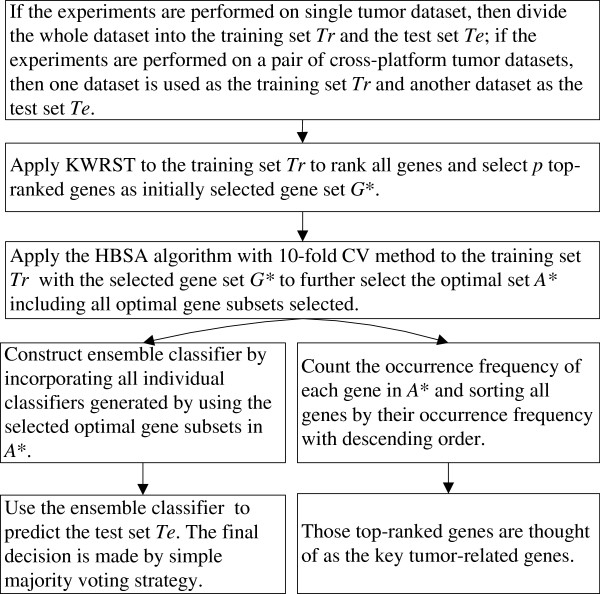
The flowchart of our analysis method.

After HBSA is applied to gene selection from the differentially expressed genes initially selected by KWRST on training set, numerous optimal gene subsets are obtained. However, finding optimal gene subsets in such tremendous gene space tends to over-fit training set. Some tumor-unrelated genes are very likely to be selected mistakenly into optimal gene subsets, which might introduce serious bias in the gene selection. The generalization performance of these gene subsets containing tumor-unrelated genes is possibly very poor in predicting unknown tumor samples. To address this problem, we design a HBSA-based ensemble classifier and a HBSA-based gene ranking method to obtain unbiased prediction accuracy and find as many important tumor-related genes as possible. The flowchart of our analysis method is shown in Figure 3.

#### HBSA-based ensemble classifier

**Figure 4:**
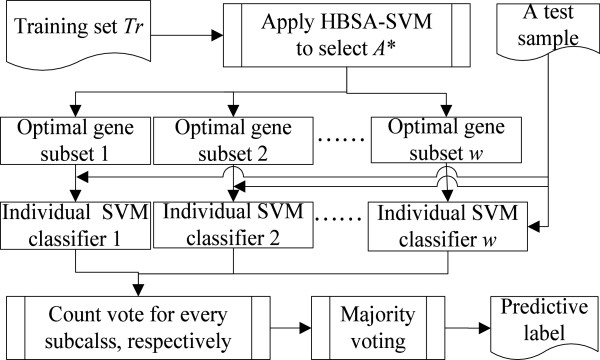
The construction of HBSA-SVM-based ensemble classifier.

The HBSA-based ensemble classifier consists of the individual classifiers constructed from the optimal gene subsets, and the corresponding prediction accuracies (Biased and Unbiased) are determined by the ensemble classifiers constructed by SVM (Biased) and SVM (Unbiased) on test set, respectively. Final decisions are made by simple majority voting strategy in our experiments. To illustrate the results, the construction of an ensemble SVM classifier with *w* individual SVM classifiers is shown in Figure 4, where each individual SVM classifier is constructed by each optimal gene subset *T* obtained by HBSA-SVM.

To measure the reliability of the classification for each test sample by the ensemble classifier constructed with *N* individual classifiers, a confidence level is defined. Assume that a dataset has *k* subclasses denoted by *L* = {*c*_1_, ⋯, *c*_*k*_}, a test sample is assigned a voting vector (*m*_1_, ⋯, *m*_*k*_), where each component *m*_*i*_ denotes the number of the obtained votes for the corresponding subclasses *c*_*i*_ in *L* = {*c*_1_, ⋯, *c*_*k*_}, where $\sum_{i=1}^{k}m_{i}=N$. Let *m*_*max*_ and *m*_*sec*_ denote the maximum and next maximum number in voting vector (*m*_1_, ⋯, *m*_*k*_), respectively. The confidence level *conf* of a test sample can be defined as *conf* = *m*_*max*_/*m*_*sec*_. If *m*_*sec*_ = 0, *conf* is set to *N*, where 1 ≤ *conf* ≤ *N*. The bigger the *conf* is, the more reliably is the test sample correctly or mistakenly classified.

#### HBSA-based gene ranking method

The HBSA-based gene ranking method, which ranks genes according to the occurrence frequency count of each gene in the final optimal gene subsets A^*^, is designed to find important tumor-related genes. That is, the significance of a gene is measured by its occurrence frequency. The top-ranked genes with the highest occurrence frequency are considered to be the most important tumor-related ones and should have superior and robust generalization performance.

## Results

### Tumor datasets

Nine publicly available tumor datasets are applied: Small Round Blue Cell Tumor (SRBCT) [10], Acute Lymphoblastic Leukemia (ALL) [63], Colon tumor [9], Leukemia72 [2], Leukemia52 [64], Diffuse Large B-cell Lymphomas (DLBCL77) [11], DLBCL21 (obtained from R. Dalla-Favera’s lab at Columbia University) [65], Prostate102 [12], and Prostate34 [66] datasets. Among these datasets, three pairs of cross-platform datasets are used to evaluate the generalization performance for our classification model. The division of training set and test set is shown in Table 1. More details about the datasets are available in the Additional file 1: Tables S1-S4. 

**Table 1:** Designation of training set and test set in our experiments

**Datasets**	**Usages**	m*	n*	***k***	**Platform**
Prostate102	Tr**	102	12,600	2	Affy HU95A V2
Prostate34	Te**	34	12,626	2	Affy U95A
DLBCL77	Tr	77	7,129	2	Affy HU6800
DLBCL21	Te	21	12,581	2	Affy HU95AV2
Leukemia72	Tr	72	7,129	2	Affy HU6800
Leukemia52	Te	52	12,582	2	AffyHGU95a
Colon	Tr	42	2000	2	AffyHUM6000
	Te	20			
ALL	Tr	148	12626	6	Affy HGU95AV2
	Te	100			
SRBCT	Tr	63	2308	4	cDNA
	Te	20			

* *m* denotes the number of sample in dataset. *n* denotes the number of genes, and *k* denotes the number of subclasses.

**“Tr” denotes this dataset will be used as training set, while “Te” denotes this dataset will be used as test set.

### HBSA-SVM classification performance

The gene selection procedure of HBSA-SVM is performed only on training set. Considering the computational performance of our computer, we initially select 300 top-ranked genes by KWRST. Then training sets and test sets are normalized by genes using z-score normalization method that makes dataset with mean zero and standard deviation one, respectively. Other parameters in HBSA are set: *p* = 300, *w* = 300, *Acc*_*Max* = 100, and *Depth* = 15, respectively. After the experiments are performed on six training sets, for each dataset 300 optimal gene subsets are selected according to 10-fold CV method. Part of the optimal gene subsets selected are shown in Table 2, which shows that at least one gene subset with 100% training accuracy is always obtained for each tumor dataset. It is also found that the prediction accuracy of HBSA-SVM (Unbiased) is always not greater than that of HBSA-SVM (Biased). The experiments further indicate that searching optimal gene subsets costs high computationally. For example, for the ALL dataset, it costs about 11 days by using HBSA-SVM at the worst case on our computational platform of Core (TM) 2 Duo 2.20 GHz CPU and 2 G RAM.

**Table 2:** Representative results obtained by the HBSA-SVM(Biased) and HBSA-SVM(Unbiased)

**Dataset**	**No.**	**Optimal gene subsets selected by the HBSA on training set**	**10-Fold CV % on training set**	**Full-fold CV % on training set**	**Prediction Acc.% on test set (Biased)**	**Prediction Acc.% on test set (Unbiased)**
Leukemia	1	{M23197, M31523}	100	98.75 ± 0.42	86.54	86.54
	2	{M23197, Y07604}	100	99.41 ± 0.69	80.77	73.08
	3	{M23197, U46751}	100	99.96 ± 0.33	80.77	73.08
	4	**{X95735, Y07604}**	**100**	**99.96 ± 0.23**	**73.08**	**71.15**
	5	{M31523, L47738}	100	99.22 ± 0.73	88.46	71.15
	6	**{M63379, Z15115}**	**100**	**99.86 ± 0.82**	**94.23**	**92.31**
DLBCL	1	{U28386, U81375, D78134}	100	100 ± 0	90.48	76.19
	2	**{U28386, U90313, D78134}**	**100**	**100 ± 0**	**76.19**	**71.43**
	3	{X67951, L06132, D78134}	100	100 ± 0	80.95	76.19
	4	{U81375, L06132, D78134}	100	100 ± 0	95.24	90.48
	5	{L06132, L35249, D78134}	100	99.86 ± 0.58	85.71	85.71
	6	**{L06132, D78134, Z35227}**	**100**	**100 ± 0**	**100**	**85.71**
Prostate	1	{37639_at, 41504_s_at, 40074_at, 1708_at}	100	99.96 ± 0.24	91.18	76.47
	2	**{37639_at, 41504_s_at, 863_g_at, 32225_at}**	**100**	**100 ± 0**	**91.18**	**88.24**
	3	{41288_at, 38087_s_at, 41504_s_at, 32786_at}	100	99.99 ± 0.10	88.24	82.35
	4	**{37639_at, 41504_s_at, 34853_at, 863_g_at}**	**100**	**99.07 ± 0.21**	**85.29**	**82.35**
SRBCT	1	{770394, 769716, 563673}	100	99.90 ± 0.39	80	75
	2	{859359, 1435862, 769716}	100	99.80 ± 0.73	90	85
	3	{377461, 769716, 563673}	100	99.97 ± 0.20	85	75
	4	{859359, 377461, 782193}	100	99.72 ± 0.93	85	75
	5	{1435862, 143306, 782193}	100	99.72 ± 1.17	80	65
	6	**{859359, 769716, 134748}**	**100**	**97.44 ± 1.30**	**60**	**40**
	7	{1435862, 207274, 878652}	100	99.97 ± 0.20	90	80
	8	**{295985, 769716, 221826}**	**100**	**98.50 ± 1.53**	**95**	**85**
	9	{308231, 214572, 784257}	100	99.90 ± 0.63	75	65
	10	{1435862,383188,141768}	100	94.88 ± 1.59	70	65
ALL	1	{AF068180,L13939,AF041434,M64925,X17025,J03473}	100	99.94 ± 0.27	96	95
	2	{M11722,AF013249,Z50022,X17025,J03473,U03106}	100	99.98 ± 0.12	95	94
	3	{M11722,AF013249,X17025,J03473,U03106,AB018310}	100	99.99 ± 0.08	94	92
	4	**{M11722,X17025,J03473,AB007902,U46922,AI525834}**	**100**	**99.83 ± 0.43**	**91**	**86**
	5	**{M11722,X17025,J03473,U46922,AI525834,U51240}**	**100**	**99.99 ± 0.08**	**96**	**92**
Colon	1	**{M26383, H40095}**	**100**	**100 ± 0**	**70**	**65**
	2	{M26383, R84411}	100	99.94 ± 0.37	80	65
	3	**{D21261, H20709}**	**100**	**97.97 ± 0.85**	**85**	**85**
	4	{J05032, M76378}	100	99.65 ± 1.14	70	65
	5	{J05032, M63391}	100	99.71 ± 0.95	75	70

Over-fitting occurs in selecting gene subsets on all six training sets as shown in Additional file 1: Table S2. For example, for the leukemia dataset, 2-gene subset {X95735, Y07604} with 100% training accuracy has only 73.08% prediction accuracy on Leukemia52. For SRBCT, 3-gene subset {859359, 769716, 134748} with 100% training accuracy obtained only 60% prediction accuracy. Some gene subsets may obtain very high prediction accuracy (e.g., for DLBCL77, 3-gene subset {L06132, D78134, Z35227} with 100% training accuracy also obtains 100% prediction accuracy on DLBCL21). It may be only by chance to find such gene subsets because the high training accuracy obtained by this gene subset cannot represent good generalization performance of the obtained classifier due to the biased gene selection procedure on whole training set. In fact, some gene subsets with nearly 100% training accuracy obtaining very high prediction accuracy on test set indicate that these gene subsets probably contain important tumor-related genes. For example, for the leukemia dataset, two genes {X95735, M63838} with only 97.22% training accuracy can obtain 96.15% prediction accuracy on Leukemia52, in which X95735 is an important tumor-related gene. Moreover, some redundant or noise genes may potentially degrade the classification performance by masking the contribution of the relevant genes. For example, for SRBCT, 2-gene subset {859359, 769716} can obtain 70% prediction accuracy on the corresponding test set, but 3-gene subset {859359, 769716, 134748} only obtains 60% prediction accuracy. Similarly, HBSA-KNN can also lead to over-fitting phenomena.

The genes in the same gene subset usually come from different pathways. For instance, for the ALL dataset, the six genes in gene subset {BLNK(AF068180), AP1B1(L13939), PTP4A3(AF041434), MPP1(M64925), IDI1(X17025), PARP1(J03473)}with 100% training accuracy take part in different pathways. BLNK takes part in base excision repair and B cell receptor signaling pathways. PARP1 takes part in the primary immunodeficiency pathway. For the gene subset {DNTT(M11722), LAIR1(AF013249), PTTG1IP(Z50022), IDI1(X17025), PARP1(J03473), CDKN1A(U03106)}, although these genes are enriched in 12 important pathways, there are no two genes taking part in the same pathway. For the SRBCT dataset, the 3-gene subset {CD99 (1435862), RCVRN(383188),ERBB2(141768)}are involved in 11 major pathways, but all these three genes come from different pathways. The genes in subset {CDK6(295985), NF2(769716),GNA11(221826)} participate in 13 important pathways such as non-small cell lung cancer, p53 signaling pathway, etc., but there are no two genes in the gene subset on the same pathway. In addition, we find that the majority of the genes selected are involved in important tumor-related biological pathways. For example, the gene CDK6 is involved in non-small cell lung cancer, p53 signaling pathway, Melanoma, etc., in total 9 pathways. Thus, the results are generally consistent with our interpretation of HBSA.

### Ensemble classifier

#### HBSA-SVM-based ensemble classification

To solve the above over-fitting problem, HBSA-SVM-based ensemble schemes are constructed by using a simple majority voting strategy to integrate the individual classifiers. The number of the gene subsets used to construct an ensemble classifier is determined by experiments. The results of three different ensemble classifiers based on different modes are shown in Table 3. For example, for the leukemia dataset, the item Top 300 gene subsets, 300 top-ranked gene subsets with the highest training accuracy are selected, but only 147 gene subsets among the 300 gene subsets share with the Leukemia52 test set. Thus the final ensemble classifier consists of the 147 individual classifiers respectively constructed from these 147 common gene subsets. The corresponding prediction accuracies (Biased and Unbiased) are obtained on the Leukemia52 test set, respectively.

**Table 3:** Prediction accuracies of the ensemble SVM(Biased) and SVM(Unbiased) classifiers

**Dataset**	**Ensemble modes**	**#Individual**	**Acc.%**	**Acc.%**
		**classifiers**	**(Biased)**	**(Unbiased)**
**Leukemia**	Top 300 gene subsets	147	92.31	84.62
	10-Fold >98*	47	96.15	88.46
	10-Fold = 100 and Full-fold > =99	5	88.46	86.54
**DLBCL**	Top 300 gene subsets	61	95.24	85.71
	10-Fold = 100	143**	95.24	85.71
	10-Fold = 100 and Full-fold = 100	29**	95.24	85.71
**Prostate**	Top 300 gene subsets	300	97.06	88.24
	Full-fold > 98	290	97.06	88.24
	Full-fold > 99	139	97.06	88.24
**SRBCT**	Top 300 gene subsets	300	90	80
	Full-fold > 98	114	95	85
	Full-fold > 98 and 10-Fold = 100	8	100	90
**ALL**	Top 300 gene subsets	300	96	96
	10-Fold = 100	59	97	96
	10-Fold = 100 and Full-fold > =99	42	95	95
**Colon**	Top 300 gene subsets	300	90	70
	10-Fold = 100	62	85	65
	10-Fold = 100 and Full-fold > =98	59	85	65

* The corresponding prediction accuracies (Biased and Unbiased) are obtained on the Leukemia52 test set, respectively. The item 10-Fold > 98 means that the gene subsets with 10-fold CV accuracy greater than 98% are selected from the 300 top-ranked gene subsets in which only 47 gene subsets are shared between the Leukemia72 training set and Leukemia52 test set. Thus the final ensemble classifier consists of the 47 individual classifiers respectively constructed from these 47 gene subsets; the corresponding prediction accuracies (Biased and Unbiased) are obtained by the ensemble classifiers constructed by SVM(Biased) and SVM(Unbiased) on the Leukemia52 test set, respectively.

** The individual classifiers are constructed from the gene subsets that are selected from all nodes in last layer, not limited to the 300 top-ranked nodes in last layer because more than 300 gene subsets can obtain 100% 10-fold CV accuracy on DLBCL.

To analyze the reliability of classification, the confidence level for each sample is calculated. Taking the colon tumor dataset as an example, the confidence levels of 20 test samples are shown in Table 4 by HBSA-SVM(Unbiased), in which the 7th, 9th and 13thsamples are mistakenly classified with very high confidence levels, 3.3478, 3.1096 and 99, respectively. Compared with the results in Additional file 1: Table S30 obtained by HBSA-SVM(Biased), most of the samples mistakenly classified in Table 4 are the ones mistakenly or correctly classified with low confidence levels in Additional file 1: Table S30.

**Table 4:** Confidence levels of 20 test samples by HBSA-SVM(Unbiased)-based ensemble classifier on colon tumor dataset

**20 samples**	**#Tumor subclass votes**	**#Normal subclass votes**	**Confidence level**	Correct? **
(No.) *				
1 (43)	116	184	1.5862	C
2 (44)	298	2	149	C
3 (45)	111	189	1.7027	**E**
4 (46)	285	15	19	C
5 (47)	286	14	20.4286	C
6 (48)	119	181	1.5210	C
7 (49)	69	231	3.3478	**E**
8 (50)	165	135	1.2222	**E**
9 (51)	227	73	3.1096	**E**
10 (52)	297	3	99	C
11 (53)	276	24	11.5	C
12 (54)	19	281	14.7895	C
13 (55)	297	3	99	**E**
14 (56)	88	212	2.4091	**E**
15 (57)	193	107	1.8037	C
16 (58)	230	70	3.2857	C
17 (59)	260	40	6.5	C
18 (60)	98	202	2.0612	C
19 (61)	300	0	300	C
20 (62)	118	182	1.5424	C

* The number in parentheses denotes the serial number of sample in original colon tumor dataset.

** “C” means the sample classified correctly and “E” means the sample classified mistakenly.

#### HBSA-KNN-based ensemble classification

HBSA-KNN-based ensemble classifier is also constructed by using majority voting strategy to combine 300 individual classifiers constructed by 300 optimal gene subsets selected by HBSA-KNN. Unlike HBSA-SVM, for each dataset random division of 10-fold CV on training set are run for five times, and the average of the five accuracies is used as the final prediction accuracy. For the cross-platform datasets, only the gene subsets shared between the training set and the corresponding test set within the selected 300 gene subsets are used to construct an ensemble classifier. The prediction accuracies of the constructed ensemble KNN classifier are listed in Table 5. Compared with prediction accuracies obtained by the ensemble HBSA-SVM(Unbiased) classifier, shown in Table 3, the prediction accuracy of the ensemble KNN classifier is no less than that of the ensemble SVM(Unbiased) classifier except the prostate dataset.

**Table 5:** Prediction accuracies of five runs of HBSA-KNN-based ensemble classifier on six test sets

**Run**	**First**	**Second**	**Third**	**Fourth**	**Fifth**	**Average**
**Dataset**	**Acc.%**	**Acc.%**	**Acc.%**	**Acc.%**	**Acc.%**	**Acc.%**
Leukemia	86.54	84.62	88.46	84.62	84.62	85.57 ± 1.66
DLBCL	90.48	90.48	90.48	85.71	90.48	89.53 ± 2.13
Prostate	85.29	82.35	85.29	85.29	85.29	84.70 ± 1.31
SRBCT	95	95	95	90	95	94 ± 2.24
ALL	95	97	96	95	95	95.60 ± 0.89
Colon	75	75	75	75	75	75 ± 0

Column First denotes the prediction accuracy of the constructed ensemble classifier obtained on the first run of the HBSA-KNN, and the others are deduced by analogy. The average accuracy is the average prediction accuracy obtained by five runs of the HBSA-KNN.

### HBSA-based gene ranking

To prioritize genes so as to find important tumor-related genes, we simply count the occurrence frequency of each gene in all of the optimal gene subsets to measure the gene significance. The 50 top-ranked genes selected by HBSA-SVM and HBSA-KNN for each dataset are shown in Additional file 1: Tables S5-S10 and S17-S22, respectively. It is shown that only few genes have relatively higher frequency, and that the respective top 10 genes selected by HBSA-SVM and HBSA-KNN are mostly shared on the same dataset. The result suggests that our HBSA-based gene ranking method is robust and valid.

We also find that the most frequently selected genes are not always the most differentially expressed ones. For DLBCL, MCM7 that is ranked the first by KWRST is ranked the third in the corresponding list of gene frequency by HBSA-SVM. However, RHOH that is ranked the first in the frequency list by HBSA-SVM is ranked the 258-th by KWRST. However, for SRBCT, most of the top 10 genes selected by HBSA-SVM are included in the top 10 genes selected by KWRST, suggesting that the most differentially expressed genes in this dataset are the most important tumor-related genes. Therefore the most important tumor-related genes are not necessarily the most differentially expressed ones.

**Figure 5:**
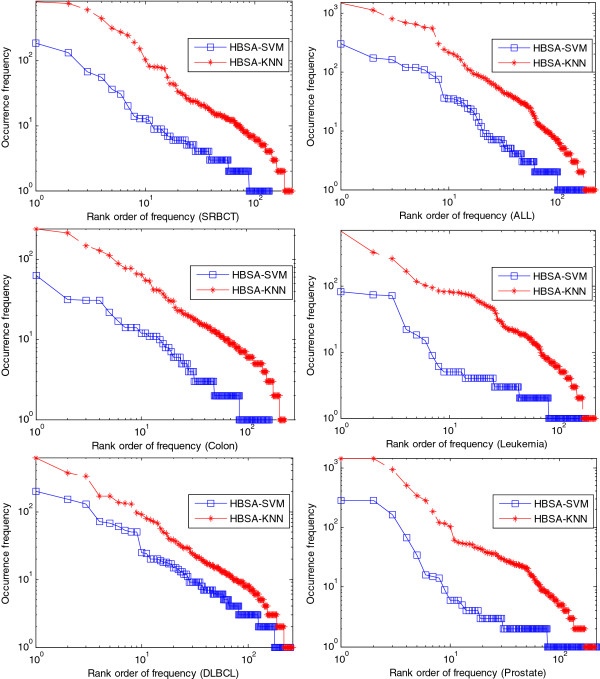
**Power-law distribution of the occurrence frequency of genes selected on six tumor datasets.** The abscissa denotes the frequency rank order of the selected genes. The vertical axis denotes the occurrence frequency of genes selected. The figure is drawn by using log-log coordinates.

Figure 5 shows the relationship between the occurrence frequency of genes and their rank orders. An important aspect of the occurrence frequency of gene in Figure 5 is the linearity of the log-log plots, so it can be inferred that the occurrence frequency of the selected genes follows power-law distribution with respect to the number of genes whose frequencies are greater than the corresponding frequency. This discovered trend is consistent with a previous study [67]. The gene frequency of HBSA-KNN is the accumulated frequency of gene from five runs of HBSA-KNN for each dataset, which indicates the characteristic of rich-get-richer. 

**Figure 6:**
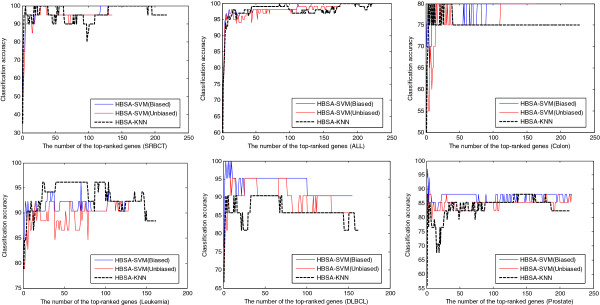
Classification accuracy versus the number of top-ranked genes on the six test sets.

Figure 6 shows that the classification accuracy varies with the number of top-ranked genes sorted by the gene frequencies for HBSA-SVM(Biased), HBSA-SVM(Unbiased) and HBSA-KNN, respectively. Table 6 lists the prediction accuracies of some representative number of top-ranked genes selected by HBSA-SVM(Biased), HBSA-SVM(Unbiased) and HBSA-KNN on independent test sets. It is found that a few top-ranked genes are enough for achieving the highest or nearly highest classification accuracy. Moreover, the prediction accuracy of HBSA-KNN is comparable to HBSA-SVM(Unbiased). For example, for HBSA-KNN on SRBCT, five genes can obtain 100% prediction accuracy, while 28 genes are needed to obtain the same accuracy by HBSA-SVM(Unbiased). High accuracy obtained with few genes could be more objective and reliable than that with much more genes since the latter easily leads to classification bias [68]. Interestingly, for HBSA-SVM and HBSA-KNN, when the number of the top-ranked genes approximates the number of subclasses in dataset, the prediction accuracy of the classification model constructed by these gene subsets can achieve similar performance or even outperform that of the corresponding ensemble classifier. 

**Table 6:** Comparison of the classification accuracies for HBSA-SVM(Biased), HBSA-SVM(Unbiased) and HBSA-KNN methods with the top-ranked genes

**Dataset**	**HBSA-SVM (Biased)**		**HBSA-SVM (Unbiased)**		**HBSA-KNN**	
	#TG*	**Acc.%**	#TG*	**Acc.%**	#TG*	**Acc.%**
**Leukemia**	2	88.46	3	82.69	2	84.62
	**3**	**92.31**	5	86.54	**5**	**90.38**
	71	96.15 (H)	15	92.31 (H)	24	96.15 (H)
**DLBCL**	2	95.24	2	80.95	2	80.95
	**3**	**100** (H)	**9**	**95.24** (H)	**3**	**90.48** (H)
**Prostate**	1	94.12	**1**	**91.18** (H)	**2**	**88.24** (H)
	**2**	**97.06** (H)	4	88.24	5	85.29
**SRBCT**	4	75	4	70	3	75
	**5**	**95**	**5**	**90**	**4**	**95**
	24	100 (H)	28	100 (H)	**5**	**100** (H)
**ALL**	**6**	**97**	**6**	**96**	**5**	**94**
	7	96	**10**	**97**	**9**	**96**
	112	100 (H)	111	99 (H)	85	100 (H)
**Colon**	**2**	**75**	2	65	3	70 (H)
	**3**	**80** (H)	7	70	**4**	**80** (H)
	4	80	**15**	**80**(H)	7	80

*‘#TG’ denotes the number of top-ranked genes. Note that the accuracy labeled by ‘H’ denotes the highest accuracy and the number of the corresponding top-ranked genes denotes the minimal number with the highest accuracy.

### Comparison of HBSA-KNN and HBSA-SVM

**Figure 7:**
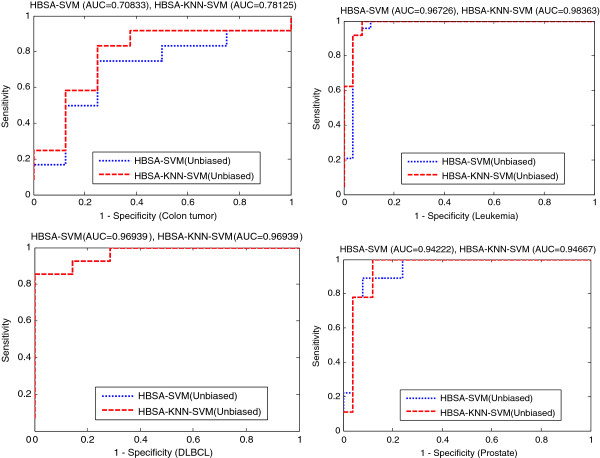
ROC comparisons of HBSA-SVM(Unbiased) and HBSA-KNN-SVM(Unbiased).

The ensemble HBSA-KNN classifier slightly outperforms ensemble HBSA-SVM(Unbiased) classifier in prediction accuracy. Further comparison of the prediction accuracies of HBSA-SVM(Biased), HBSA-SVM(Unbiased) and HBSA-KNN varying with different number of top-ranked genes is shown in Figure 6. The comparison indicates that HBSA-KNN is slightly superior to HBSA-SVM(Unbiased) in prediction accuracy when the number of top-ranked genes selected is small enough. To further reveal how the biased SVM affect classification results, the gene list obtained by HBSA-KNN is further evaluated by SVM-RBF classifier constructed by biased and unbiased methods again, respectively (Additional file 1: Figure S2).The two methods are named HBSA-KNN-SVM(Biased) and HBSA-KNN-SVM(Unbiased), respectively. Additional file 1: Figure S2 shows that HBSA-KNN-SVM(Unbiased) can always obtain the prediction accuracy no greater than that of HBSA-KNN-SVM(Biased) and that HBSA-KNN is also slightly superior to HBSA-KNN-SVM(Biased) in prediction accuracy when the number of top-ranked genes is small. To further evaluate the effectiveness of HBSA-KNN in gene selection, the eight top-ranked genes are selected to construct HBSA-SVM(Unbiased) and HBSA-KNN-SVM(Unbiased) classifiers on four binary datasets, respectively, which are evaluated by ROC (Figure 7). It is clear that HBSA-KNN-SVM(Unbiased) is slightly superior to HBSA-SVM(Unbiased) in AUC, indicating that the classification ability of the gene subsets selected by HBSA-KNN is slightly stronger than that obtained by HBSA-SVM.

### Comparisons with other related methods

Compared with the exhaustive search method, proposed by Wang L.P. *et al.*[46], our methods are less computationally demanding. Moreover, the ensemble strategy adopted is also superior to their average strategy which averages the prediction accuracy of all gene subsets selected from training set. Coincidently, the 3-gene subsets {IGF2, AF1q(MLLT11), CD99},selected by exhaustive search method [46], with 95% prediction accuracy is identical to the first three genes selected by our HBSA-KNN (see Additional file 1: Table S17), which indicates that our HBSA is feasible and can achieve the same good results as the exhaustive search method. The Prediction Analysis of Microarrays (PAM) proposed by Tibshirani *et al.*[22] can identify a small subset of genes that best characterize each subclass by shrinking weak components of class-centroids with a shrinkage parameter for tumor subclass prediction. Its experimental results on SRBCT and leukemia datasets demonstrated that their method is very efficient in finding informative genes with high classification accuracy. Of the 43 genes selected by PAM on SRBCT dataset, 21 genes are also found by our method on the same dataset (where only the 50 top-ranked genes are considered as shown in Additional file 1: Table S5). On the other hand, although one of their goals was to find the smallest gene subsets, the size of their selected gene subsets with satisfactory accuracy was still too large from the viewpoint of classification and clinical diagnosis.

In addition, Dabney *et al.*[69,70] proposed a Classification to Nearest Centroids (ClaNC) method for class-specific gene selection. To find the theoretically optimal gene subset, they further provided a theoretical result showing how to determine the gene subsets of a given size that maximizes the classification accuracy for high-dimensional nearest centroid classifiers. Their results suggest that ClaNC outperforms PAM in prediction accuracy. However, before gene selection, ClaNC requires a given number of genes, which is difficult to determine how many genes are appropriate.

Our method is similar to PAM and ClaNC methods in three aspects. 1) Find minimum gene subsets with maximum accuracy. 2) Consider the discriminative power of multiple genes when searching for gene subsets. 3) Seek the simplest method with biomedical interpretability.

To achieve more objective comparison, the classification performance of PAM, ClaNC and our method are obtained on the two cross-platform datasets (leukemia and DLBCL) that are realigned by those shared genes between the training set and the corresponding test set, respectively. For the leukemia dataset, 4606 genes are shared between Leukemia72 and Leukemia52. For DLBCL, 4072 genes are shared between DLBCL77 and DLBCL21.

Since HBSA-KNN is slightly superior to HBSA-SVM(Unbiased) in gene selection, we just compare HBSA-KNN with PAM and ClaNC methods in prediction accuracy. The conclusion from the comparisons of the classification accuracy, shown in Table 7, is that although ClaNC outperforms PAM in accuracy, the accuracy obtained by ClaNC is lower than ours on all six independent test sets when the number of the top-ranked genes selected is small enough, i.e., when the size of the selected gene subset approximately satisfies (4) as shown in Table 7. For example, for ALL (six subclasses), when the number of the top-ranked genes selected by HBSA-KNN is five, 94% prediction accuracy can be obtained, while ClaNC obtains only 86% accuracy with six genes (one gene selected per subclass). For SRBCT (four subclasses), our method obtain 100% prediction accuracy with only five genes, while eight genes (two genes selected per subclass) are needed to obtain 95%prediction accuracy by ClaNC. For the prostate dataset (two subclasses), our method obtains 88.24% accuracy with two genes, while only 74% accuracy is obtained by ClaNC with the same number of genes. Obviously, our method can achieve higher accuracy with the same or fewer top-ranked genes. From Table 7 we can see that the PAM method does not performs well in the classification of some cross-platform datasets because the same accuracy is obtained when different number of genes for the DLBCL and Prostate cross-platform datasets are used, which are possibly caused by the fact that the cross-platform training set and test set are not on the same measurement scale.

**Table 7:** The comparison of prediction accuracies by HBSA-KNN, PAM and ClaNC on independent test set

**Methods**	**Dataset**	**Number of the top-ranked genes**									
		**2**	**3**	**4**	**5**	**6**	**7**	**8**	**20**	**40**	**60**
HBSA-KNN	Leukemia (*Acc*)	84.62	94.23	92.31	94.23	82.69	82.69	82.69	88.46	90.38	92.31
	*sensitivity*	100	100	100	100	100	100	100	100	100	100
	*specificity*	71.43	89.29	85.71	89.29	67.86	67.86	67.86	78.57	82.14	85.71
	*PPV*	75	88.89	85.71	88.89	72.73	72.73	72.73	80	82.76	85.71
	*NPV*	100	100	100	100	100	100	100	100	100	100
	DLBCL (*Acc*)	95.24	100	80.95	85.71	80.95	85.71	85.71	85.71	85.71	90.48
	*sensitivity*	100	100	85.71	85.71	78.57	85.71	85.71	85.71	85.71	92.86
	*specificity*	85.71	100	71.43	85.71	85.71	85.71	85.71	85.71	85.71	85.71
	*PPV*	93.33	100	85.71	92.31	91.67	92.31	92.31	92.31	92.31	92.86
	*NPV*	93.33	100	85.71	92.31	91.67	92.31	92.31	92.31	92.31	92.86
	Prostate (*Acc*)	88.24	76.47	82.35	85.29	82.35	79.41	76.47	76.47	82.35	85.29
	*Sensitivity*	100	100	100	100	100	88.89	88.89	88.89	88.89	100
	*Specificity*	84	68	76	80	76	76	72	72	80	80
	*PPV*	69.23	52.94	60	64.29	60	57.14	53.33	53.33	61.54	64.29
	*NPV*	100	100	100	100	100	95	94.74	94.74	95.24	100
	SRBCT	75	95	95	100	95	95	95	95	95	100
	ALL	75	82	87	94	92	93	93	96	97	99
	Colon (*Acc*)	65	70	80	75	80	80	75	75	75	75
	*sensitivity*	75	83.33	91.67	91.67	91.67	83.33	83.33	83.33	75	75
	*specificity*	50	50	62.50	50	62.50	75	62.50	62.50	75	75
	*PPV*	69.23	71.43	78.57	73.33	78.57	83.33	76.92	76.92	81.82	81.82
	*NPV*	57.14	66.67	83.33	80	83.33	75	71.43	71.43	66.67	66.67
PAM	**Dataset**	**Number of the selected genes**									
		**2**	**4**	**6**	**8**	**10**	**12**	**16**	**20**	**40**	**60**
	Leukemia	82.69	90.38	90.38	90.38	92.31	94.23	96.15	96.15	98.08	98.08
	DLBCL	66.67	66.67	66.67	66.67	66.67	66.67	66.67	66.67	66.67	66.67
	Prostate	73.53	73.53	73.53	73.53	73.53	73.53	73.53	73.53	73.53	73.53
	SRBCT	45	45	75	75	85	95	95	95	95	95
	ALL	43	61	61	68	68	83	85	85	86	86
	Colon	65	75	70	70	70	75	75	75	75	75
ClaNC	**Dataset**	**Number of the selected genes**									
		1 × k*	**2 × *k***	**3 × *k***	**4 × *k***	**5 × *k***	**6 × *k***	**7 × *k***	**8 × *k***	**9 × *k***	**10 × *k***
	Leukemia	86.54	90.39	90.39	92.31	90.39	94.23	94.23	94.23	94.23	96.15
	DLBCL	80.95	95.24	95.24	95.24	95.24	80.95	76.19	71.43	71.43	71.43
	Prostate	73.53	85.29	79.41	76.47	76.47	79.41	79.41	76.47	76.47	79.41
	SRBCT	85	95	95	95	95	95	95	95	95	95
	ALL	86	95	97	99	98	98	99	99	99	98
	Colon	65	65	65	70	70	75	75	75	75	75

* *k* denotes the number of subclasses in each dataset, which ranges from two to six. For example, for ALL dataset, the size of the gene subset selected ranges from six (1 × 6) to sixty (10 × 6).

Note that the prediction accuracy may be affected by different data normalization methods. The results in Table 7 are obtained with the z-score normalization method on the tumor datasets. If we use another 0–1 normalization method that scales all data into the range of [0, 1] with the formula (*x* − *min*(*x*))/(*max* (*x*) − *min*(*x*)), where *x* is a vector that denotes a set of expression values of a gene in different samples, the results may vary with the same gene subset as shown in Table 7 and Additional file 1: Table S29. For example, for the leukemia dataset, the first three genes obtain 94.23% prediction accuracy on the Leukemia52 test set with the former z-score method, but the same three genes can obtain 98.08% prediction accuracy with the latter 0–1 normalization. The prediction accuracies of PAM and ClaNC methods are obviously improved on the cross-platform prostate dataset normalized with 0–1 normalization method, but the prediction accuracy becomes worse on the leukemia dataset similarly normalized. The results with 0–1 normalization also indicate that our method is still superior to PAM and ClaNC in prediction accuracy when the number of top-ranked genes is small enough.

**Figure 8:**
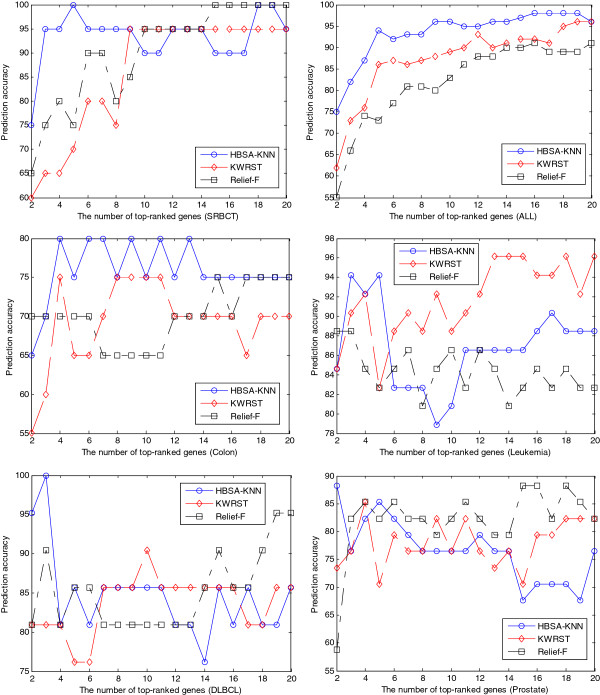
The comparisons of three gene ranking methods.

We further compare HBSA-KNN-based gene ranking method with the other two well-known gene ranking methods: Kruskal-Wallis rank sum test (KWRST) and Relief-F [71]. The results in Figure 8 show that our method consistently outperforms KWRST and Relief-F in prediction accuracy when the number of top-ranked genes is small enough. Although for the prostate dataset only top two genes obtain high prediction accuracy (88.24%) that is obviously greater than that of KWRST and Relief-F with the same number of genes, our method is still effective because this case still conforms to our goal that the most important tumor-related gene is ranked first. However, our method aims at finding as many more important tumor-related genes as possible, even though the important genes might include redundant ones from the viewpoint of classification. Thus the prediction accuracy might be worse as the number of top-ranked genes increases. For example, the prediction accuracy curves of leukemia and prostate in Figure 8 appear the situation. 

Moreover, better results can be achieved with more pre-selected genes by KWRST and with an acceptable search breadth increased in HBSA. For example, on the cross platform leukemia dataset, with the top 400 genes pre-selected by KWRST and the search breadth *w* of 450, the top eight genes selected by HBSA-KNN are {L09209, M23197, M11722, X95735, HG1612-HT1612, X62654, U77948, M31523} in which three genes L09209, HG1612-HT1612 and X62654 are not in the Leukemia 52 test set. Among these shared genes, the set of the top three genes {M23197, M11722, X95735} obtains 94.23% prediction accuracy on the independent test set, and the top five genes {M23197, M11722, X95735, U77948, M31523} and the top 84 genes can result in 96.15% and 98.08% prediction accuracies, respectively. More importantly, these important genes selected with this search breadth are shared with those genes shown in Additional file 1: Table S21. For the ALL dataset, the top eight genes selected by HBSA-KNN are {36985_at, 38242_at, 32207_at, 1287_at, 37470_at, 35974_at, 34168_at, 38518_at} in which only the rank orders of a few genes are changed compared with the same genes in Additional file 1: Table S18. The top seven, 20 and 25 genes can obtain 96%, 98% and 99% prediction accuracies, respectively, which are obviously improved compared with the corresponding results (shown in Tables 6 and 7) with less preselected genes and narrower search breadth. In conclusion, if the number of the initially selected genes and the search breadth are more appropriate, the prediction accuracy by HBSA will be further improved, which further proves that our method is indeed robust.

### Biological validation of the top-ranked genes

The association of top-ranked genes with tumor is analyzed in the context of individual gene function, pathway analysis, and protein-protein interaction (PPI) network to validate the effectiveness of the results. We first validate the top-ranked genes as tumor-related genes by known cancer gene list. Some unvalidated genes are validated by Cancer Linker Degree (CLD) analysis and relevant biomedical literature. Moreover, the selected genes are further validated by the fact that some pathways involving in the selected genes are closely related to tumor development. The following analysis is mainly based on the results obtained by HBSA-KNN.

#### Individual gene based literature validation

The top 50 genes selected by HSBA-SVM and HSBA-KNN are listed in Additional file 1: Tables S5-S10 and S17-S22, respectively. The known cancer genes were downloaded from the website (http://cbio.mskcc.org/cancergenes). 1086 known cancer genes are collected by querying the website for “oncogene”, “tumor suppressor” and “stability” [72]. The top 50 genes selected are analyzed through relevant biomedical literature. Here two case studies of the top-ranked genes on leukemia and prostate are presented as following. More analyses are available in the Additional file 1: Section 11.

Among the top 50 genes selected by HSBA-KNN on leukemia dataset, 10 genes (20%) are known cancer genes as listed in Additional file 1: Table S21. For other genes, by means of biomedical literature search and CLD calculation validation only on those among top 10 ones, we have successfully validated all the ten genes. The evidence of their involvement in cancer and the number or PubMed IDs of references documenting each gene-cancer association are shown in Table 8. CD33 (M23197) is expressed on the malignant blast cells in most cases of acute myeloid leukemia (AML) but not on normal hematopoietic pluripotent stem cells [73]. In vivo ablation of CD33+ cells achieves good results when treating patients with acute myeloid leukemia [74]. MARCKSL1, also named multidrug resistant associated protein (MRP), are found to be increasedly expressed in some vincristine-resistant cell lines [75]. SP3, a nuclear protein identified in numerous different biochemical assays at translocation break points, is associated with a subtype of acute myeloid leukemia [76]. CD63 (X62654) belongs to a newly defined family of genes for membrane proteins including CD33 which was recognized by monoclonal antibodies inhibitory to human T cell leukemia virus type 1-induced syncytium formation [77]. TCF3 (M31523) is involved in 19p13 chromosome rearrangement and acts as a tumor suppressor gene in B-cell precursor acute lymphoblastic leukemia [78]. CST3, also named cystatin C, was elevated in cancer patients than in controls. 

**Table 8:** Top 10 genes selected by HBSA-KNN from the leukemia dataset

**Top ten genes**	**CLD**	**Validation of tumor-related genes**
APLP2	4	stability
CD33	3	[73,74]
ZYX	--	Tumor suppressor
MARCKSL1		[75]
SP3	9	[76]
CD63	2	[77]; tumor suppressor
TCF3	--	[78]
PSME1	1	--
CCND3	--	Tumor suppressor
CST3	--	PMID: 17728092

The genes are sorted according to their frequency. If a gene is validated in the literature, the corresponding reference is shown (‘PMID’ denotes the PubMed ID).

For the prostate dataset, among the top 50 genes ranked by HSBA-KNN, 12 genes (24%) are known cancer genes (Additional file 1: Table S22). For other selected genes, we perform manual literature validation only on those among top 10 ones. We successfully validate nine of these ten genes (Table 9).

**Table 9:** Top 10 genes selected by HBSA-KNN on the prostate dataset

**Top ten genes**	**CLD**	**Validation of tumor-related genes**
MAF	7	Tumor oncogene
HPN	1	[79]
ABL1	46	Tumor suppressor
SLC25A6	--	[80]
CHD9	4	PMID: 20308527
SERPINB5	--	Tumor suppressor
A2R6W1	--	PMID:17259976;Tumor suppressor
WWC1	2	PMID: 16684779
NELL2	2	--
RBP1	4	PMID: 15280411

*The column is the same as described in Table*8*.*

A major 11-locus haplotype of SNPs in the HEPSIN gene (HPN), is significantly associated with prostate cancer, which supports that HPN (X07732) is a potentially important candidate gene involved in prostate cancer susceptibility [79]. SLC25A6, also named ANT3, is selectively required for TNF-α and oxidative stress-induced cell death in MCF-7 cells [80]. KIBRA is involved in estrogen receptor transactivation in breast cancer cells. Altered RBP1 expression and hypermethylation are common in prostate carcinoma. Both prostate adenocarcinoma and intraepithelial neoplasia show frequent RBP1 overexpression. CHD9 and NELL2 have CLD of four and two respectively as shown in the following network based analysis. The gene A2R6W1 was identified from Aspergillus niger and is hypothesized as a nucleus protein binding zinc ion and DNA for transcription regulation. Its relation with cancer deserves investigation.

#### Network-based analysis for top 10 genes

Aragues *et al.*[81] demonstrated that CLD of a protein, defined as the number of cancer genes to which it is connected, was a good indicator of the probability of being a cancer gene. We apply a protein-network-based method to analyze the neighborhood partners of the selected genes using all interactions in the Human Protein Reference Database (HPRD) [82]. The results are shown in Additional file 1: Figures S4-S8. For the leukemia dataset, among the 10 top-ranked genes, two genes (ZYX and CCND3) are cancer ones, five (APLP2, CD33, SP3, CD63, PSME1) and one other genes (CST3) are directly or indirectly associated with cancer genes, respectively. The CLDs of APLP2, CD33, SP3, CD63, PSME1, which are ranked first, second, fifth, seventh and ninth, respectively, are 4, 3, 9, 2 and 1, respectively. TTC3 and MARCKSL1 show no cancer gene linking, of which MARCKSL1 are increasedly expressed in some vincristine-resistant cell lines [75].

For the prostate dataset, our results show that three genes, namely MAF, ABL1 and SERPINB5, are cancer ones and most other top-ranked genes have a direct interaction with known cancer genes. The CLD of MAF, HPN, ABL1, CHD9, WWC1, NELL2 and RBP1 is 7, 1, 46, 4, 2, 2 and 4, respectively. We therefore infer that even if the remaining few genes not reported as cancer genes by previous studies are very possibly play a critical role in tumor genesis and cancer cell process as suggested by the fact that they interconnect directly with known cancer genes and ‘guilt of association’ rule.

#### Validation based on pathway analysis

The top-ranked genes are analyzed in the context of biological pathways on the website http://vortex.cs.wayne.edu/projects.htm. The pathways that the genes selected are most likely involved in are listed in Additional file 1: Tables S11–S16 and Tables S23–S28, where *p*-values are calculated by (14) and only ten pathways with the lowest *p*-values are selected. This approach is based on the assumption that the numbers of genes participating in different pathways conform to hypergeometric distribution. Given *N* genes in which *M* genes participate in a pathway *F*, we randomly select *K* genes which are considered to be significant. Then the *p-*value of having *x* or fewer genes in *F* can be calculated by summing the probabilities of a random list of *K* genes having 1, 2, ⋯, *x* genes of category *F*:

(13) $$p=\sum_{i=0}^{x}(\left(\begin{array}{c} M\\ i \end{array}\right)\left(\begin{array}{c} N-M\\ K-x \end{array}\right)/\left(\begin{array}{c} N\\ K \end{array}\right))$$

When *N* is very large, the hypergeometric distribution tends to be binominal. In this case, the *p*-value could also be calculated as:

(14) $$p=1-\sum_{i=0}^{x-1}\left(\begin{array}{c} K\\ i \end{array}\right){\left(\frac{M}{N}\right)}^{i}{\left(1-\frac{M}{N}\right)}^{K-i}$$

The top-ranked pathways in which the top 50 genes are involved include cell proliferation (such as cell cycle, DNA replication [83]), genomic stability (base excision repair, mismatch repair, etc), angiogenesis (like vascular endothelial growth factor(VEGF) signaling pathway),cancer metastasis (such as the pathway of cell adhesion molecules [84]),tumor suppressor pathway (such as p53 signaling pathway [85]), immunity escape (like pathways of antigen processing and presentation, B cell receptor signaling pathway, primary immunodeficiency, etc.) or progression of one specific or more than one kinds of cancers, etc.

Owing to a large number of top pathways involved, by means of biomedical literature we validate the tumor relevance of only four pathways supported by both HBSA-SVM and HBSA-KNN. For leukemia, B-cell antigen receptor (BCR) signal pathway is important for the survival of chronic lymphocytic leukemia cells which is regulated by overexpressed active protein kinase Cβ [86]. Heterogeneity in leukemia stem cell self-renewal potential supports the hypothesis that they derive from normal Hematopoietic stem cells [87]. Many transcription factors are either tumor suppressors or oncogenes, thus, mutations or aberrant regulation of them is associated with cancer [88]. DNA excision repair profiles of normal and leukemic human lymphocytes are different [89].

For the prostate dataset, Osman *et al.*[90] hypothesized that a pathway of prostate cancer progression involves p53 inactivation by mdm2 overexpression and that p21 transactivation via an alternative signaling system, rather than through a p53-dependent mechanism. Insulin signalling pathway is involved in the pathogenesis of various malignancies, increase cancer risk through its effect on cell proliferation, differentiation and apoptosis, and was reported to be involved in the tumorigenesis and neoplastic growth of the prostate [91]. The linkage of the morphological and functional changes of nucleolus and ribosome to cancer are reviewed in literature [92]. For cell cycle pathway, investigation has revealed that androgen acts as a master regulator of G1-S phase progression, able to induce signals that promote G1 cyclin-dependent kinase in prostate cancer cells [93].

We can conclude from the above signal pathway analysis that most of the pathways involving the selected genes are associated to the tumorigenesis, neoplastic growth or metastasis of tumor. From the analysis in the context of the molecular basis, the PPI networks and the pathways, we infer that the top-ranked genes are useful for cancer diagnosis as important potential biomarkers and may also provide insights into the mechanism of tumor genesis, development and metastasis.

## Discussions

To find optimal gene subsets from tremendous gene space, a challenge is how to avoid the effects of the curse of dimensionality. Usually, preliminary gene selection is often regarded as an indispensable step of classifier construction process. However, if it is performed only once on whole dataset and the performance is further evaluated by CV method, such gene selection may lead to an overoptimistic classification performance [24]. Even though the optimal gene subsets are selected independently on training set without the feedback of the test set and evaluated on independent test set, gene selection may also lead to over-fit the training set even test set if done improperly. On the other hand, over-fitting can also be easily caused by too many potential genes to discriminate among a small number of samples [68], which is evident by the fact that among the numerous gene subsets that can obtain 100% or nearly 100% *k*-fold CV accuracy on training set, only few can obtain very high prediction accuracy on independent test set. This is the reason why different methods usually find different optimal gene subsets and why many existing gene selection methods cannot consistently perform well on all tumor datasets. To address the over-fitting and selection bias problems, we adopt simple majority voting strategy to construct HBSA-based ensemble classifier with the optimal gene subsets. The results show that our ensemble classifier can efficiently avoid over-fitting and improve the stability of prediction performance.

Intuitively, the construction of classification model with more genes would obtain better generalization performance, but in fact the classifier constructed in such way usually leads to the bias of results. More importantly, we do not determine which genes contribute more to the classifier if a complicate classifier is used. As stated by Dabney [69], “a complicated classification model may be rejected for a simpler alternative even if the simpler alternative does not perform as well.” We observed that although simpler classification model constructed with fewer genes may be a little worse in accuracy than that with more genes, the results obtained by the simpler model result in less bias. We conclude that only a few top-ranked genes are enough for obtaining good classification performance. Particularly, when the number of the discriminate genes approximately equals to the number of subclasses in a dataset, high prediction accuracy is always obtained.

To prioritize genes for a specific tumor, the occurrence frequency of each gene in the selected gene subsets is counted and these genes are ranked according to the counted frequency to measure the importance of corresponding genes with respect to tumor. Our analysis based on protein-protein interaction network, individual gene function through relevant literatures and biological pathway demonstrate: 1) most of the top-ranked genes are important cancer genes or linked with cancer genes; 2) they are involved in cancer genesis, development, invasion, metastasis or angiogenesis. Thus these few top-ranked genes are useful for the screening of cancer genes and cancer biomarkers for tumor diagnosis, molecular treatment targets as a cancer-related gene pool and may also provide some insight into the mechanism of carcinogenesis and cancer development.

We also find that the occurrence frequency of a gene with respect to the number of those genes whose frequencies are greater than the corresponding frequency follows power-law distribution. As we know, power-law distribution is a universal phenomenon in nature. Gene regulatory network is widely accepted as a complex scare-free one with the property of power-law degree distribution. In such network, nodes represent genes and a link between two genes represents interaction between the two genes, and some nodes are more highly connected [94,95]. No doubt that the nodes with high degree play a very important role in network because structure always affects function. There may be no or weak interaction (minimum relevance) among the genes in the same optimal gene subset selected by HBSA, but the classification accuracy is the combined effect of the genes in an optimal gene subset. If we design a cooperation network in which nodes represent genes in an optimal gene subset and a link between each two genes in the gene subset represents cooperation between the two genes. There is no cooperation between two genes belonging to different optimal gene subsets. The node-degree distribution of the network constructed in such way by all gene subsets in A^*^ obviously follows power-law degree distribution. In such virtual network the genes with high node-degree correspond to the ones with high frequency in the optimal gene subsets, and these genes should closely involved in an actual gene regulatory network related to tumor.

However, the PPI network based analyses suggest that tumor-related genes are not always highly linked or hub ones in biological processes as indicated by the node linking degree. The node linking degree is the number of proteins that a node (protein) directly links and the nodes with higher degree are assumed as more important or hub proteins. For the prostate dataset, ABL1, a cytoplasmic and nuclear protein tyrosine kinase encoded by its proto-oncogene, ranked the third in Additional file 1: Table S22, is implicated in cell processes of cell differentiation, cell division, cell adhesion, and stress response, has a node linking degree of 100, with a CLD of 46. However, the protein MAF encoded by the first ranked gene MAF, another proto-oncogene, has a linking degree of only 12 degree in the PPI network, with a CLD of seven. For the SRBCT dataset, the fourth gene CAV1 in Additional file 1: Table S17, a tumor suppressor gene candidate that encodes protein Caveolin 1, has a linking degree of 73 in the PPI network, while the three proteins encoded by the top three genes CD99, MLLT11 and IGF2 in Additional file 1: Table S17 are 9, 0, and 16, respectively and have much lower CLD. One reason is that the protein-protein interactions in HPRD are most physical ones while our referred interactions of the selected genes are most functional. Since protein-protein interactions are highly dynamic in different cell states or highly different in different types of cells, the divergence may also be explained by the fact that the biological pathways in cancer cells may be greatly different or changed from pathways in normal cells, where many abnormal protein-protein interactions may be opened and normal interactions are closed. However, the real position of the cancer-related genes in the cancer oncogenesis and development pathways needs further study.

Our contribution in this paper is to propose two methods, namely the construction method of HBSA-based ensemble classifier and the HBSA-based gene ranking method, to obtain unbiased classification performance and find important tumor-related genes more biologically meaning in molecular tumor diagnosis. Unlike other search-based gene selection methods, such as GA/SVM [30] and sequential forward search (SFS) [36] that find only one optimal gene subsets, our HBSA can find as many optimal gene subsets as possible on training set and obtain determined results in each run. More importantly, these gene subsets by HBSA have the same minimum cardinal number which can ensure that it is reasonable to measure the significance of gene by using its occurrence frequency. Generally, HBSA-based gene ranking method is also different from many traditional gene ranking methods because our method simultaneously takes into account the discrimilability of individual gene and the relationship among multiple genes (the discrimilability of gene subset), while many traditional univariate Filters-based gene selection methods often select the top-ranked genes only according to their individual discriminative power and a few multivariate Filters-based methods only consider gene dependencies to improve classification performance. However, our method does not remove the redundant genes from the top-ranked genes because these redundant genes might be very important tumor-related genes [49]. On the other hand, our ensemble classifier is constructed by simplest but optimum individual classifiers on training set, which is different from other ensemble classifiers such as Bagging [96], Boosting [97] and random subspace method [98], in which individual classifiers are constructed by randomly resampling in sample set or feature set.

## Conclusions

Many machine learning and statistical algorithms for GEP-based tumor classification are available, but many of these methods might suffer from the problems of over-fitting and gene selection bias because the number of genes far exceeds the number of tumor tissue samples. Thus, we proposed two novel and robust methods (HBSA-based ensemble classification and HBSA-based gene ranking methods) to obtain high but unbiased prediction accuracy on independent test set and to find the most important tumor-related genes. HBSA-based ensemble classifier is constructed by using majority voting strategy on the basis of the selected optimum gene subsets selected by HBSA to improve the stability of the classification performance. HBSA-based gene ranking method is to prioritize the genes by using their occurrence frequencies counted in all of the selected gene subsets so that a set of significant genes can be found, which can be used as the biomarker of clinical tumor diagnosis and prognosis. Although HBSA implicates two problems: over-fitting and selection bias, both the proposed HBSA-based ensemble classifier and HBSA-based gene ranking method can successfully avoid the two problems. Moreover, the two methods are robust, stable and global optimum when such gene subsets selected are enough because the two methods are statistically established on the basis of the optimal gene subsets. Particularly, our methods not only are simple but also have rich biomedical interpretability. The experimental results indicate that our method can obtain high prediction accuracy with approximately minimum gene subset, and it overcomes the problem that too many genes can also lead to over-fitting phenomenon [68].

By comparing HBSA-SVM(Unbiased) and HBSA-KNN, we find that HBSA-KNN-based gene ranking method is slightly superior to HBSA-SVM-based one in gene selection. And the comparison of HBSA-SVM(Biased) and HBSA(Unbiased)demonstrates the bias degree of results. Most importantly, the analyses on the top-ranked genes in the context of individual gene function, pathway and PPI network biomedically justify our method. We also find that the occurrence frequency of gene in the optimal gene subsets with respect to the number of gene whose frequency is greater than the corresponding frequency follows power-law distribution, so we further infer that the important or hub genes related to tumor might be few. It may partly explain our finding that the number of informative genes that approximately equals to the number of subclasses in dataset is enough for obtaining good generalization performance. Lastly, we find that the genes with maximum differential expression among subclasses are not always the most important tumor-related genes, and some most important tumor-related genes are possibly those less differentially expressed ones.

Our future work will be mainly focused on utilizing the prior biomedical knowledge and exploring new heuristic search algorithms to reduce the time complexity of our current method. We are currently designing a novel time-saving method based on neighborhood rough set model to implement the same idea as this paper.

## Competing interests

The authors declare that they have no competing interests.

## Authors’ contributions

Shu-Lin Wang designed the HBSA algorithm and developed the computer programs in Matlab, performed the experiments, and drafted the manuscript. Xue-Ling Li and Jianwen Fang helped Shu-Lin Wang to analyze the numerical results with relevant biomedical literatures and revised the manuscript. All authors read and approved the final manuscript.

## Supplementary Material

Additional file 1 **Supplementary Tables and Figures [**[2]**,**[9]**-**[12]**,**[63]**-**[66]**,**[73]**,**[74]**,**[77]**-**[79]**,**[83]**-**[86]**,**[99]**-**[145]**]** Click here for file

## References

[B1] Hornberg JJ Bruggeman FJ Westerhoff HV Lankelmaa J Cancer: A systems biology disease Biosystems 2006 83 2–3 81 90 16426740 10.1016/j.biosystems.2005.05.014

[B2] Golub TR Slonim DK Tamayo P Huard C Gaasenbeek M Mesirov JP Coller H Loh ML Downing JR Caligiuri MA Molecular classification of cancer: Class discovery and class prediction by gene expression monitoring Science 1999 286 5439 531 537 10521349 10.1126/science.286.5439.531

[B3] Huang DS Zheng CH Independent component analysis-based penalized discriminant method for tumor classification using gene expression data Bioinformatics 2006 22 15 1855 1862 16709589 10.1093/bioinformatics/btl190

[B4] Zheng CH Huang DS Zhang L Kong XZ Tumor clustering using nonnegative matrix factorization with gene selection IEEE Trans Inf Technol Biomed 2009 13 4 599 607 19369170 10.1109/TITB.2009.2018115

[B5] Zheng CH Zhang L Ng VT Shiu SC Huang DS Molecular pattern discovery based on penalized matrix decomposition IEEE/ACM Trans Comput Biol Bioinform 2011 8 6 1592 1603 21519114 10.1109/TCBB.2011.79

[B6] Zheng CH Zhang L Ng TY Shiu SC Huang DS Metasample-based sparse representation for tumor classification IEEE/ACM Trans Comput Biol Bioinform 2011 8 5 1273 1282 21282864 10.1109/TCBB.2011.20

[B7] Wang SL Zhu YH Jia W Huang DS Robust classification method of tumor subtype by using correlation filters IEEE/ACM Trans Comput Biol Bioinform 2012 9 2 580 591 10.1109/TCBB.2011.135 22025761

[B8] Wang SL Li XL Zhang SW Gui J Huang DS Tumor classification by combining PNN classifier ensemble with neighborhood rough set based gene reduction Comput Biol Med 2010 40 2 179 189 20044083 10.1016/j.compbiomed.2009.11.014

[B9] Alon U Barkai N Notterman DA Gish K Ybarra S Mack D Levine AJ Broad patterns of gene expression revealed by clustering analysis of tumor and normal colon tissues probed by oligonucleotide arrays Proc Natl Acad Sci U S A 1999 96 12 6745 6750 10359783 10.1073/pnas.96.12.6745 PMC21986

[B10] Khan J Wei JS Ringner M Saal LH Ladanyi M Westermann F Berthold F Schwab M Antonescu CR Peterson C Classification and diagnostic prediction of cancers using gene expression profiling and artificial neural networks Nat Med 2001 7 6 673 679 11385503 10.1038/89044 PMC1282521

[B11] Shipp MA Ross KN Tamayo P Weng AP Kutok JL Aguiar RCT Gaasenbeek M Angelo M Reich M Pinkus GS Diffuse large B-cell lymphoma outcome prediction by gene-expression profiling and supervised machine learning Nat Med 2002 8 1 68 74 11786909 10.1038/nm0102-68

[B12] Singh D Febbo PG Ross K Jackson DG Manola J Ladd C Tamayo P Renshaw AA D’Amico AV Richie JP Gene expression correlates of clinical prostate cancer behavior Cancer Cell 2002 1 2 203 209 12086878 10.1016/s1535-6108(02)00030-2

[B13] Yeang CH Ramaswamy S Tamayo P Mukherjee S Rifkin RM Angelo M Reich M Lander E Mesirov J Golub T Molecular classification of multiple tumor types Bioinformatics 2001 17 Suppl 1 S316 S322 11473023 10.1093/bioinformatics/17.suppl_1.s316

[B14] Guyon I Weston J Vapnik V Gene selection for cancer classification using support vector machine Mach Learn 2002 46 1–3 389 422

[B15] Furey TS Cristianini N Duffy N Bednarski DW Schummer M Haussler D Support vector machine classification and validation of cancer tissue samples using microarray expression data Bioinformatics 2000 16 10 906 914 11120680 10.1093/bioinformatics/16.10.906

[B16] Xu Y Selaru FM Yin J Zou TT Shustova V Mori Y Sato F Liu TC Olaru A Wang S Artificial neural networks and gene filtering distinguish between global gene expression profiles of Barrett’s esophagus and esophageal cancer Cancer Res 2002 62 12 3493 3497 12067993

[B17] Ringner M Peterson C Microarray-based cancer diagnosis with artificial neural networks Biotechniques 2003 34 S30 S35 12664682

[B18] Sun GM Dong XY Xu GD Tumor tissue identification based on gene expression data using DWT feature extraction and PNN classifier Neurocomputing 2006 69 4–6 387 402

[B19] Huang DS Ip HHS Law KCK Chi Z Zeroing polynomials using modified constrained neural network approach Ieee T Neural Networ 2005 16 3 721 732 10.1109/TNN.2005.844912 15940999

[B20] Huang DS A constructive approach for finding arbitrary roots of polynomials by neural networks Ieee T Neural Networ 2004 15 2 477 491 10.1109/TNN.2004.824424 15384540

[B21] Li LP Darden TA Weinberg CR Levine AJ Pedersen LG Gene assessment and sample classification for gene expression data using a genetic algorithm/k-nearest neighbor method Comb Chem High Throughput Screen 2001 4 8 727 739 11894805 10.2174/1386207013330733

[B22] Tibshirani R Hastie T Narasimhan B Chu G Diagnosis of multiple cancer types by shrunken centroids of gene expression Proc Natl Acad Sci U S A 2002 99 10 6567 6572 12011421 10.1073/pnas.082099299 PMC124443

[B23] Tan YX Shi LM Tong WD Wang C Multi-class cancer classification by total principal component regression (TPCR) using microarray gene expression data Nucleic Acids Res 2005 33 1 56 65 15640445 10.1093/nar/gki144 PMC546133

[B24] Boulesteix AL WilcoxCV: an R package for fast variable selection in cross-validation Bioinformatics 2007 23 13 1702 1704 17495999 10.1093/bioinformatics/btm162

[B25] Saeys Y Inza I Larranaga P A review of feature selection techniques in bioinformatics Bioinformatics 2007 23 19 2507 2517 17720704 10.1093/bioinformatics/btm344

[B26] Kohavi R John GH Wrappers for feature subset selection Artif Intell 1997 97 1–2 273 324

[B27] Yan XT Deng MH Fung WK Qian MP Detecting differentially expressed genes by relative entropy J Theor Biol 2005 234 3 395 402 15784273 10.1016/j.jtbi.2004.11.039

[B28] Li T Zhang CL Ogihara M A comparative study of feature selection and multiclass classification methods for tissue classification based on gene expression Bioinformatics 2004 20 15 2429 2437 15087314 10.1093/bioinformatics/bth267

[B29] Peng HC Ding C Long FH Minimum redundancy - Maximum relevance feature selection IEEE Intell Syst 2005 20 6 70 71

[B30] Liu JJ Cutler G Li WX Pan Z Peng SH Hoey T Chen LB Ling XFB Multiclass cancer classification and biomarker discovery using GA-based algorithms Bioinformatics 2005 21 11 2691 2697 15814557 10.1093/bioinformatics/bti419

[B31] Inza I Larranaga P Blanco R Cerrolaza AJ Filter versus wrapper gene selection approaches in DNA microarray domains Artif Intell Med 2004 31 2 91 103 15219288 10.1016/j.artmed.2004.01.007

[B32] Davies S Russell S NP-completeness of searches for smallest possible feature sets Proceedings of the 1994 AAAI Fall Symposium on Relevance 1994 AAAI Press, New Orleans, LA, USA 37 39

[B33] Burke HB Discovering patterns in microarray data Mol Diagn 2000 5 4 349 357 11172499 10.1007/BF03262096

[B34] Zhu ZX Ong YS Dash M Markov blanket-embedded genetic algorithm for gene selection Pattern Recognition 2007 40 11 3236 3248

[B35] Wang YH Makedon FS Ford JC Pearlman J HykGene: a hybrid approach for selecting marker genes for phenotype classification using microarray gene expression data Bioinformatics 2005 21 8 1530 1537 15585531 10.1093/bioinformatics/bti192

[B36] Xiong MM Fang XZ Zhao JY Biomarker identification by feature wrappers Genome Res 2001 11 11 1878 1887 11691853 10.1101/gr.190001 PMC311150

[B37] Zhou X Tuck DP MSVM-RFE: extensions of SVM-RFE for multiclass gene selection on DNA microarray data Bioinformatics 2007 23 9 1106 1114 17494773 10.1093/bioinformatics/btm036

[B38] Reunanen J Overfitting in making comparisons between variable selection methods J Mach Learn Res 2003 3 7–8 1371 1382

[B39] Haibe-Kains B Desmedt C Sotiriou C Bontempi G A comparative study of survival models for breast cancer prognostication based on microarray data: does a single gene beat them all? Bioinformatics 2008 24 19 2200 2208 18635567 10.1093/bioinformatics/btn374 PMC2553442

[B40] Roth FP Bringing out the best features of expression data Genome Res 2001 11 11 1801 1802 11691842 10.1101/gr.215501

[B41] Huang HL Lee CC Ho SY Selecting a minimal number of relevant genes from microarray data to design accurate tissue classifiers Biosystems 2007 90 1 78 86 17291683 10.1016/j.biosystems.2006.07.002

[B42] Ransohoff DF Opinion - Rules of evidence for cancer molecular-marker discovery and validation Nat Rev Cancer 2004 4 4 309 314 15057290 10.1038/nrc1322

[B43] Huang DS Radial basis probabilistic neural networks: Model and application International Journal of Pattern Recognition and Artificial Intelligence 1999 13 7 1083 1101

[B44] Huang DS Du JX A Constructive Hybrid Structure Optimization Methodology for Radial Basis Probabilistic Neural Networks Ieee T Neural Networ 2008 19 12 2099 2115 10.1109/TNN.2008.2004370 19054734

[B45] Ambroise C McLachlan GJ Selection bias in gene extraction on the basis of microarray gene-expression data Proc Natl Acad Sci U S A 2002 99 10 6562 6566 11983868 10.1073/pnas.102102699 PMC124442

[B46] Wang LP Chu F Xie W Accurate cancer classification using expressions of very few genes IEEE/ACM Trans Comput Biol Bioinform 2007 4 1 40 53 17277412 10.1109/TCBB.2007.1006

[B47] Dudoit S Fridlyand J Speed TP Comparison of discrimination methods for the classification of tumors using gene expression data J Am Stat Assoc 2002 97 457 77 87

[B48] Wang SL Wang J Chen HW Li ST Zhang BY Heuristic breadth-first search algorithm for informative gene selection based on gene expression profiles Chinese Journal of Computers 2008 31 4 636 649

[B49] Li X Rao SQ Wang YD Gong BS Gene mining: a novel and powerful ensemble decision approach to hunting for disease genes using microarray expression profiling Nucleic Acids Res 2004 32 9 2685 2694 15148356 10.1093/nar/gkh563 PMC419591

[B50] Sotiriou C Neo SY McShane LM Korn EL Long PM Jazaeri A Martiat P Fox SB Harris AL Liu ET Breast cancer classification and prognosis based on gene expression profiles from a population-based study Proc Natl Acad Sci U S A 2003 100 18 10393 10398 12917485 10.1073/pnas.1732912100 PMC193572

[B51] Jain AK Duin RPW Mao JC Statistical pattern recognition: A review IEEE Trans Pattern Anal Mach Intell 2000 22 1 4 37

[B52] Asyali MH Colak D Demirkaya O Inan MS Gene expression profile classification: A review Curr Bioinforma 2006 1 1 55 73

[B53] Deng L Ma JW Pei J Rank sum method for related gene selection and its application to tumor diagnosis Chin Sci Bull 2004 49 15 1652 1657

[B54] Lee JW Lee JB Park M Song SH An extensive comparison of recent classification tools applied to microarray data Computational Statistics & Data Analysis 2005 48 4 869 885

[B55] Valente JMS Alves R Beam search algorithms for the early/tardy scheduling problem with release dates J Manuf Syst 2005 24 1 35 46

[B56] Vapnik VN Statistical learning theory 1998 Wiley Interscience, New York

[B57] Chang CC Lin CJ LIBSVM: a library for support vector machines Software available at http://wwwcsientuedutw/~cjlin/libsvm 2001.

[B58] Keerthi SS Lin CJ Asymptotic behaviors of support vector machines with Gaussian kernel Neural Comput 2003 15 7 1667 1689 12816571 10.1162/089976603321891855

[B59] Hsu CW Chang CC Lin CJ A practical guide to support vector classification Technical report, Department of Computer Science, National Taiwan University (http://wwwcsientuedutw/~cjlin/papershtml) 2003.

[B60] Evgeniou T Pontil M Elisseeff A Leave-one-out-error, stability, and generalization of voting combination of classifiers Mach Learn 2004 55 71 97

[B61] Breiman L Spector P Submodel selection and evaluation regression - the X-random case Int Stat Rev 1992 60 3 291 319

[B62] Sonego P Kocsor A Pongor S ROC analysis: applications to the classification of biological sequences and 3D structures Brief Bioinform 2008 9 3 198 209 18192302 10.1093/bib/bbm064

[B63] Yeoh EJ Ross ME Shurtleff SA Williams WK Patel D Mahfouz R Behm FG Raimondi SC Relling MV Patel A Classification, subtype discovery, and prediction of outcome in pediatric acute lymphoblastic leukemia by gene expression profiling Cancer Cell 2002 1 2 133 143 12086872 10.1016/s1535-6108(02)00032-6

[B64] Armstrong SA Staunton JE Silverman LB Pieters R de Boer ML Minden MD Sallan SE Lander ES Golub TR Korsmeyer SJ MLL translocations specify a distinct gene expression profile that distinguishes a unique leukemia Nat Genet 2002 30 1 41 47 11731795 10.1038/ng765

[B65] Stolovitzky GA Gene selection strategies in microarray expression data: applications to case–control studies Complex Systems Science in Biomedicine 2006 4 679 699

[B66] Welsh JB Sapinoso LM Su AI Kern SG Wang-Rodriguez J Moskaluk CA Frierson HF Hampton GM Analysis of gene expression identifies candidate markers and pharmacological targets in prostate cancer Cancer Res 2001 61 16 5974 5978 11507037

[B67] Liu CC Chen WSE Lin CC Liu HC Chen HY Yang PC Chang PC Chen JJW Topology-based cancer classification and related pathway mining using microarray data Nucleic Acids Res 2006 34 14 4069 4080 16914437 10.1093/nar/gkl583 PMC1557825

[B68] Ransohoff DF Rules of evidence for cancer molecular-marker discovery and validation Nat Rev Cancer 2004 4 4 309 314 15057290 10.1038/nrc1322

[B69] Dabney AR Classification of microarrays to nearest centroids Bioinformatics 2005 21 22 4148 4154 16174683 10.1093/bioinformatics/bti681

[B70] Dabney AR Storey JD Optimality driven nearest centroid classification from genomic data PLoS One 2007 2 10 e1002 17912341 10.1371/journal.pone.0001002 PMC1991588

[B71] Kononenko I Estimating attributes: Analysis and extensions of Relief European Conference on Machine Learning 1994 Springer, Catana, Italy 171 182

[B72] Higgins ME Claremont M Major JE Sander C Lash AE CancerGenes: a gene selection resource for cancer genome projects Nucleic Acids Res 2007 35 D721 D726 17088289 10.1093/nar/gkl811 PMC1781153

[B73] Linenberger ML CD33-directed therapy with gemtuzumab ozogamicin in acute myeloid leukemia: progress in understanding cytotoxicity and potential mechanisms of drug resistance Leukemia 2005 19 2 176 182 15592433 10.1038/sj.leu.2403598

[B74] Bernstein ID CD33 as a target for selective ablation of acute myeloid leukemia Clin Lymphoma 2002 2 S9 S11 11970770 10.3816/clm.2002.s.002

[B75] Hirose M The Process Behind the Expression of mdr-1/P-gp and mrp/MRP in Human Leukemia/Lymphoma Anticancer Res 2009 29 4 1073 1077 19414348

[B76] Wen CH Levitan D Li XJ Greenwald I spr-2, a suppressor of the egg-laying defect caused by loss of sel-12 presenilin in Caenorhabditis elegans, is a member of the SET protein subfamily Proc Natl Acad Sci U S A 2000 97 26 14524 14529 11114162 10.1073/pnas.011446498 PMC18952

[B77] Imai T Fukudome K Takagi S Nagira M Furuse M Fukuhara N Nishimura M Hinuma Y Yoshie O C33 antigen recognized by monoclonal antibodies inhibitory to human T cell leukemia virus type 1-induced syncytium formation is a member of a new family of transmembrane proteins including CD9, CD37, CD53, and CD63 J Immunol 1992 149 9 2879 2886 1401919

[B78] Barber KE Harrison CJ Broadfield ZJ Stewart ARM Wright SL Martineau M Strefford JC Moorman AV Molecular cytogenetic characterization of TCF3 (E2A)/19p 13.3 rearrangements in B-cell precursor acute lymphoblastic leukemia Genes Chromosomes Cancer 2007 46 5 478 486 17311319 10.1002/gcc.20431

[B79] Pal P Xi H Kaushal R Sun G Jin CH Jin L Suarez BK Catalona WJ Deka R Variants in the HEPSIN gene are associated with prostate cancer in men of European origin Hum Genet 2006 120 2 187 192 16783571 10.1007/s00439-006-0204-3

[B80] Yang ZQ Cheng W Hong LX Chen WZ Wang YH Lin SC Han JH Zhou HM Gu J Adenine nucleotide (ADP/ATP) translocase 3 participates in the tumor necrosis factor-induced apoptosis of MCF-7 cells Mol Biol Cell 2007 18 4681 4689 17855512 10.1091/mbc.E06-12-1161 PMC2043556

[B81] Aragues R Sander C Oliva B Predicting cancer involvement of genes from heterogeneous data BMC Bioinformatics 2008 9 172 189 18371197 10.1186/1471-2105-9-172 PMC2330045

[B82] Peri S Navarro JD Kristiansen TZ Amanchy R Surendranath V Muthusamy B Gandhi TKB Chandrika KN Deshpande N Suresh S Human protein reference database as a discovery resource for proteomics Nucleic Acids Res 2004 32 D497 D501 14681466 10.1093/nar/gkh070 PMC308804

[B83] Leibeling D Laspe P Emmert S Nucleotide excision repair and cancer J Mol Histol 2006 37 5–7 225 238 16855787 10.1007/s10735-006-9041-x

[B84] Behrens J The role of cell adhesion molecules in cancer invasion and metastasis Breast Cancer Res Treat 1993 24 175 184 8435473 10.1007/BF01833258

[B85] Sherr CJ McCormick F The RB and p53 pathways in cancer Cancer Cell 2002 2 2 103 112 12204530 10.1016/s1535-6108(02)00102-2

[B86] Abrams ST Lakum T Lin K Jones GM Treweeke AT Farahani M Hughes M Zuzel M Slupsky JR B-cell receptor signaling in chronic lymphocytic leukemia cells is regulated by overexpressed active protein kinase C beta II Blood 2007 109 3 1193 1201 17003377 10.1182/blood-2006-03-012021

[B87] Hope KJ Jin LQ Dick JE Acute myeloid leukemia originates from a hierarchy of leukemic stem cell classes that differ in self-renewal capacity Nat Immunol 2004 5 7 738 743 15170211 10.1038/ni1080

[B88] Libermann TA Zerbini LF Targeting transcription factors for cancer gene therapy Curr Gene Ther 2006 6 1 17 33 16475943 10.2174/156652306775515501

[B89] Buschfort C Muller MR Seeber S Rajewsky MF Thomale J DNA excision repair profiles of normal and leukemic human lymphocytes: Functional analysis at the single-cell level Cancer Res 1997 57 4 651 658 9044842

[B90] Osman I Drobnjak M Fazzari M Ferrara J Scher HI Cordon-Cardo C Inactivation of the p53 pathway in prostate cancer: Impact on tumor progression Clin Cancer Res 1999 5 8 2082 2088 10473090

[B91] Nandeesha H Insulin: a novel agent in the pathogenesis of prostate cancer Int Urol Nephrol 2009 41 2 267 272 18665451 10.1007/s11255-008-9440-x

[B92] Montanaro L Trere D Derenzini M Nucleolus, ribosomes, and cancer Am J Pathol 2008 173 2 301 310 18583314 10.2353/ajpath.2008.070752 PMC2475768

[B93] Balk SP Knudsen KE AR, the cell cycle, and prostate cancer Nucl Recept Signal 2008 6 e001 18301781 10.1621/nrs.06001 PMC2254330

[B94] Strogatz SH Exploring complex networks Nature 2001 410 6825 268 276 11258382 10.1038/35065725

[B95] Wang B Chen P Huang DS Li JJ Lok TM Lyu MR Predicting protein interaction sites from residue spatial sequence profile and evolution rate FEBS Lett 2006 580 2 380 384 16376878 10.1016/j.febslet.2005.11.081

[B96] Breiman L Bagging predictors Mach Learn 1996 24 2 123 140

[B97] Quinlan JR Bagging, boosting, and C4.5 Proceedings of the Thirteenth National Conference on Artificial Intelligence and the Eighth Innovative Applications of Artificial Intelligence Conference, Vols 1 and 2 1996 725 730

[B98] Ho TK The random subspace method for constructing decision forests IEEE Trans Pattern Anal Mach Intell 1998 20 8 832 844

[B99] Housa D Housova J Vernerova Z Haluzik M Adipocytokines and cancer Physiol Res 2006 55 3 233 244 16238454 10.33549/physiolres.930848

[B100] Wadman I Li JX Bash RO Forster A Osada H Rabbitts TH Baer R Specific in-vivo association between the bHLH and LIM proteins implicated in human T cell Leukemia EMBO J 1994 13 20 4831 4839 7957052 10.1002/j.1460-2075.1994.tb06809.x PMC395422

[B101] Macalma T Otte J Hensler ME Bockholt SM Louis HA KalffSuske M Grzeschik KH vonder Ahe D Beckerle MC Molecular characterization of human zyxin J Biol Chem 1996 271 49 31470 31478 8940160 10.1074/jbc.271.49.31470

[B102] Shi J Kahle A Hershey JWB Honchak BM Warneke JA Leong SPL Nelson MA Decreased expression of eukaryotic initiation factor 3f deregulates translation and apoptosis in tumor cells Oncogene 2006 25 35 4923 4936 16532022 10.1038/sj.onc.1209495

[B103] Rassenti LZ Huynh L Toy TL Chen L Keating MJ Gribben JG Neuberg DS Flinn IW Rai KR Byrd JC ZAP-70 compared with immunoglobulin heavy-chain gene mutation status as a predictor of disease progression in chronic lymphocytic leukemia N Engl J Med 2004 351 9 893 901 15329427 10.1056/NEJMoa040857

[B104] Vinante F Rigo A Vincenzi C Ricetti MM Marrocchella R Chilosi M Cassatella MA Bonazzi L Pizzolo G IL-8 messenger-RNA expression and IL-8 production by acute myeloid-leukemia cells Leukemia 1993 7 10 1552 1556 8412317

[B105] Amin S Parker A Mann J ZAP70 in chronic lymphocytic leukemia Int J Biochem Cell Biol 2008 40 9 1654 1658 17625948 10.1016/j.biocel.2007.05.016

[B106] Lepont P Stickney JT Foster LA Meng JJ Hennigan RF Ip W Point mutation in the NF2 gene of HEI-193 human schwannoma cells results in the expression of a merlin isoform with attenuated growth suppressive activity Mutat Res Fundam Mol Mech Mutagen 2008 637 1–2 142 151 10.1016/j.mrfmmm.2007.07.015 PMC2233940 17868749

[B107] Hulit J Bash T Fu MF Galbiati F Albanese C Sage DR Schlegel A Zhurinsky J Shtutman M Ben-Ze’ev A et al: The cyclin D1 gene is transcriptionally repressed by caveolin-1 J Biol Chem 2000 275 28 21203 21209 10747899 10.1074/jbc.M000321200

[B108] Tirado OM Mateo-Lozano S Villar J Dettin LE Llort A Gallego S Ban J Kovar H Notario V Caveolin-1 (CAV1) is a target of EWS/FLI-1 and a key determinant of the oncogenic phenotype and tumorigenicity of Ewing’s sarcoma cells Cancer Res 2006 66 20 9937 9947 17047056 10.1158/0008-5472.CAN-06-0927

[B109] Meyer A van Golen CM Boyanapalli M Kim B Soules ME Feldman EL Integrin-linked kinase complexes with caveolin-1 in human neuroblastoma cells Biochemistry 2005 44 3 932 938 15654749 10.1021/bi048619r

[B110] Ramani P Rampling D Link M Immunocytochemical study of 12E7 in small round-cell tumors of childhood - an assessment of its sensitivity and specificity Histopathology 1993 23 6 557 561 8314240 10.1111/j.1365-2559.1993.tb01243.x

[B111] Lin HJ Shaffer KM Sun ZR Jay G He WW Ma W AF1q, a differentially expressed gene during neuronal differentiation, transforms HEK cells into neuron-like cells Mol Brain Res 2004 131 1–2 126 130 15530661 10.1016/j.molbrainres.2004.07.022

[B112] Weir ML Muschler J Dystroglycan: Emerging roles in mammary gland function J Mammary Gland Biol Neoplasia 2003 8 4 409 419 14985637 10.1023/B:JOMG.0000017428.38034.a7

[B113] Poggi A Catellani S Bruzzone A Caligaris-Cappio F Gobbi M Zocchi MR Lack of the leukocyte-associated Ig-like receptor-1 expression in high-risk chronic lymphocytic leukaemia results in the absence of a negative signal regulating kinase activation and cell division Leukemia 2008 22 5 980 988 18288129 10.1038/leu.2008.21

[B114] Harnacke K Kruhoffer M Orntoft TF Hass R Down-modulation of poly(ADP-ribose) polymerase-1 (PARP-1) in human TUR leukemia cells restores transcriptional responsiveness for differentiation and cell cycle arrest Eur J Cell Biol 2005 84 11 885 896 16323285 10.1016/j.ejcb.2005.08.009

[B115] Kees UR Ford J Watson M Murch A Ringner M Walker RL Meltzer P Gene expression profiles in a panel of childhood leukemia cell lines mirror critical features of the disease Mol Cancer Ther 2003 2 7 671 677 12883040

[B116] Pottier N Cheok MH Yang W Assem M Tracey L Obenauer JC Panetta JC Relling MV Evans WE Expression of SMARCB1 modulates steroid sensitivity in human lymphoblastoid cells: identification of a promoter snp that alters PARP1 binding and SMARCB1 expression Hum Mol Genet 2007 16 2261 2271 17616514 10.1093/hmg/ddm178

[B117] Nakayama J Yamamoto M Hayashi K Satoh H Bundo K Kubo M Goitsuka R Farrar MA Kitamura D BLNK suppresses pre-B-cell leukemogenesis through inhibition of JAK3 Blood 2009 113 7 1483 1492 19047679 10.1182/blood-2008-07-166355 PMC2644075

[B118] Mizukami Y Jo WS Duerr EM Gala M Li JN Zhang XB Zimmer MA Iliopoulos O Zukerberg LR Kohgo Y Induction of interleukin-8 preserves the angiogenic response in HIF-1 alpha-deficient colon cancer cells Nat Med 2005 11 9 992 997 16127434 10.1038/nm1294

[B119] Cacev T Radosevic S Krizanac S Kapitanovic S Influence of interleukin-8 and interleukin-10 on sporadic colon cancer development and progression Carcinogenesis 2008 29 8 1572 1580 18628251 10.1093/carcin/bgn164

[B120] Barshishat M Ariel A Cahalon L Chowers Y Lider O Schwartz B TNF alpha and IL-8 regulate the expression and function of CD44 variant proteins in human colon carcinoma cells Clin Exp Metastasis 2002 19 4 327 337 12090473 10.1023/a:1015528314970

[B121] Hellmuth M Wetzler C Nold M Chang JH Frank S Pfeilschifter J Muhl H Expression of interleukin-8, heme oxygenase-1 and vascular endothelial growth factor in DLD-1 colon carcinoma cells exposed to pyrrolidine dithiocarbamate Carcinogenesis 2002 23 8 1273 1279 12151344 10.1093/carcin/23.8.1273

[B122] Vavricka SR Musch MW Chang JE Nakagawa Y Phanvijhitsiri K Waypa TS Merlin D Schneewind O Chang EB hPepT1 transports muramyl dipeptide, activating NF-kappa B and stimulating IL-8 secretion in human colonic Caco2/bbe cells Gastroenterology 2004 127 5 1401 1409 15521010 10.1053/j.gastro.2004.07.024

[B123] delaCadena M Fernandez J deCarlos A MartinezZorzano V GilMartin E RodriguezBerrocal FJ Low levels of alpha-L-fucosidase activity in colorectal cancer are due to decreased amounts of the enzymatic protein and are related with Dukes' stage Int J Oncol 1996 9 4 747 754 21541579 10.3892/ijo.9.4.747

[B124] Kishino H Waddell PJ Correspondence analysis of genes and tissue types and finding genetic links from microarray data Genome Inform Ser Workshop Genome Inform 2000 11 83 95 11700590

[B125] Hill O Cetin Y Cieslak A Magert HJ Forssmann WG A new human guanylate cyclase-activating peptide (GCAP-II, uroguanylin): precursor cDNA and colonic expression Biochimica Et Biophysica Acta-Protein Structure and Molecular Enzymology 1995 1253 2 146 149 10.1016/0167-4838(95)00204-4 8519795

[B126] Li MH Lin YM Hasegawa S Shimokawa T Murata K Kameyama M Ishikawa O Katagiri T Tsunoda T Nakamura Y Genes associated with liver metastasis of colon cancer, identified by genome-wide cDNA microarray Int J Oncol 2004 24 2 305 312 14719106

[B127] Reubi JC In-vitro identification of vasoactive-intestinal-peptide receptors in human tumors: implications for tumor imaging J Nucl Med 1995 36 10 1846 1853 7562054

[B128] Giroux V Iovanna J Dagorn JC Probing the human kinome for kinases involved in pancreatic cancer cell survival and gemcitabine resistance FASEB J 2006 20 12 1982 1991 17012250 10.1096/fj.06-6239com

[B129] Zhou CZ Qiu GQ Wang XL Fan JW Tang HM Sun YH Wang Q Huang F Yan DW Li DW Screening of tumor suppressor genes on 1q31.1–32.1 in Chinese patients with sporadic colorectal cancer Chin Med J 2008 121 24 2479 2486 19187582

[B130] Hiraga J Katsumi A Iwasaki T Abe A Kiyoi H Matsushita T Kinoshita T Naoe T Prognostic analysis of aberrant somatic hypermutation of RhoH gene in diffuse large B cell lymphoma Leukemia 2007 21 8 1846 1847 17443219 10.1038/sj.leu.2404717

[B131] Lin KR Lee SF Hung CM Li CL Yang-Yen HF Yen JJY Survival factor withdrawal-induced apoptosis of TF-1 cells involves a TRB2-Mcl-1 axis-dependent pathway J Biol Chem 2007 282 30 21962 21972 17545167 10.1074/jbc.M701663200

[B132] Monti S Savage KJ Kutok JL Feuerhake F Kurtin P Mihm M Wu BY Pasqualucci L Neuberg D Aguiar RCT Molecular profiling of diffuse large B-cell lymphoma identifies robust subtypes including one characterized by host inflammatory response Blood 2005 105 5 1851 1861 15550490 10.1182/blood-2004-07-2947

[B133] Gez S Crossett B Christopherson RI Differentially expressed cytosolic proteins in human leukemia and lymphoma cell lines correlate with lineages and functions Biochimica Et Biophysica Acta-Proteins and Proteomics 2007 1774 1173 1183 10.1016/j.bbapap.2007.06.011 17698427

[B134] Lacayo NJ Meshinchi S Kinnunen P Yu R Wang Y Stuber CM Douglas L Wahab R Becton DL Weinstein H Gene expression profiles at diagnosis in de novo childhood AML patients identify FLT3 mutations with good clinical outcomes Blood 2004 104 9 2646 2654 15251987 10.1182/blood-2003-12-4449

[B135] Furusato B Gao CL Ravindranath L Chen YM Cullen J McLeod DG Dobi A Srivastava S Petrovics G Sesterhenn IA Mapping of TMPRSS2-ERG fusions in the context of multi-focal prostate cancer Mod Pathol 2008 21 2 67 75 18065961 10.1038/modpathol.3800981

[B136] Chen L Li XY Wang GI Wang Y Zhu YY Zhu JW Clinicopathological significance of overexpression of TSPAN1, K167 and CD34 in gastric carcinoma Tumori 2008 94 4 531 538 18822690 10.1177/030089160809400415

[B137] Saleem M Kweon MH Johnson JJ Adhami VM Elcheva I Khan N Bin Hafeez B, Bhat KMR, Sarfaraz S, Reagan-Shaw S et al: S100A4 accelerates tumorigenesis and invasion of human prostate cancer through the transcriptional regulation of matrix metalloproteinase 9 Proc Natl Acad Sci U S A 2006 103 40 14825 14830 16990429 10.1073/pnas.0606747103 PMC1595436

[B138] Ross ME Zhou X Song G Shurtleff SA Girtman K Williams WK Liu HC Mahfouz R Raimondi SC Lenny N Patel A Downing JR Classification of pediatric acute lymphoblastic leukemia by gene expression profiling Blood 2003 102 8 2951 2959 12730115 10.1182/blood-2003-01-0338

[B139] Yazawa S Nakamura J-Asao T Nagamachi Y Sagi M Malta KL Achikawa TT Akamatsu M Aberrant α1 → 2 fucosyltransferases found in human colorectal carcinoma involved in the accumulation of Leb and Y Antigens in Colorectal Tumors Cancer Sci 1993 84 9 989 995 10.1111/j.1349-7006.1993.tb00190.x PMC5919283 8407568

[B140] Schulz TJ Thierbach R Voigt A Drewes G Mietzner B Steinberg P Pfeiffer AFH Ristow M Induction of oxidative metabolism by mitochondrial frataxin inhibits cancer growth - Otto Warburg revisited J Biol Chem 2006 281 2 977 981 16263703 10.1074/jbc.M511064200

[B141] Lan LX Han HB Zuo HJ Chen ZG Du YT Zhao W Gu J Zhang ZQ Upregulation of myosin Va by Snail is involved in cancer cell migration and metastasis Int J Cancer 2010 126 1 53 64 19521958 10.1002/ijc.24641

[B142] van Spriel AB Puls KL Sofi M Pouniotis D Hochrein H Orinska Z Knobeloch KP Plebanski M Wright MD A regulatory role for CD37 in T cell proliferation J Immunol 2004 172 5 2953 2961 14978098 10.4049/jimmunol.172.5.2953

[B143] Lutsiak MEC Tagaya Y Adams AJ Schlom J Sabzevari H Tumor-Induced Impairment of TCR Signaling Results in Compromised Functionality of Tumor-Infiltrating Regulatory T Cells J Immunol 2008 180 9 5871 5881 18424706 10.4049/jimmunol.180.9.5871 PMC2636572

[B144] Polson AG Calemine-Fenaux J Chan P Chang W Christensen E Clark S de Sauvage FJ Eaton D Elkins K Elliott JM Antibody-Drug Conjugates for the Treatment of Non-Hodgkin’s Lymphoma: Target and Linker-Drug Selection Cancer Res 2009 69 6 2358 2364 19258515 10.1158/0008-5472.CAN-08-2250

[B145] Sakane-Ishikawa E Nakatsuka S-Tomita Y Fujita S Nakamichi I Takakuwa T Sugiyama H Fukuhara S Hino M Kanamaru A Prognostic Significance of BACH2 Expression in Diffuse Large B-Cell Lymphoma: A Study of the Osaka Lymphoma Study Group J Clin Oncol 2005 23 31 8012 8017 16258099 10.1200/JCO.2005.02.1626

