# Supplementary material for: Finding minimum gene subsets with heuristic breadth-first search algorithm for robust tumor classification
Source: BMC Bioinformatics. 2012 Jul 25;13:178. doi: 10.1186/1471-2105-13-178 (PMC3465202; doi:10.1186/1471-2105-13-178)
Supplement: Additional file 1 — Supplementary Tables and Figures [[2],[9]-[12],[63]-[66],[73],[74],[77]-[79],[83]-[86],[99]-[145]] [file 1471-2105-13-178-S1.docx]

Finding Minimum Gene Subsets with Heuristic Breadth-first Search Algorithm for Robust Tumor Classification

Shu-Lin Wang^1,2,3^, Xue-Ling Li^2^,and Jianwen Fang^3*^

^1^ College of Information Science and Engineering, Hunan University, Changsha, Hunan 410082, China

^2^Intelligent Computing Laboratory, Hefei Institute of Intelligent Machines, Chinese Academy of Sciences, Hefei, Anhui 230031, China

^3^Applied Bioinformatics Laboratory, the University of Kansas, 2034 Becker Drive,Lawrence, KS 66047, USA

*Corresponding author

Email addresses:

Shu-Lin Wang: jt_slwang@hotmail.com

Xue-Ling Li: xlli@iim.ac.cn

Jianwen Fang: jwfang@ku.edu

————————————————————

Table of Contents

[1 Training set and test set 2](#_Toc319693244)

[2 An examble of HBSA 3](#_Toc319693245)

[3 Top-ranked gene lists selected by HBSA-SVM 3](#_Toc319693246)

[4 Pathway analysis of the genes selected by HBSA-SVM 14](#_Toc319693247)

[5 Top-ranked genes selected by HBSA-KNN 16](#_Toc319693248)

[6 Pathway analysis of the genes selected by HBSA-KNN 27](#_Toc319693249)

[7 Comparison of classification accuracy for three experimental methods 29](#_Toc319693250)

[8 Comparison of experimental results with 0-1 normalization 30](#_Toc319693251)

[9 Partial results on the colon tumor dataset 31](#_Toc319693252)

[10 Functional analysis of the top-ranked genes selected by HBSA-SVM 32](#_Toc319693253)

[11 Network analysis of the top 10 genes selected by HBSA-KNN 36](#_Toc319693254)

[References 40](#_Toc319693255)

# 1 Training set and test set

In our experiments we apply our approach to ninepublicly available tumor datasets: Small Round Blue Cell Tumor (SRBCT)[10], Acute Lymphoblastic Leukemia (ALL) [63], Colon tumor [9], Leukemia72 [2], Leukemia52 [64], Diffuse Large B-cell Lymphomas (DLBCL77) [11], DLBCL21 (obtained in R. Dalla-Favera’s lab at Columbia University)[65], Prostate102 [12], and Prostate34 [66] datasets, in which three pairs of datasets are used as the evaluation of generalized performance for cross-platform classification model.

**Table S1** The descriptions of nine tumor datasets used in our study.

| NO. | Dataset | Authors | #samples | #Genes | #Subclasses |
| --- | --- | --- | --- | --- | --- |
| 1 | SRBCT | (Khan *et al.*, 2001) | 83 | 2,308 | 4 |
| 2 | ALL | (Yeoh *et al*., 2002) | 248 | 12625 | 6 |
| 3 | Colon tumor | (Alon *et al.*, 1999) | 62 | 2,000 | 2 |
| 4 | Leukemia72 | (Golub *et al.*, 1999) | 72 | 7,129 | 2 |
| 5 | Leukemia52 | (Armstrong *et al*., 2002) | 52 | 12582 | 2 |
| 6 | DLBCL77 | (Shipp *et al*., 2002) | 77 | 7129 | 2 |
| 7 | DLBCL21 | (R. Dalla-Favera’s lab) | 21 | 12581 | 2 |
| 8 | Prostate102 | (Singh *et al*., 2002) | 102 | 12600 | 2 |
| 9 | Prostate34 | (Welsh *et al*. 2001) | 34 | 12626 | 2 |

From theweb sitehttp://research.nhgri.nih.gov/microarray/Supplement, we downloaded the SRBCT dataset which contains 88 samples with 2,308 genes in each sample. According to the suggestion in the original literature, there are 63 training samples and 25 test samples which contain five non tumor-related samples as shown in TablesS2 and S3.The 63 training samples contain 23 Ewing family of tumors (EWS), 20 rhabdomyosarcoma (RMS), 12 neuroblastoma (NB), and eight Burkitt lymphomas (BL). The test samples contain six EWSs, five RMSs, six NBs, three BLs, and five non tumor-related samples. The five tumor-unrelated samples are removed in our experiments.For the colon tumor dataset, the first 42 samples in original dataset are used as training set, and the last 20 samples are used as test set.

**Table S2** The partition of training set and test set for tumor datasets.

| NO. | Dataset | Training set | Test set |
| --- | --- | --- | --- |
| 1 | SRBCT | 63 | 20 |
| 2 | ALL | 148 | 100 |
| 3 | Colon tumor | 42 | 20 |
| 4 | Leukemia | Leukemia72 | Leukemia52 |
| 5 | DLBCL | DLBCL77 | DLBCL21 |
| 6 | Prostate | Prostate102 | Prostate34 |

**Table S3.**Descriptions of the SRBCT dataset.

| Subclass | #Original Dataset | #Training set | #Test set |
| --- | --- | --- | --- |
| EWS | 29 | 23 | 6 |
| NB | 18 | 12 | 6 |
| RMS | 25 | 20 | 5 |
| BL | 11 | 8 | 3 |
| Non-SRBCT | 5 | 0 | 5 |
| Total | 88 | 63 | 25 |

The partition of training set and test set for ALL dataset is shown in Tables S2 and S4. For example, for subclass BCR-ABL, there are 15 samples. According to the rank of samples in original dataset, the first nine samples are used as training set, and the last six samples are used as test set. The others are deduced similarly.

**Table S4** The partition of training set and testing set for ALL dataset.

| NO. | Subclass | #Training set | #Test set |
| --- | --- | --- | --- |
| 1 | BCR-ABL | 9 | 6 |
| 2 | E2A-PBX1 | 16 | 11 |
| 3 | Hyperdip>50 | 39 | 25 |
| 4 | MLL | 12 | 8 |
| 5 | T-ALL | 25 | 18 |
| 6 | TEL-AML1 | 47 | 32 |
| Total |  | 148 | 100 |

# 2 An examble of HBSA

Assume we have a gene set $G^{*}=\{a, b, c,d\}$ with four genes selected by KWRST from a sample set and the search breadth is set to four. We firstly generate a root node assigned an empty set $\emptyset$,and then expand the root node to four child nodes assigned with four genes $\{a, b, c,d\}$, respectively. Then the four nodes in layer 1 are expanded to 12 child nodes in layer 2, and the classification accuracy of all nodes in layer 2 are measured by $Acc\left( T \right)$, respectively, where $T$ denotes the gene set constructed by all genes on the path from the root node to the present leaf node. For example, the $T$ of the node 6 is {a, b}, so the accuracy of the node 6 is assigned with $Acc(\{a,b\})$. Then the four nodes with the highest accuracy are selected to be expanded to eight child nodes. Note that there should be no the same gene on one path. Finally the accuracy of each node in layer 3 is measured by $Acc\left( T \right)$. Assume the nodes 19, 20, 22 and 23 in layer 3 can achieve the highest accuracy, and the four nodes will be selected to be expanded while other nodes in this layer are discarded. If in this layer there is at least one node whose accuracy is greater than or equal to the given accuracy threshold, the search process is ended. Thus, if the search process ends, the optimal gene subsets $A^{*}$ is {{a, d, b}, {a, d, c}, {b, c, a}, {c, b, d}}, whichincludes four optimal gene subsets.

$$\emptyset$$

a

b

c

b

c

a

c

a

b

1

2

3

4

6

7

8

Layer 0

Layer 1

d

d

d

9

10

5

a

b

d

c

Layer 2

11

12

13

14

15

16

b

a

a

18

19

c

d

20

a

b

d

21

22

23

24

25

Layer 3

17

**Fig. S1.**A diagram of search procedure using HBSA.

# 3 Top-ranked gene lists selected by HBSA-SVM

For six tumor datasets, Tables S5-S10 show the descriptions of 50 top-ranked genes selected by the HBSA-SVM method and ranked by their occurrence frequencies in descending order. We also downloaded known cancer genes from the website (http://cbio.mskcc.org/cancergenes) as of August 2009. 1086 known cancer genes are collected by querying the website for “oncogene”, “tumor suppressor” and “stability”. The 1086 known cancer genes comprise 338 oncogenes, 313 stability genes and 435 tumor suppressor genes. In Table S5-S10, column “Is cancer gene?” denotes whether the corresponding gene selected belongs to the known cancer genes.

**Table S5**Description of 50 top-ranked genes for SRBCT dataset

| No. | Probe No. | Gene symbol | Description | Frequency | Is cancer gene? |
| --- | --- | --- | --- | --- | --- |
| 1 | 769716 | NF2 | neurofibromin 2 (bilateral acoustic neuroma) | 188 | Y |
| 2 | 770394 | FCGRT | Fc fragment of IgG, receptor, transporter, alpha | 132 |  |
| 3 | 377461 | CAV1 | caveolin 1, caveolae protein, 22kD | 68 | Y |
| 4 | 1435862 | CD99 | antigen identified by monoclonal antibodies 12E7, F21 and O13 | 56 |  |
| 5 | 812105 | MLLT11 | transmembrane protein | 37 |  |
| 6 | 796258 | SGCA | sarcoglycan, alpha (50kD dystrophin-associated glycoprotein) | 31 | Y |
| 7 | 859359 | TP53I3 | quinone oxidoreductase homolog | 20 | Y |
| 8 | 782193 | LATS2 | Thioredoxin | 14 | Y |
| 9 | 784593 | RND3 | ESTs | 13 | Y |
| 10 | 814260 | FVT1 | follicular lymphoma variant translocation 1 | 13 |  |
| 11 | 308231 | MYO1B | Homo sapiens incomplete cDNA for a mutated allele of a myosin class I, myh-1c | 12 |  |
| 12 | 207274 | IGF2 | Human DNA for insulin-like growth factor II (IGF-2); exon 7 and additional ORF | 9 |  |
| 13 | 241412 | ELF1 | E74-like factor 1 (ets domain transcription factor) | 9 |  |
| 14 | 81518 | OCRL | apelin; peptide ligand for APJ receptor | 9 |  |
| 15 | 295985 | CDK6 | ESTs | 8 | Y |
| 16 | 563673 | ALDH7A1 | antiquitin 1 | 7 |  |
| 17 | 43733 | GYG2 | glycogenin 2 | 7 |  |
| 18 | 486110 | PFN2 | profilin 2 | 6 |  |
| 19 | 629896 | MAP1B | microtubule-associated protein 1B | 6 |  |
| 20 | 21652 | CTNNA1 | catenin (cadherin-associated protein), alpha 1 (102kD) | 6 |  |
| 21 | 236282 | WAS | Wiskott-Aldrich syndrome (ecezema-thrombocytopenia) | 6 |  |
| 22 | 841641 | CCND1 | cyclin D1 (PRAD1: parathyroid adenomatosis 1) | 6 | Y |
| 23 | 841620 | DPYSL2 | dihydropyrimidinase-like 2 | 6 |  |
| 24 | 221826 | GNA11 | guanine nucleotide binding protein (G protein), alpha 11 (Gq class) | 5 |  |
| 25 | 504791 | GSTA4 | glutathione S-transferase A4 | 5 |  |
| 26 | 82903 | TAP2 | TAP binding protein (tapasin) | 5 |  |
| 27 | 842918 | FARP1 | chondrocyte-derived ezrin-like protein | 5 |  |
| 28 | 784224 | FGFR4 | fibroblast growth factor receptor 4 | 5 |  |
| 29 | 143306 | Lsp1 | lymphocyte-specific protein 1 | 4 |  |
| 30 | 782503 | FADS1 | Homo sapiens clone 23716 mRNA sequence | 4 |  |
| 31 | 204545 | ANTXR1 | ESTs | 4 |  |
| 32 | 813742 | PTK7 (CCK4) | PTK7 protein tyrosine kinase 7 | 4 |  |
| 33 | 183337 | DMA | major histocompatibility complex, class II, DM alpha | 4 |  |
| 34 | 132848 |  | ESTs | 4 |  |
| 35 | 293859 |  | Putative prostate cancer tumor suppressor | 4 |  |
| 36 | 125092 | SLC26A10 | UDP-N-acetyl-alpha-D-galactosamine:(N-acetylneuraminyl)-galactosylglucosylceramide N-acetylgalactosaminyltransferase (GalNAc-T) | 4 |  |
| 37 | 782811 | HMGA1 | high-mobility group (nonhistone chromosomal) protein isoforms I and Y | 4 | Y |
| 38 | 897177 | PGAM1 (PGAMA) | phosphoglycerate mutase 1 (brain) | 4 |  |
| 39 | 134748 | GCSH | glycine cleavage system protein H (aminomethyl carrier) | 3 |  |
| 40 | 878652 | PCOLCE | postmeiotic segregation increased 2-like 12 | 3 |  |
| 41 | 383188 | RCVRN | Recoverin | 3 |  |
| 42 | 878280 | CRMP1 | collapsin response mediator protein 1 | 3 |  |
| 43 | 745343 | REG1A | regenerating islet-derived 1 alpha (pancreatic stone protein, pancreatic thread protein) | 3 |  |
| 44 | 212542 | PBX1 | Homo sapiens mRNA; cDNA DKFZp586J2118 (from clone DKFZp586J2118) | 3 |  |
| 45 | 624360 | PSMB8 | proteasome (prosome, macropain) subunit, beta type, 8 (large multifunctional protease 7) | 3 |  |
| 46 | 435953 | ITPR3 |  | 3 |  |
| 47 | 203003 | NME4 | non-metastatic cells 4, protein expressed in | 3 |  |
| 48 | 814526 | RBM38 | ESTs | 3 |  |
| 49 | 668442 | DDR2 | discoidin domain receptor family, member 2 | 3 |  |
| 50 | 767183 | HCLS1 | hematopoietic cell-specific Lyn substrate 1 | 3 |  |

**Table S6** Description of 50 top-ranked genes for ALL dataset

| No. | Probe No. | Gene symbol | Description | Frequency | Is cancer gene? |
| --- | --- | --- | --- | --- | --- |
| 1 | 36985_at | IDI1 | Cluster Incl. X17025:Human homolog of yeast IPP isomerase /cds=(50,736) /gb=X17025 /gi=488749 /ug=Hs.76038 /len=1807 | 299 |  |
| 2 | 32207_at | MPP1 | Cluster Incl. M64925:Human palmitoylated erythrocyte membrane protein (MPP1) mRNA, complete cds /cds=(103,1503) /gb=M64925 /gi=189785 /ug=Hs.1861 /len=1989 | 173 |  |
| 3 | 37470_at | LAIR1 | Cluster Incl. AF013249:Homo sapiens leukocyte-associated Ig-like receptor-1 (LAIR-1) mRNA, complete cds /cds=(68,931) /gb=AF013249 /gi=2352940 /ug=Hs.115808 /len=1675 | 159 |  |
| 4 | 1287_at | PARP1 | J03473 /FEATURE=mRNA /DEFINITION=HUMRISDAD Human poly(ADP-ribose) synthetase mRNA, complete cds | 120 | Y |
| 5 | 38242_at | BLNK | Cluster Incl. AF068180:Homo sapiens B cell linker protein BLNK mRNA, alternatively spliced, complete cds /cds=(153,1523) /gb=AF068180 /gi=3406748 /ug=Hs.167746 /len=1790 | 117 |  |
| 6 | 34168_at | DNTT | Cluster Incl. M11722:Human terminal transferase mRNA, complete cds /cds=(328,1854) /gb=M11722 /gi=339436 /ug=Hs.234772 /len=2068 | 108 |  |
| 7 | 35974_at | LRMP | Cluster Incl. U10485:Human lymphoid-restricted membrane protein (Jaw1) mRNA, complete cds /cds=(574,2241) /gb=U10485 /gi=505685 /ug=Hs.40202 /len=2417 | 88 |  |
| 8 | 40745_at | AP1B1 | Cluster Incl. L13939:Homo sapiens beta adaptin (BAM22) mRNA, complete cds /cds=(46,2895) /gb=L13939 /gi=4079593 /ug=Hs.89576 /len=3859 | 75 |  |
| 9 | 37039_at | HLA-DRA | Cluster Incl. J00194:human hla-dr antigen alpha-chain mrna & ivs fragments /cds=(26,790) /gb=J00194 /gi=188231 /ug=Hs.76807 /len=1199 | 36 |  |
| 10 | 41146_at | PARP1 | Cluster Incl. J03473:Human poly(ADP-ribose) synthetase mRNA, complete cds /cds=(95,3139) /gb=J03473 /gi=337423 /ug=Hs.177766 /len=3795 | 35 | Y |
| 11 | 37680_at | AKAP12 | Cluster Incl. U81607:Homo sapiens gravin mRNA, complete cds /cds=(191,5536) /gb=U81607 /gi=2218076 /ug=Hs.788 /len=6596 | 35 |  |
| 12 | 36008_at | PTP4A3 | Cluster Incl. AF041434:Homo sapiens potentially prenylated protein tyrosine phosphatase hPRL-3 mRNA, complete cds /cds=(237,758) /gb=AF041434 /gi=3406429 /ug=Hs.43666 /len=1006 | 33 |  |
| 13 | 31863_at | RRP1B | Cluster Incl. D80001:Human mRNA for KIAA0179 gene, partial cds /cds=(0,2288) /gb=D80001 /gi=1136417 /ug=Hs.152629 /len=4994 | 31 |  |
| 14 | 2031_s_at | CDKN1A | U03106 /FEATURE= /DEFINITION=HSU03106 Human wild-type p53 activated fragment-1 (WAF1) mRNA, complete cds | 29 | Y |
| 15 | 39507_at | OGT | Cluster Incl. AL050366:Homo sapiens mRNA; cDNA DKFZp564A126 (from clone DKFZp564A126) /cds=UNKNOWN /gb=AL050366 /gi=4914599 /ug=Hs.100293 /len=5508 | 24 |  |
| 16 | 32794_g_at | IL23A | Cluster Incl. X00437:Human mRNA for T-cell specific protein /cds=(37,975) /gb=X00437 /gi=36748 /ug=Hs.2003 /len=1151 | 23 |  |
| 17 | 38774_at | STX7 | Cluster Incl. U77942:Human syntaxin 7 mRNA, complete cds /cds=(79,864) /gb=U77942 /gi=2337919 /ug=Hs.8906 /len=1614 | 21 |  |
| 18 | 41165_g_at | IGHG1 | Cluster Incl. X67301:H.sapiens mRNA for IgM heavy chain constant region (Ab63) /cds=(0,1361) /gb=X67301 /gi=38407 /ug=Hs.179543 /len=1453 | 17 |  |
| 19 | 39168_at | DHRSX | Cluster Incl. AB018328:Homo sapiens mRNA for KIAA0785 protein, complete cds /cds=(201,2285) /gb=AB018328 /gi=3882290 /ug=Hs.9933 /len=4485 | 14 |  |
| 20 | 1520_s_at | IL1B | J05008 /FEATURE=expanded_cds /DEFINITION=HUMEDN1B Homo sapiens endothelin-1 (EDN1) gene, complete cds | 12 | Y |
| 21 | 40519_at | PTPRC | Cluster Incl. Y00638:Human mRNA for leukocyte common antigen (T200) /cds=(86,4000) /gb=Y00638 /gi=34280 /ug=Hs.170121 /len=4315 | 9 |  |
| 22 | 37420_i_at | HLA-F | Cluster Incl. AL022723:dJ377H14.9 (major histocompatibility complex, class I, F (CDA12)) /cds=(97,1185) /gb=AL022723 /gi=5002624 /ug=Hs.110309 /len=1303 | 9 |  |
| 23 | 1971_g_at | FHIT | U46922 /FEATURE= /DEFINITION=HSU46922 Human FHIT mRNA, complete cds | 8 | Y |
| 24 | 34224_at | FADS1 | Cluster Incl. AC004770:Homo sapiens chromosome 11, BAC CIT-HSP-311e8 (BC269730) containing the hFEN1 gene /cds=(0,1058) /gb=AC004770 /gi=3212836 /ug=Hs.21765 /len=1059 | 8 |  |
| 25 | 39345_at | NPC2 | Cluster Incl. AI525834:PT1.3_06_D01.r Homo sapiens cDNA, 5 end /clone_end=5 /gb=AI525834 /gi=4439969 /ug=Hs.119529 /len=951 | 7 |  |
| 26 | 34210_at |  | Cluster Incl. N90866:zb11b10.s1 Homo sapiens cDNA, 3 end /clone=IMAGE-301723 /clone_end=3 /gb=N90866 /gi=1444193 /ug=Hs.214742 /len=577 | 7 |  |
| 27 | 40775_at | ITM2A | Cluster Incl. AL021786:Human DNA sequence from PAC 696H22 on chromosome Xq21.1-21.2. Contains a mouse E25 like gene, a Kinesin like pseudogene and ESTs /cds=(0,680) /gb=AL021786 /gi=2853186 /ug=Hs.17109 /len=1389 | 7 |  |
| 28 | 38018_g_at | CD79A | Cluster Incl. U05259:Human MB-1 gene, complete cds /cds=(36,716) /gb=U05259 /gi=452561 /ug=Hs.79630 /len=1107 | 7 |  |
| 29 | 37780_at | PCLO | Cluster Incl. AB011131:Homo sapiens mRNA for KIAA0559 protein, partial cds /cds=(0,3640) /gb=AB011131 /gi=3043641 /ug=Hs.12376 /len=5639 | 7 |  |
| 30 | 1105_s_at | IL23A | M12886 /FEATURE= /DEFINITION=HUMTCBYY Human T-cell receptor active beta-chain mRNA, complete cds | 7 |  |
| 31 | 41462_at | SNX2 | Cluster Incl. AF065482:Homo sapiens sorting nexin 2 (SNX2) mRNA, complete cds /cds=(29,1588) /gb=AF065482 /gi=3152937 /ug=Hs.11183 /len=2037 | 7 |  |
| 32 | 39114_at | C10orf10 | Cluster Incl. AB022718:Homo sapiens mRNA for DEPP (decidual protein induced by progesterone), complete cds /cds=(218,856) /gb=AB022718 /gi=4204189 /ug=Hs.93675 /len=2114 | 6 |  |
| 33 | 39056_at | PAICS | Cluster Incl. X53793:H.sapiens ADE2H1 mRNA showing homologies to SAICAR synthetase and AIR carboxylase of the purine pathway (EC 6.3.2.6, EC 4.1.1.21) /cds=(24,1301) /gb=X53793 /gi=28383 /ug=Hs.117950 /len=1426 | 5 |  |
| 34 | 37890_at | CD47 | Cluster Incl. X69398:H.sapiens mRNA for OA3 antigenic surface determinant /cds=(106,1077) /gb=X69398 /gi=396175 /ug=Hs.82685 /len=1285 | 5 |  |
| 35 | 41819_at | FYB | Cluster Incl. AF001862:Homo sapiens FYN binding protein mRNA, complete cds /cds=(67,2418) /gb=AF001862 /gi=2232149 /ug=Hs.58435 /len=2578 | 5 |  |
| 36 | 36524_at | ARHGEF4 | Cluster Incl. AB029035:Homo sapiens mRNA for KIAA1112 protein, partial cds /cds=(0,2086) /gb=AB029035 /gi=5689560 /ug=Hs.6066 /len=3800 | 5 |  |
| 37 | 1077_at | RAG1 | M29474 /FEATURE=mRNA /DEFINITION=HUMRAG1 Human recombination activating protein (RAG-1) gene, complete cds | 5 |  |
| 38 | 35238_at | TRAF5 | Cluster Incl. AB000509:Homo sapiens mRNA for TRAF5, complete cds /cds=(54,1727) /gb=AB000509 /gi=2982670 /ug=Hs.29736 /len=3968 | 5 |  |
| 39 | 32542_at | FHL1 | Cluster Incl. AF063002:Homo sapiens LIM protein SLIMMER mRNA, complete cds /cds=(84,1055) /gb=AF063002 /gi=3859848 /ug=Hs.75329 /len=2042 | 4 |  |
| 40 | 40729_s_at |  | Cluster Incl. Y14768:Homo sapiens DNA, cosmid clones TN62 and TN82 /cds=(10,744) /gb=Y14768 /gi=3805800 /ug=Hs.890 /len=896 | 4 |  |
| 41 | 40272_at | CRMP1 | Cluster Incl. D78012:Homo sapiens mRNA for dihydropyrimidinase related protein-1, complete cds /cds=(150,1868) /gb=D78012 /gi=1330237 /ug=Hs.155392 /len=2842 | 4 |  |
| 42 | 41406_at | INTS3 | Cluster Incl. AL080172:Homo sapiens mRNA; cDNA DKFZp434G231 (from clone DKFZp434G231) /cds=UNKNOWN /gb=AL080172 /gi=5262642 /ug=Hs.105894 /len=3406 | 4 |  |
| 43 | 36239_at | POU2AF1 | Cluster Incl. Z49194:H.sapiens mRNA for oct-binding factor /cds=(523,1293) /gb=Z49194 /gi=974830 /ug=Hs.2407 /len=3301 | 4 |  |
| 44 | 41425_at | FLI1 | Cluster Incl. M98833:Human ERGB transcription factor (FLI-1 homolog) mRNA, complete cds /cds=(172,1527) /gb=M98833 /gi=182188 /ug=Hs.108043 /len=2954 | 4 | Y |
| 45 | 38994_at | SOCS2 | Cluster Incl. AF037989:Homo sapiens STAT-induced STAT inhibitor-2 mRNA, complete cds /cds=(317,913) /gb=AF037989 /gi=3265032 /ug=Hs.110776 /len=1937 | 4 |  |
| 46 | 41200_at | SCARB1 | Cluster Incl. Z22555:H.sapiens encoding CLA-1 mRNA /cds=(69,1598) /gb=Z22555 /gi=397606 /ug=Hs.180616 /len=2552 | 4 |  |
| 47 | 1488_at | PTPRK | L77886 /FEATURE= /DEFINITION=HUMPTPC Human protein tyrosine phosphatase mRNA, complete cds | 4 | Y |
| 48 | 39003_at | PTTG1IP | Cluster Incl. Z50022:H.sapiens mRNA for surface glycoprotein /cds=(93,635) /gb=Z50022 /gi=1107702 /ug=Hs.111126 /len=2617 | 3 |  |
| 49 | 37759_at | LAPTM5 | Cluster Incl. U51240:Human lysosomal-associated multitransmembrane protein (LAPTm5) mRNA, complete cds /cds=(75,863) /gb=U51240 /gi=1255239 /ug=Hs.79356 /len=2232 | 3 |  |
| 50 | 36383_at | ERG | Cluster Incl. M17254:Human erg2 gene encoding erg2 protein, complete cds /cds=(0,1388) /gb=M17254 /gi=182186 /ug=Hs.159432 /len=1389 | 3 | Y |

**Table S7** Description of 50 top-ranked genes for the colon tumor dataset

| No. | Access No. | Gene symbol | Description | Frequency | Is cancer gene? |
| --- | --- | --- | --- | --- | --- |
| 1 | M26383 | IL8 | Human monocyte-derived neutrophil-activating protein (MONAP) mRNA, complete cds. | 64 |  |
| 2 | M80815 | FUCA1 | H.sapiens a-L-fucosidase gene, exon 7 and 8, and complete cds. | 32 |  |
| **3** | **M76378** | **CSRP1** | Human cysteine-rich protein (CRP) gene, exons 5 and 6. | 31 |  |
| **4** | **M76378** | **CSRP1** | Human cysteine-rich protein (CRP) gene, exons 5 and 6. | 31 |  |
| 5 | J05032 | DARS | Human aspartyl-tRNA synthetase alpha-2 subunit mRNA, complete cds. | 22 |  |
| 6 | R87126 | MYH9 | MYOSIN HEAVY CHAIN, NONMUSCLE (Gallus gallus) | 17 | Y |
| 7 | R36977 | GTF3A | P03001 TRANSCRIPTION FACTOR IIIA; | 14 |  |
| 8 | D16294 | ACAA2 | Human mRNA for mitochondrial 3-oxoacyl-CoA thiolase, complete cds. | 14 |  |
| 9 | H43887 | CFD | COMPLEMENT FACTOR D PRECURSOR (Homo sapiens) | 14 |  |
| 10 | H64489 | TSPAN1 | LEUKOCYTE ANTIGEN CD37 (Homo sapiens) | 12 |  |
| 11 | H20709 | MYL6 | MYOSIN LIGHT CHAIN ALKALI, SMOOTH-MUSCLE ISOFORM (HUMAN). | 12 |  |
| 12 | X14958 | HMGA1 | Human hmgI mRNA for high mobility group protein Y. | 11 | Y |
| 13 | T51023 | HSP90AB1 | HEAT SHOCK PROTEIN HSP 90-BETA (HUMAN). | 11 |  |
| 14 | Z50753 | GUCA2B | H.sapiens mRNA for GCAP-II/uroguanylin precursor. | 11 |  |
| **15** | **M76378** | **CSRP1** | Human cysteine-rich protein (CRP) gene, exons 5 and 6. | 10 |  |
| 16 | X54942 | CKS2 | H.sapiens ckshs2 mRNA for Cks1 protein homologue. | 9 |  |
| 17 | X12671 | HNRNPA1 | Human gene for heterogeneous nuclear ribonucleoprotein (hnRNP) core protein A1. | 8 |  |
| 18 | M26697 | NPM1 | Human nucleolar protein (B23) mRNA, complete cds. | 8 | Y |
| 19 | M36634 | VIP | Human vasoactive intestinal peptide (VIP) mRNA, complete cds. | 7 |  |
| 20 | H40095 | MIF (GLIF) (MMIF) | MACROPHAGE MIGRATION INHIBITORY FACTOR (HUMAN). | 6 |  |
| 21 | M22382 | HSPD1 | MITOCHONDRIAL MATRIX PROTEIN P1 PRECURSOR (HUMAN). | 6 |  |
| 22 | X63629 | CDH3 | H.sapiens mRNA for p cadherin. | 6 |  |
| 23 | T71025 | MT1G | Human (HUMAN); | 6 |  |
| 24 | H72234 | APEX1 | DNA-(APURINIC OR APYRIMIDINIC SITE) LYASE (HUMAN). | 5 | Y |
| 25 | T86749 | TSPAN31 | Human (clone PSK-J3) cyclin-dependent protein kinase mRNA, complete cds. | 5 |  |
| 26 | T86473 | NME1 | NUCLEOSIDE DIPHOSPHATE KINASE A (HUMAN). | 5 | Y |
| 27 | H87135 | C7orf47 | IMMEDIATE-EARLY PROTEIN IE180 (Pseudorabies virus) | 5 |  |
| 28 | D14812 | MORF4L2 | Human mRNA for ORF, complete cds. | 4 | Y |
| 29 | R55310 | UQCRC1 | S36390 MITOCHONDRIAL PROCESSING PEPTIDASE; | 4 |  |
| 30 | U30825 | SFRS9 | Human splicing factor SRp30c mRNA, complete cds. | 4 |  |
| 31 | T51571 | S100A11 | P24480 CALGIZZARIN. | 4 | Y |
| 32 | M63391 | DES | Human desmin gene, complete cds. | 3 |  |
| 33 | T59162 | SELENBP1 | SELENIUM-BINDING PROTEIN (Mus musculus) | 3 |  |
| 34 | X70326 | MARCKSL1 | H.sapiens MacMarcks mRNA. | 3 |  |
| 35 | D59253 | NCBP2 | Human mRNA for NCBP interacting protein 1. | 3 |  |
| 36 | H08393 | WDR77 | COLLAGEN ALPHA 2(XI) CHAIN (Homo sapiens) | 3 |  |
| 37 | H89087 | RNPS1 | SPLICING FACTOR SC35 (Homo sapiens) | 3 |  |
| 38 | H70425 |  | INTERFERON-ALPHA RECEPTOR PRECURSOR (Homo sapiens) | 3 |  |
| 39 | T51858 | EIF4B | EUKARYOTIC INITIATION FACTOR 4B (Homo sapiens) | 3 |  |
| 40 | U04953 | IARS | Human isoleucyl-tRNA synthetase mRNA, complete cds. | 3 |  |
| 41 | L05144 | PCK1 | PHOSPHOENOLPYRUVATE CARBOXYKINASE, CYTOSOLIC (HUMAN);contains Alu repetitive element;contains element PTR5 repetitive element. | 3 |  |
| 42 | M88279 | FKBP4 | P59 PROTEIN (HUMAN); | 3 |  |
| 43 | T51493 | PPP2R5C | Homo sapiens PP2A B56-gamma1 mRNA, 3'' end of cds. | 3 |  |
| 44 | T89115 | HNRNPH1 (HNRPH, HNRPH1) | HETEROGENEOUS NUCLEAR RIBONUCLEOPROTEIN K (Homo sapiens) | 3 |  |
| 45 | X12369 | TPM1 | TROPOMYOSIN ALPHA CHAIN, SMOOTH MUSCLE (HUMAN). | 3 | Y |
| 46 | M55265 | CSNK2A1 | Human casein kinase II alpha subunit mRNA, complete cds. | 3 | Y |
| 47 | M59040 | CD44 | Human cell adhesion molecule (CD44) mRNA, complete cds. | 3 |  |
| 48 | U34074 | AKAP1 | Human A kinase anchor protein S-AKAP84 mRNA, nuclear gene encoding mitochondrial protein, complete cds. | 3 |  |
| 49 | L19437 | TALDO1 | TRANSALDOLASE (HUMAN);contains Alu repetitive element;contains PTR5 repetitive element. | 2 |  |
| 50 | D00761 | PSMB1 | PROTEASOME COMPONENT C5 (HUMAN). | 2 |  |

**Table S8** Description of 50 top-ranked genes for the DLBCL dataset

| No. | Probe No. | Gene symbol | Description | Frequency | Is cancer gene? |
| --- | --- | --- | --- | --- | --- |
| 1 | Z35227_at | RHOH | TTF mRNA for small G protein | 201 |  |
| 2 | D78134_at | CIRBP | YWHAZ Tyrosine 3-monooxygenase/tryptophan 5-monooxygenase activation protein, zeta polypeptide | 153 |  |
| 3 | D55716_at | MCM7 | DNA REPLICATION LICENSING FACTOR CDC47 HOMOLOG | 128 |  |
| 4 | D87119_at | TRIB2 | Cancellous bone osteoblast mRNA for GS3955 | 71 |  |
| 5 | M94880_f_at | HLA-A | HLA-A MHC class I protein HLA-A (HLA-A28,-B40, -Cw3) | 67 |  |
| 6 | D38076_at | RANBP1 | RANBP1 RAN binding protein 1 | 60 |  |
| 7 | L02426_at | PSMC1 | 26S PROTEASE REGULATORY SUBUNIT 4 | 53 |  |
| 8 | X67951_at | PRDX1 | PAGA Proliferation-associated gene A (natural killer-enhancing factor A) | 50 |  |
| 9 | M63835_at | FCGR1A | HIGH AFFINITY IMMUNOGLOBULIN GAMMA FC RECEPTOR I "A FORM" PRECURSOR | 50 |  |
| 10 | M63138_at | CTSD | CTSD Cathepsin D (lysosomal aspartyl protease) | 25 |  |
| 11 | D83597_at | CD180 | RP105 | 24 |  |
| 12 | L25876_at | CDKN3 | Protein tyrosine phosphatase (CIP2)mRNA | 20 |  |
| 13 | X02152_at | LDHA | LDHA Lactate dehydrogenase A | 20 |  |
| 14 | L42324_at | GPR18 | (clone GPCR W) G protein-linked receptor gene (GPCR) gene, 5'' end of cds | 20 |  |
| 15 | Z49099_at | SMS | Spermine synthase | 19 |  |
| 16 | U72935_cds3_s_at | ATRX | ATRX gene (putative DNA dependent ATPase and helicase) extracted from Human putative DNA dependent ATPase and helicase (ATRX) gene | 18 | Y |
| 17 | V00594_at | MT2A | Metallothionein isoform 2 | 18 |  |
| 18 | X56494_at | PKM2 | PKM2 Pyruvate kinase, muscle | 17 |  |
| 19 | X16983_at | ITGA4 | ITGA4 Integrin, alpha 4 (antigen CD49D, alpha 4 subunit of VLA-4 receptor) | 17 | Y |
| 20 | X17567_s_at | SNRPB | SNRPB Small nuclear ribonucleoprotein polypeptides B and B1 | 15 |  |
| 21 | HG4716-HT5158_at |  | Guanosine 5''-Monophosphate Synthase | 15 |  |
| 22 | U81375_at | SLC29A1 | Placental equilibrative nucleoside transporter 1 (hENT1) mRNA | 14 |  |
| 23 | D13633_at | DLGAP5 | KIAA0008 gene | 13 |  |
| 24 | M22760_at | COX5A | CYTOCHROME C OXIDASE POLYPEPTIDE VA PRECURSOR | 13 |  |
| 25 | U14518_at | CENPA | CENPA Centromere protein A (17kD) | 12 |  |
| 26 | D84557_at | MCM6 | P105MCM mRNA | 12 |  |
| 27 | Z21966_at | POU6F1 | POU6F1 POU homeobox protein | 11 |  |
| 28 | HG2279-HT2375_at | TPI1 (TPI) | Triosephosphate Isomerase | 9 |  |
| 29 | J04173_at | PGAM1 | PGAM1 Phosphoglycerate mutase 1 (brain) | 9 |  |
| 30 | X59543_at | RRM1 | RIBONUCLEOSIDE-DIPHOSPHATE REDUCTASE M1 CHAIN | 9 |  |
| 31 | X74801_at | CCT3 | T-COMPLEX PROTEIN 1, GAMMA SUBUNIT | 9 |  |
| 32 | HG1980-HT2023_at |  | Tubulin, Beta 2 | 9 |  |
| 33 | U09587_at | GARS | GARS Glycyl-tRNA synthetase | 9 |  |
| 34 | U48296_at | PTP4A1 | Protein tyrosine phosphatase PTPCAAX1 (hPTPCAAX1) mRNA | 9 |  |
| 35 | Z70723_at | PON1 | SERUM PARAOXONASE/ARYLESTERASE | 9 | Y |
| 36 | X12447_at | ALDOA | ALDOA Aldolase A | 8 |  |
| 37 | M19645_at | HSPA5 | 78 KD GLUCOSE REGULATED PROTEIN PRECURSOR | 8 |  |
| 38 | D82348_at | ATIC | 5-aminoimidazole-4-carboxamide-1-beta-D-ribonucleoti de transformylase/inosinicase | 7 |  |
| 39 | M13792_at | ADA | ADA Adenosine deaminase | 7 |  |
| 40 | Z11793_at | SEPP1 | Selenoprotein P | 7 |  |
| 41 | U90313_at | GSTO1 | Glutathione-S-transferase homolog mRNA | 7 |  |
| 42 | D38048_at | PSMB7 | Proteasome subunit z | 7 |  |
| 43 | HG2874-HT3018_at | rpl36a | Ribosomal Protein L39 Homolog | 7 |  |
| 44 | M63379_at | CLU | CLU Clusterin (complement lysis inhibitor; testosterone-repressed prostate message 2; apolipoprotein J) | 7 | Y |
| 45 | U62293_rna1_s_at | LIMK1 | LIMK1 gene (LIM-kinase1) extracted from Human LIM-kinase1 and alternatively spliced LIM-kinase1 (LIMK1) gene | 7 |  |
| 46 | D80008_at | GINS1 | KIAA0186 gene | 7 |  |
| 47 | L19686_rna1_at | MIF | Macrophage migration inhibitory factor (MIF) gene | 6 | Y |
| 48 | D28473_s_at | IARS | IARS Isoleucine-tRNA synthetase | 6 |  |
| 49 | M29536_at | EIF2S2 | Translational initiation factor 2 beta subunit (elF-2-beta) mRNA | 6 |  |
| 50 | J03507_at | C7 | C7 Complement component 7 | 6 |  |

**Table S9** Description of 50 top-ranked genes for the leukemia dataset

| No. | Probe No. | Gene symbol | Description | Frequency | Is cancer gene? |
| --- | --- | --- | --- | --- | --- |
| 1 | M23197_at | CD33 | CD33 CD33 antigen (differentiation antigen) | 82 |  |
| 2 | X95735_at | ZYX | Zyxin | 74 | Y |
| 3 | M27891_at | CST3 | CST3 Cystatin C (amyloid angiopathy and cerebral hemorrhage) | 72 |  |
| 4 | M31523_at | TCF3 | TCF3 Transcription factor 3 (E2A immunoglobulin enhancer binding factors E12/E47) | 22 | Y |
| 5 | U46499_at | MGST1 | GLUTATHIONE S-TRANSFERASE, MICROSOMAL | 18 |  |
| 6 | L09209_s_at | APLP2 | APLP2 Amyloid beta (A4) precursor-like protein 2 | 15 |  |
| 7 | M92287_at | CCND3 | CCND3 Cyclin D3 | 9 | Y |
| 8 | X59417_at | PSMA6 | PROTEASOME IOTA CHAIN | 6 |  |
| 9 | HG1612-HT1612_at | MARCKSL1 (MLP, MRP) | Macmarcks | 5 |  |
| 10 | J05243_at | SPTAN1 | SPTAN1 Spectrin, alpha, non-erythrocytic 1 (alpha-fodrin) | 5 |  |
| 11 | D26308_at | BLVRB | NADPH-flavin reductase | 5 |  |
| 12 | M84526_at | CFD | DF D component of complement (adipsin) | 5 |  |
| 13 | X62654_rna1_at | CD63 | ME491 gene extracted from H.sapiens gene for Me491/CD63 antigen | 5 |  |
| 14 | Y07604_at | NME4 | Nucleoside-diphosphate kinase | 4 |  |
| 15 | L07633_at | PSME1 | INTERFERON GAMMA UP-REGULATED I-5111 PROTEIN PRECURSOR | 4 |  |
| 16 | D88422_at | CSTA | CYSTATIN A | 4 |  |
| 17 | M63379_at | CLU | CLU Clusterin (complement lysis inhibitor; testosterone-repressed prostate message 2; apolipoprotein J) | 4 | Y |
| 18 | Z15115_at | TOP2B | TOP2B Topoisomerase (DNA) II beta (180kD) | 4 |  |
| 19 | M11722_at | DNTT (TDT) | Terminal transferase mRNA | 4 |  |
| 20 | U05259_rna1_at | CD79A | MB-1 gene | 4 |  |
| 21 | U77948_at | GTF2I | KAI1 Kangai 1 (suppression of tumorigenicity 6, prostate; CD82 antigen (R2 leukocyte antigen, antigen detected by monoclonal and antibody IA4)) | 4 |  |
| 22 | U94855_at | EIF3F | Translation initiation factor 3 47 kDa subunit mRNA | 4 |  |
| 23 | M31166_at | PTX3 | PTX3 Pentaxin-related gene, rapidly induced by IL-1 beta | 4 |  |
| 24 | U77604_at | MGST2 | Microsomal glutathione S-transferase (GST-II) mRNA | 4 |  |
| 25 | M91432_at | ACADM | ACADM Acyl-Coenzyme A dehydrogenase, C-4 to C-12 straight chain | 4 |  |
| 26 | X66401_cds1_at | TAP2 | LMP2 gene extracted from H.sapiens genes TAP1, TAP2, LMP2, LMP7 and DOB | 3 |  |
| 27 | L47738_at | CYFIP2 | Inducible protein mRNA | 3 |  |
| 28 | X85116_rna1_s_at | STOM | Epb72 gene exon 1 | 3 |  |
| 29 | X68560_at | SP3 | SP3 Sp3 transcription factor | 3 |  |
| 30 | U97105_at | DPYSL2 (CRMP2) | Dihydropyrimidinase related protein-2 | 3 |  |
| 31 | U16954_at | MLLT11 | (AF1q) mRNA | 3 |  |
| 32 | M33680_at | CD81 | 26-kDa cell surface protein TAPA-1 mRNA | 3 |  |
| 33 | X51521_at | EZR | VIL2 Villin 2 (ezrin) | 3 |  |
| 34 | D26156_s_at | SMARCA4 | Transcriptional activator hSNF2b | 3 | Y |
| 35 | U70867_at | SLCO2A1 | Prostaglandin transporter hPGT mRNA | 3 |  |
| 36 | L05148_at | ZAP70 | Protein tyrosine kinase related mRNA sequence | 3 |  |
| 37 | U72936_s_at | ATRX | X-LINKED HELICASE II | 3 | Y |
| 38 | Y00787_s_at | IL8 | INTERLEUKIN-8 PRECURSOR | 3 |  |
| 39 | D86967_at | EDEM1 | KIAA0212 gene | 3 |  |
| 40 | M63138_at | CTSD | CTSD Cathepsin D (lysosomal aspartyl protease) | 3 |  |
| 41 | X64364_at | BSG | BSG Basigin | 3 |  |
| 42 | M96803_at | SPTBN1 | SPTBN1 Spectrin, beta, non-erythrocytic 1 | 3 |  |
| 43 | Z14982_rna1_at | PSM88 | MHC-encoded proteasome subunit gene LAMP7-E1 gene (proteasome subunit LMP7) extracted from H.sapiens gene for major histocompatibility complex encoded proteasome subunit LMP7 | 3 |  |
| 44 | M29696_at | IL7R | IL7R Interleukin 7 receptor | 2 |  |
| 45 | U10485_at | LRMP | Lymphoid-restricted membrane protein (Jaw1) mRNA | 2 |  |
| 46 | U18271_cds3_s_at | TMPO | Thymopoietin (TMPO) gene | 2 |  |
| 47 | D50918_at | SEPT6 | KIAA0128 gene, partial cds | 2 |  |
| 48 | X63753_at | SON | SON SON DNA binding protein | 2 |  |
| 49 | D86970_at | MYO18A | KIAA0216 gene | 2 |  |
| 50 | U29175_at | SMARCA4 | Transcriptional activator hSNF2b | 2 | Y |

**Table S10** Description of 50 top-ranked genes for the prostate dataset

| No. | Probe No. | Gene symbol | Description | Frequency | Is cancer gene? |
| --- | --- | --- | --- | --- | --- |
| 1 | 37639_at | HPN | Cluster Incl. X07732:Human hepatoma mRNA for serine protease hepsin /cds=UNKNOWN /gb=X07732 /gi=32063 /ug=Hs.823 /len=2363 | 285 |  |
| 2 | 41504_s_at | MAF | Cluster Incl. AF055376:Homo sapiens short form transcription factor C-MAF (c-maf) mRNA, complete cds /cds=(807,1928) /gb=AF055376 /gi=3335147 /ug=Hs.30250 /len=4246 | 281 | Y |
| 3 | 2041_i_at | ABL1 | M14752 /FEATURE= /DEFINITION=HUMABLA Human c-abl gene, complete cds | 164 | Y |
| 4 | 34213_at | WWC1 | Cluster Incl. AB020676:Homo sapiens mRNA for KIAA0869 protein, partial cds /cds=(0,2667) /gb=AB020676 /gi=4240226 /ug=Hs.21543 /len=3408 | 67 |  |
| 5 | 40436_g_at | SLC25A6 | Cluster Incl. J03592:Human ADP/ATP translocase mRNA, 3 end, clone pHAT8 /cds=(0,788) /gb=J03592 /gi=339722 /ug=Hs.164280 /len=1116 | 34 |  |
| 6 | 40024_at | STAC | Cluster Incl. D86640:Homo sapiens mRNA for stac, complete cds /cds=(39,1247) /gb=D86640 /gi=1799567 /ug=Hs.56045 /len=2963 | 16 |  |
| 7 | 40282_s_at | CFD | Cluster Incl. M84526:Human adipsin/complement factor D mRNA, complete cds /cds=(54,740) /gb=M84526 /gi=178625 /ug=Hs.155597 /len=1071 | 15 |  |
| 8 | 32786_at | JUNB | Cluster Incl. X51345:Human jun-B mRNA for JUN-B protein /cds=(253,1296) /gb=X51345 /gi=34014 /ug=Hs.198951 /len=1797 | 14 | Y |
| 9 | 38098_at | LPIN1 | Cluster Incl. D80010:Human mRNA for KIAA0188 gene, partial cds /cds=(0,2700) /gb=D80010 /gi=1136435 /ug=Hs.81412 /len=5307 | 9 |  |
| 10 | 863_g_at | SERPINB5 | U04313 /FEATURE= /DEFINITION=HSU04313 Human maspin mRNA, complete cds | 6 | Y |
| 11 | 39582_at | CYLD | Cluster Incl. AL050166:Homo sapiens mRNA; cDNA DKFZp586D1122 (from clone DKFZp586D1122) /cds=UNKNOWN /gb=AL050166 /gi=4884381 /ug=Hs.26295 /len=2654 | 6 | Y |
| 12 | 914_g_at | ERG | M21535 /FEATURE= /DEFINITION=HUMERG11 Human erg protein (ets-related gene) mRNA, complete cds | 6 | Y |
| 13 | 40074_at | MTHFD2 | Cluster Incl. X16396:Human mRNA for NAD-dependent methylene tetrahydrofolate dehydrogenase cyclohydrolase (EC 1.5.1.15) /cds=(15,1049) /gb=X16396 /gi=35070 /ug=Hs.154672 /len=2102 | 5 |  |
| 14 | 37068_at | PLA2G7 | Cluster Incl. U24577:Human LDL-phospholipase A2 mRNA, complete cds /cds=(216,1541) /gb=U24577 /gi=1314245 /ug=Hs.93304 /len=1561 | 4 |  |
| 15 | 39756_g_at | LOC646408 | Cluster Incl. Z93930:Human DNA sequence from clone 292E10 on chromosome 22q11-12. Contains the XBP1 gene for X-box binding protein 1 (TREB5), ESTs, STSs, GSSs and a putative CpG island /cds=(30,815) /gb=Z93930 /gi=4775603 /ug=Hs.149923 /len=1802 | 4 |  |
| 16 | 34775_at | TSPAN1 | Cluster Incl. AF065388:Homo sapiens tetraspan NET-1 mRNA, complete cds /cds=(121,846) /gb=AF065388 /gi=3152700 /ug=Hs.38972 /len=1278 | 4 |  |
| 17 | 33386_at |  | Cluster Incl. Z97630:Human DNA sequence from clone 466N1 on chromosome 22q12-13 Contains H1F0(H1 histone family, member 0) gene, 2-amino-3-ketobutyrate -CoA ligase( nuclear gene encoding mitochondrial protein), GALR3 (galanin receptor) gene, ESTs, GSSs and CpG islands /cds=(381,965) /gb=Z97630 /gi=4582128 /ug=Hs.226117 /len=2527 | 4 |  |
| 18 | 1980_s_at | NME1 | X58965 /FEATURE= /DEFINITION=HSNM23H2G H.sapiens RNA for nm23-H2 gene | 4 | Y |
| 19 | 1708_at | MAPK10 | U07620 /FEATURE= /DEFINITION=HSU07620 Human MAP kinase mRNA, complete cds | 3 |  |
| 20 | 41288_at |  | Cluster Incl. AL036744:DKFZp564I1663_r1 Homo sapiens cDNA, 5 end /clone=DKFZp564I1663 /clone_end=5 /gb=AL036744 /gi=5927888 /ug=Hs.236327 /len=617 | 3 |  |
| 21 | 38087_s_at | S100A4 | Cluster Incl. W72186:zd69b10.s1 Homo sapiens cDNA, 3 end /clone=IMAGE-345883 /clone_end=3 /gb=W72186 /gi=1382635 /ug=Hs.81256 /len=598 | 3 | Y |
| 22 | 36666_at | P4HB | Cluster Incl. M22806:Human prolyl 4-hydroxylase beta-subunit and disulfide isomerase (P4HB) gene /cds=(66,1592) /gb=M22806 /gi=487831 /ug=Hs.75655 /len=2438 | 3 | Y |
| 23 | 556_s_at | GSTM4 | M96233 /FEATURE=expanded_cds /DEFINITION=HUMGSTM4A Human glutathione transferase class mu number 4 (GSTM4) gene, complete cds | 3 |  |
| 24 | 37599_at | AOX1 | Cluster Incl. AF017060:untitled /cds=(298,4314) /gb=AF017060 /gi=2343154 /ug=Hs.81047 /len=5125 | 3 |  |
| 25 | 33328_at | HEG1 | Cluster Incl. W28612:49b3 Homo sapiens cDNA /gb=W28612 /gi=1308560 /ug=Hs.184724 /len=809 | 3 |  |
| 26 | 41585_at | KIAA0746 | Cluster Incl. AB018289:Homo sapiens mRNA for KIAA0746 protein, partial cds /cds=(0,3091) /gb=AB018289 /gi=3882212 /ug=Hs.49500 /len=4086 | 3 |  |
| 27 | 39705_at | SIN3B | Cluster Incl. AB014600:Homo sapiens mRNA for KIAA0700 protein, partial cds /cds=(0,3393) /gb=AB014600 /gi=3327213 /ug=Hs.13999 /len=5020 | 3 |  |
| 28 | 36624_at | IMPDH2 | Cluster Incl. L33842:Homo sapiens (clone FFE-7) type II inosine monophosphate dehydrogenase (IMPDH2) gene, exons 1-13, complete cds /cds=(102,1646) /gb=L33842 /gi=602457 /ug=Hs.75432 /len=1688 | 3 |  |
| 29 | 38684_at | ATP2C1 | Cluster Incl. AJ010953:Homo sapiens mRNA for putative Ca2+-transporting ATPase, partial /cds=(0,1491) /gb=AJ010953 /gi=3646133 /ug=Hs.106778 /len=2134 | 3 |  |
| 30 | 31609_s_at | PCOLCE | Cluster Incl. L33799:Human procollagen C-proteinase enhancer protein (PCOLCE) mRNA, complete cds /cds=(60,1409) /gb=L33799 /gi=642907 /ug=Hs.202097 /len=1480 | 3 |  |
| 31 | 32225_at | ATP1A1 | Cluster Incl. X04297:Human mRNA for Na,K-ATPase alpha-subunit /cds=(318,3389) /gb=X04297 /gi=28926 /ug=Hs.190703 /len=4108 | 2 |  |
| 32 | 34853_at | FLRT2 | Cluster Incl. AB007865:Homo sapiens KIAA0405 mRNA, complete cds /cds=(1124,3106) /gb=AB007865 /gi=2662090 /ug=Hs.48998 /len=7527 | 2 |  |
| 33 | 769_s_at | ANXA2 | D00017 /FEATURE= /DEFINITION=HUMLIC Homo sapiens mRNA for lipocortin II, complete cds | 2 | Y |
| 34 | 34840_at |  | Cluster Incl. AI700633:we38g03.x1 Homo sapiens cDNA, 3 end /clone=IMAGE-2343412 /clone_end=3 /gb=AI700633 /gi=4988533 /ug=Hs.4815 /len=565 | 2 |  |
| 35 | 575_s_at | EPCAM | M93036 /FEATURE=mRNA /DEFINITION=HUMGA7A08 Human (clone 21726) carcinoma-associated antigen GA733-2 (GA733-2) mRNA, exon 9 and complete cds | 2 |  |
| 36 | 36918_at | GUCY1A3 | Cluster Incl. Y15723:Homo sapiens mRNA for soluble guanylyl cyclase /cds=(523,2595) /gb=Y15723 /gi=3702146 /ug=Hs.75295 /len=2982 | 2 |  |
| 37 | 39755_at | LOC646408 | Cluster Incl. Z93930:Human DNA sequence from clone 292E10 on chromosome 22q11-12. Contains the XBP1 gene for X-box binding protein 1 (TREB5), ESTs, STSs, GSSs and a putative CpG island /cds=(30,815) /gb=Z93930 /gi=4775603 /ug=Hs.149923 /len=1802 | 2 |  |
| 38 | 38814_at | ATP6V1G1 | Cluster Incl. AF038954:Homo sapiens vacuolar H(+)-ATPase subunit mRNA, complete cds /cds=(63,419) /gb=AF038954 /gi=3329377 /ug=Hs.90336 /len=1048 | 2 |  |
| 39 | 38827_at | AGR2 | Cluster Incl. AF038451:Homo sapiens secreted cement gland protein XAG-2 homolog (hAG-2/R) mRNA, complete cds /cds=(58,585) /gb=AF038451 /gi=3779225 /ug=Hs.91011 /len=1059 | 2 |  |
| 40 | 32076_at | RCAN2 | Cluster Incl. D83407:ZAKI-4 mRNA in human skin fibroblast, complete cds /cds=(204,782) /gb=D83407 /gi=1435039 /ug=Hs.156007 /len=3184 | 2 |  |
| 41 | 1521_at | NME1 | X17620 /FEATURE=mRNA /DEFINITION=HSNM23 Human mRNA for Nm23 protein, involved in developmental regulation (homolog. to Drosophila Awd protein) | 2 | Y |
| 42 | 1740_g_at | FOLH1 | M99487 /FEATURE= /DEFINITION=HUMPSM Human prostate-specific membrane antigen (PSM) mRNA, complete cds | 2 |  |
| 43 | 33904_at | CLDN3 | Cluster Incl. AB000714:Homo sapiens hRVP1 mRNA for RVP1, complete cds /cds=(198,860) /gb=AB000714 /gi=2570128 /ug=Hs.25640 /len=1250 | 2 |  |
| 44 | 34304_s_at | SAT1 | Cluster Incl. AL050290:Homo sapiens mRNA; cDNA DKFZp586G1923 (from clone DKFZp586G1923) /cds=(490,780) /gb=AL050290 /gi=4886512 /ug=Hs.28491 /len=1133 | 2 |  |
| 45 | 291_s_at | TACSTD2 | J04152 /FEATURE=mRNA /DEFINITION=HUMGA733A Human gastrointestinal tumor-associated antigen GA733-1 protein gene, complete cds, clone 05516 | 2 | Y |
| 46 | 31583_at | SNORD38B | Cluster Incl. X67247:H.sapiens rpS8 gene for ribosomal protein S8 /cds=(23,649) /gb=X67247 /gi=36149 /ug=Hs.118690 /len=705 | 2 |  |
| 47 | 41242_at | UAP1 | Cluster Incl. AB011004:Homo sapiens HuUAP1 mRNA for UDP-N-acetylglucosamine pyrophosphorylase, complete cds /cds=(0,1517) /gb=AB011004 /gi=3273315 /ug=Hs.21293 /len=1518 | 2 |  |
| 48 | 41485_at | LDHA | Cluster Incl. X02152:Human mRNA for lactate dehydrogenase-A (LDH-A, EC 1.1.1.27) /cds=(97,1095) /gb=X02152 /gi=34312 /ug=Hs.2795 /len=1661 | 2 |  |
| 49 | 41454_at | HEBP2 | Cluster Incl. W27949:39h3 Homo sapiens cDNA /gb=W27949 /gi=1307897 /ug=Hs.111029 /len=735 | 2 |  |
| 50 | 37141_at | FOXA1 | Cluster Incl. U39840:Human hepatocyte nuclear factor-3 alpha (HNF-3 alpha) mRNA, complete cds /cds=(87,1508) /gb=U39840 /gi=1066121 /ug=Hs.105440 /len=2872 | 2 | Y |

# 4 Pathway analysis of the genes selected by HBSA-SVM

Each gene with its occurrence frequency of at least one time is selected and analyzed in terms of its biological pathways on the website <http://vortex.cs.wayne.edu/projects.htm. The Tables S11-S16> are the results of the most significant pathways involved in the selected genes. For example, in the top-ranked pathway of cell adhesion molecules of SRBCT in Table S11, there are total 133 genes. In the DNA chip for SRBCT classification, there are 11 genes among them involved. With our method, five genes have the occurrence frequency of at least one time. The *p*-value of this pathway is 2.265E-4.In Table S14, the B-cell antigen receptor (BCR) of leukemia dataset is important for the survival of chronic lymphocytic leukemia cells.The experimental results demonstrate that the overexpressed active protein kinase C βplays a role in the regulation of BCR signal pathway that is important for the progression of CLL [86].

We find that the abnormality of these pathways is involved in uncontrolled cell proliferation (such as cell cycle,DNA replication [83]), carcinogenesis (base excision repair,mismatch repair,adipocytokine signaling pathway [99], etc), angiogenesis (like VEGF signaling pathway), metastasis (such as the pathway of cell adhesion molecules [84]),tumor suppressor pathway (such as p53 signaling pathway [85]), immunity escape (like pathways of antigen processing and presentation,B cell receptor signaling pathway,primary immunodeficiency, etc) or progression of a specific cancer or more than one kinds of cancers.

**Table S11**Ten pathways with the smallest *p*-values in the SRBCT dataset

| No. | Pathways | Pathway Genes  in the Chip | Input Genes  in the Chip | Pathway  Genes | *p*-Values |
| --- | --- | --- | --- | --- | --- |
| 1 | Cell adhesion molecules (CAMs)* | 11 | 5 | 133 | 2.265E-4 |
| 2 | Adherens junction | 24 | 7 | 75 | 3.077E-4 |
| 3 | Type I diabetes mellitus | 4 | 3 | 44 | 7.652E-4 |
| 4 | Asthma | 2 | 2 | 30 | 3.449E-3 |
| 5 | Antigen processing and presentation | 13 | 4 | 88 | 5.413E-3 |
| 6 | Endometrial cancer | 23 | 5 | 52 | 9.320E-3 |
| 7 | Autoimmune thyroid disease | 3 | 2 | 53 | 9.947E-3 |
| 8 | Graft-versus-host disease | 3 | 2 | 42 | 9.947E-3 |
| 9 | Allograft rejection | 3 | 2 | 38 | 9.947E-3 |
| 10 | Bladder cancer | 17 | 4 | 42 | 1.500E-2 |

*In the pathway of cell adhesion molecules, there are 133 genes in all. In the DNA chip for SRBCT classification, there are 11 genes among them involved. With our method, 5 genes have the occurrence frequency of at least one time. The p-value of this pathway is 2.265E-4.

**Table S12**Ten pathways with the smallest *p*-values in the ALL dataset

| No. | Pathways | Pathway Genes  in the Chip | Input Genes  in the Chip | Pathway  Genes | *p*-Values |
| --- | --- | --- | --- | --- | --- |
| 1 | Primary immunodeficiency | 32 | 4 | 35 | 3.162E-4 |
| 2 | Graft-versus-host disease | 32 | 4 | 42 | 3.162E-4 |
| 3 | Type I diabetes mellitus | 39 | 4 | 44 | 6.842E-4 |
| 4 | Hematopoietic cell lineage | 83 | 5 | 88 | 1.679E-3 |
| 5 | Cell adhesion molecules (CAMs) | 104 | 5 | 133 | 4.482E-3 |
| 6 | Allograft rejection | 33 | 3 | 38 | 4.749E-3 |
| 7 | Autoimmune thyroid disease | 44 | 3 | 53 | 1.062E-2 |
| 8 | Asthma | 27 | 2 | 30 | 3.176E-2 |
| 9 | Antigen processing and presentation | 70 | 3 | 88 | 3.620E-2 |
| 10 | Axon guidance | 101 | 3 | 128 | 8.790E-2 |

**Table S13**Ten pathways with the smallest *p*-Values in the colon tumor dataset

| No. | Pathways | Pathway Genes  in the Chip | Input Genes  in the Chip | Pathway  Genes | *p*-Values |
| --- | --- | --- | --- | --- | --- |
| 1 | Proteasome | 14 | 4 | 22 | 3.670E-2 |
| 2 | Base excision repair | 6 | 2 | 33 | 1.043E-1 |
| 3 | Ribosome | 27 | 5 | 91 | 1.055E-1 |
| 4 | $hsa05131$ | 20 | 3 | 51 | 2.944E-1 |
| 5 | Pathogenic Escherichia coli infection | 20 | 3 | 51 | 2.944E-1 |
| 6 | ABC transporters | 4 | 1 | 44 | 3.296E-1 |
| 7 | RNA polymerase | 4 | 1 | 25 | 3.296E-1 |
| 8 | Bladder cancer | 14 | 2 | 42 | 3.901E-1 |
| 9 | Cell cycle | 24 | 3 | 112 | 4.029E-1 |
| 10 | Hematopoietic cell lineage | 16 | 2 | 88 | 4.585E-1 |

**Table S14**Ten pathways with the smallest *p*-values in the leukemia dataset

| No. | Pathways | Pathway Genes  in the Chip | Input Genes  in the Chip | Pathway  Genes | *p*-Values |
| --- | --- | --- | --- | --- | --- |
| 1 | B cell receptor signaling pathway* | 48 | 5 | 64 | 4.396E-3 |
| 2 | VEGF signaling pathway | 50 | 4 | 71 | 2.624E-2 |
| 3 | Hematopoietic cell lineage | 82 | 5 | 88 | 3.814E-2 |
| 4 | Cytokine-cytokine receptor pathway | 184 | 8 | 259 | 5.748E-2 |
| 5 | Axon guidance | 67 | 4 | 128 | 6.529E-2 |
| 6 | T cell receptor signaling pathway | 68 | 4 | 93 | 6.820E-2 |
| 7 | Basal transcription factors | 19 | 2 | 34 | 6.820E-2 |
| 8 | Base excision repair | 19 | 2 | 33 | 6.820E-2 |
| 9 | Leukocyte transendothelial migration | 72 | 4 | 116 | 8.051E-2 |
| 10 | Mismatch repair | 21 | 2 | 22 | 8.135E-2 |

** Signals through the B-cell antigen receptor (BCR) are important for the survival of chronic lymphocytic leukemia cells, and the experimental results demonstrate that the overexpressed active protein kinase C βplays a role in the regulation and outcome of signals that can be important for the progression of CLL [86].*

**Table S15**Ten pathways with the smallest *p*-values in the DLBCL dataset

| No. | Pathways | Pathway Genes  in the Chip | Input Genes  in the Chip | Pathway  Genes | *p*-Values |
| --- | --- | --- | --- | --- | --- |
| 1 | Proteasome | 19 | 6 | 22 | 9.259E-5 |
| 2 | Cell adhesion molecules (CAMs) | 59 | 7 | 133 | 1.171E-2 |
| 3 | DNA replication | 24 | 4 | 35 | 1.719E-2 |
| 4 | Type I diabetes mellitus | 26 | 4 | 44 | 2.265E-2 |
| 5 | Cell cycle | 71 | 7 | 112 | 2.988E-2 |
| 6 | Antigen processing and presentation | 46 | 5 | 88 | 4.389E-2 |
| 7 | Leukocyte transendothelial migration | 63 | 6 | 116 | 4.960E-2 |
| 8 | Allograft rejection | 21 | 3 | 38 | 5.716E-2 |
| 9 | Renin-angiotensin system | 10 | 2 | 17 | 6.454E-2 |
| 10 | Vibrio cholerae infection | 26 | 3 | 59 | 9.594E-2 |

**Table S16**Ten pathways with the smallest *p*-values in the prostate dataset

| No. | Pathways | Pathway Genes  in the Chip | Input Genes  in the Chip | Pathway  Genes | *p*-Values |
| --- | --- | --- | --- | --- | --- |
| 1 | Ribosome23 | 60 | 21 | 91 | 1.571E-12 |
| 2 | p53 signaling pathway | 53 | 3 | 68 | 7.644E-2 |
| 3 | Adipocytokine signaling pathway | 63 | 3 | 72 | 1.136E-1 |
| 4 | Nucleotide excision repair | 39 | 2 | 43 | 1.650E-1 |
| 5 | Insulin signaling pathway | 123 | 4 | 138 | 1.985E-1 |
| 6 | Small cell lung cancer | 83 | 3 | 87 | 2.028E-1 |
| 7 | Cell cycle | 100 | 3 | 112 | 2.878E-1 |
| 8 | Biosynthesis of unsaturated fatty acids | 18 | 1 | 23 | 2.884E-1 |
| 9 | Cell adhesion molecules (CAMs) | 104 | 3 | 133 | 3.083E-1 |
| 10 | Antigen processing and presentation | 70 | 2 | 88 | 3.780E-1 |

# 5 Top-ranked genes selected by HBSA-KNN

For six tumor datasets, the Tables S17-S22 show the description of 50 top-ranked genes selected by the HBSA-KNN method and ranked by their occurrence frequencies in descending order, respectively, in which Column Frequency denotes the accumulated frequency of each gene in five runs of the HBSA-KNN. We also downloaded a set of known cancer genes from the website (http://cbio.mskcc.org/cancergenes) as of August 2009. 1086 known cancer genes are collected by querying the website for “oncogene”, “tumor suppressor” and “stability”. The known cancer genes comprise 338 oncogenes, 313 stability genes and 435 tumor suppressor genes. Overlap exists between the three kinds of cancer genes. In Tables S17-S22, column “Is cancer gene?” denotes whether the corresponding gene selected belongs to the known cancer genes or not.

**Table S17**Description of 50 top-ranked genes for the SRBCT dataset

| No. | Probe No. | Gene symbol | Description | Frequency | Is cancer gene? |
| --- | --- | --- | --- | --- | --- |
| 1 | 1435862 | CD99 | antigen identified by monoclonal antibodies 12E7, F21 and O13 | 825 |  |
| 2 | 812105 | MLLT11 | Transmembrane protein | 759 |  |
| 3 | 207274 | IGF2 | Human DNA for insulin-like growth factor II (IGF-2); exon 7 and additional ORF | 618 |  |
| 4 | 377461 | CAV1 | caveolin 1, caveolae protein, 22kD | 449 | Y |
| 5 | 143306 | Lsp1 | lymphocyte-specific protein 1 | 322 |  |
| 6 | 769716 | NF2 | neurofibromin 2 (bilateral acoustic neuroma) | 277 | Y |
| 7 | 770394 | FCGRT | Fc fragment of IgG, receptor, transporter, alpha | 247 |  |
| 8 | 325182 | CDH2 | cadherin 2, N-cadherin (neuronal) | 195 |  |
| 9 | 629896 | MAP1B | microtubule-associated protein 1B | 152 |  |
| 10 | 241412 | ELF1 | E74-like factor 1 (ets domain transcription factor) | 103 |  |
| 11 | 308231 | MYO1B | Homo sapiens incomplete cDNA for a mutated allele of a myosin class I, myh-1c | 83 |  |
| 12 | 784224 | FGFR4 | fibroblast growth factor receptor 4 | 81 |  |
| 13 | 563673 | ALDH7A1 | antiquitin 1 | 80 |  |
| 14 | 767495 | Gli3 | GLI-Kruppel family member GLI3 (Greig cephalopolysyndactyly syndrome) | 79 |  |
| 15 | 81518 | OCRL | apelin; peptide ligand for APJ receptor | 77 |  |
| 16 | 244618 |  | ESTs | 55 |  |
| 17 | 183337 | HLA-DMB | major histocompatibility complex, class II, DM alpha | 44 |  |
| 18 | 796258 | SGCA | sarcoglycan, alpha (50kD dystrophin-associated glycoprotein) | 44 | Y |
| 19 | 627939 | CSRP3 | cysteine and glycine-rich protein 3 (cardiac LIM protein) | 42 | Y |
| 20 | 782193 | TXN | Thioredoxin | 34 |  |
| 21 | 878652 | PMS2L12 | postmeiotic segregation increased 2-like 12 | 33 |  |
| 22 | 52076 | OLFM1 | olfactomedinrelated ER localized protein | 31 |  |
| 23 | 814260 | KDSR | follicular lymphoma variant translocation 1 | 29 |  |
| 24 | 134748 | GCSH | glycine cleavage system protein H (aminomethyl carrier) | 26 |  |
| 25 | 207358 | SLC2A1 | solute carrier family 2 (facilitated glucose transporter), member 1 | 26 |  |
| 26 | 204299 | RPA3 | replication protein A3 (14kD) | 24 |  |
| 27 | 866702 | PTPN13 | protein tyrosine phosphatase, non-receptor type 13 (APO-1/CD95 (Fas)-associated phosphatase) | 24 |  |
| 28 | 789091 | HIST1H2AC | H2A histone family, member L | 24 |  |
| 29 | 898219 | MEST | mesoderm specific transcript (mouse) homolog | 24 |  |
| 30 | 729964 | PSAP | sphingomyelin phosphodiesterase 1, acid lysosomal (acid sphingomyelinase) | 23 |  |
| 31 | 813742 | PTK7 | PTK7 protein tyrosine kinase 7 | 22 |  |
| 32 | 842918 | FARP1 | chondrocyte-derived ezrin-like protein | 21 |  |
| 33 | 841641 | CCND1 | cyclin D1 (PRAD1: parathyroid adenomatosis 1) | 21 | Y |
| 34 | 25725 | FDFT1 | farnesyl-diphosphate farnesyltransferase 1 | 21 |  |
| 35 | 80338 | SELENBP1 | selenium binding protein 1 | 21 |  |
| 36 | 377731 | GSTM5 | glutathione S-transferase M5 | 19 |  |
| 37 | 245330 | ZBTB48 | Human Krueppel-related zinc finger protein (H-plk) mRNA, complete cds | 19 |  |
| 38 | 383188 | RCVRN | Recoverin | 18 |  |
| 39 | 784257 | KIF3C | kinesin family member 3C | 18 |  |
| 40 | 1470048 | LY6G6E | lymphocyte antigen 6 complex, locus E | 17 |  |
| 41 | 859359 | TP53I3 | quinone oxidoreductase homolog | 16 | Y |
| 42 | 236282 | WAS | Wiskott-Aldrich syndrome (ecezema-thrombocytopenia) | 16 |  |
| 43 | 43733 | GYG2 | glycogenin 2 | 15 |  |
| 44 | 878280 | CRMP1 | collapsin response mediator protein 1 | 15 |  |
| 45 | 841620 | DPYSL2 | dihydropyrimidinase-like 2 | 15 |  |
| 46 | 784593 |  | ESTs | 15 |  |
| 47 | 234237 | PIR | Pirin | 15 |  |
| 48 | 530185 | CD83 | CD83 antigen (activated B lymphocytes, immunoglobulin superfamily) | 15 |  |
| 49 | 897177 | PGAM1 | phosphoglycerate mutase 1 (brain) | 15 |  |
| 50 | 377048 | MYO1B | Homo sapiens incomplete cDNA for a mutated allele of a myosin class I, myh-1c | 14 |  |

**Table S18**Description of 50 top-ranked genes for the ALL dataset

| No. | Probe No. | Gene symbol | Description | Frequency | Is cancer gene? |
| --- | --- | --- | --- | --- | --- |
| 1 | 36985_at | IDI1 | Cluster Incl. X17025:Human homolog of yeast IPP isomerase /cds=(50,736) /gb=X17025 /gi=488749 /ug=Hs.76038 /len=1807 | 1494 |  |
| 2 | 38242_at | BLNK | Cluster Incl. AF068180:Homo sapiens B cell linker protein BLNK mRNA, alternatively spliced, complete cds /cds=(153,1523) /gb=AF068180 /gi=3406748 /ug=Hs.167746 /len=1790 | 1113 |  |
| 3 | 32207_at | MPP1 | Cluster Incl. M64925:Human palmitoylated erythrocyte membrane protein (MPP1) mRNA, complete cds /cds=(103,1503) /gb=M64925 /gi=189785 /ug=Hs.1861 /len=1989 | 804 |  |
| 4 | 37470_at | LAIR1 | Cluster Incl. AF013249:Homo sapiens leukocyte-associated Ig-like receptor-1 (LAIR-1) mRNA, complete cds /cds=(68,931) /gb=AF013249 /gi=2352940 /ug=Hs.115808 /len=1675 | 689 |  |
| 5 | 1287_at | PARP1 | J03473 /FEATURE=mRNA /DEFINITION=HUMRISDAD Human poly(ADP-ribose) synthetase mRNA, complete cds | 638 | Y |
| 6 | 38518_at | SCML2 | Cluster Incl. Y18004:Homo sapiens mRNA for SCML2 protein /cds=(91,2193) /gb=Y18004 /gi=4490941 /ug=Hs.171558 /len=4130 | 571 |  |
| 7 | 35974_at | LRMP | Cluster Incl. U10485:Human lymphoid-restricted membrane protein (Jaw1) mRNA, complete cds /cds=(574,2241) /gb=U10485 /gi=505685 /ug=Hs.40202 /len=2417 | 547 |  |
| 8 | 33821_at |  | Cluster Incl. AL034374:Human DNA sequence from clone 483K16 on chromosome 6p12.1-21.1. Contains (parts of) two novel genes, 40S Ribosomal protein S16 and 60S Ribosomal protein L31 pseudogenes, ESTs, STSs, GSSs and a putative CpG island /cds=(0,703) /gb=AL034374 /gi=4455565 /ug=Hs.234555 /len=2432 | 295 |  |
| 9 | 34168_at | DNTT | Cluster Incl. M11722:Human terminal transferase mRNA, complete cds /cds=(328,1854) /gb=M11722 /gi=339436 /ug=Hs.234772 /len=2068 | 239 |  |
| 10 | 39003_at | PTTG1IP | Cluster Incl. Z50022:H.sapiens mRNA for surface glycoprotein /cds=(93,635) /gb=Z50022 /gi=1107702 /ug=Hs.111126 /len=2617 | 210 |  |
| 11 | 37343_at | ITPR3 | Cluster Incl. U01062:Human type 3 inositol 1,4,5-trisphosphate receptor (ITPR3) mRNA, complete cds /cds=(36,8051) /gb=U01062 /gi=453367 /ug=Hs.77515 /len=8833 | 200 |  |
| 12 | 37039_at | HLA-DRA | Cluster Incl. J00194:human hla-dr antigen alpha-chain mrna & ivs fragments /cds=(26,790) /gb=J00194 /gi=188231 /ug=Hs.76807 /len=1199 | 185 |  |
| 13 | 38408_at | TSPAN7 | Cluster Incl. L10373:Human (clone CCG-B7) mRNA sequence /cds=UNKNOWN /gb=L10373 /gi=307287 /ug=Hs.82749 /len=1792 | 162 |  |
| 14 | 35648_at | AUTS2 | Cluster Incl. AB007902:Homo sapiens KIAA0442 mRNA, partial cds /cds=(0,3519) /gb=AB007902 /gi=2662164 /ug=Hs.32168 /len=5379 | 129 |  |
| 15 | 36239_at | POU2AF1 | Cluster Incl. Z49194:H.sapiens mRNA for oct-binding factor /cds=(523,1293) /gb=Z49194 /gi=974830 /ug=Hs.2407 /len=3301 | 112 |  |
| 16 | 40518_at | PTPRC | Cluster Incl. Y00062:Human mRNA for T200 leukocyte common antigen (CD45, LC-A) /cds=(146,3577) /gb=Y00062 /gi=34275 /ug=Hs.170121 /len=4597 | 105 |  |
| 17 | 39168_at | DHRSX | Cluster Incl. AB018328:Homo sapiens mRNA for KIAA0785 protein, complete cds /cds=(201,2285) /gb=AB018328 /gi=3882290 /ug=Hs.9933 /len=4485 | 92 |  |
| 18 | 33121_g_at | RGS10 | Cluster Incl. AF045229:Homo sapiens regulator of G protein signaling 10 mRNA, complete cds /cds=(132,635) /gb=AF045229 /gi=2906029 /ug=Hs.82280 /len=753 | 89 |  |
| 19 | 40522_at | GLUL | Cluster Incl. X59834:Human rearranged mRNA for glutamine synthase /cds=(109,1230) /gb=X59834 /gi=31830 /ug=Hs.170171 /len=2715 | 86 |  |
| 20 | 39827_at | DDIT4 | Cluster Incl. AA522530:ni38d12.s1 Homo sapiens cDNA, 3 end /clone=IMAGE-979127 /clone_end=3 /gb=AA522530 /gi=2263242 /ug=Hs.111244 /len=891 | 81 |  |
| 21 | 914_g_at | ERG | M21535 /FEATURE= /DEFINITION=HUMERG11 Human erg protein (ets-related gene) mRNA, complete cds | 79 | Y |
| 22 | 39114_at | C10orf10 | Cluster Incl. AB022718:Homo sapiens mRNA for DEPP (decidual protein induced by progesterone), complete cds /cds=(218,856) /gb=AB022718 /gi=4204189 /ug=Hs.93675 /len=2114 | 78 |  |
| 23 | 37780_at | PCLO | Cluster Incl. AB011131:Homo sapiens mRNA for KIAA0559 protein, partial cds /cds=(0,3640) /gb=AB011131 /gi=3043641 /ug=Hs.12376 /len=5639 | 70 |  |
| 24 | 35614_at | TCFL5 | Cluster Incl. AB012124:Homo sapiens TCFL5 mRNA for transcription factor-like 5, complete cds /cds=(98,1456) /gb=AB012124 /gi=4126408 /ug=Hs.30696 /len=2316 | 69 |  |
| 25 | 2031_s_at | CDKN1A | U03106 /FEATURE= /DEFINITION=HSU03106 Human wild-type p53 activated fragment-1 (WAF1) mRNA, complete cds | 63 | Y |
| 26 | 1105_s_at | **IL23A** | M12886 /FEATURE= /DEFINITION=HUMTCBYY Human T-cell receptor active beta-chain mRNA, complete cds | 63 |  |
| 27 | 32794_g_at | **IL23A** | Cluster Incl. X00437:Human mRNA for T-cell specific protein /cds=(37,975) /gb=X00437 /gi=36748 /ug=Hs.2003 /len=1151 | 59 |  |
| 28 | 38994_at | SOCS2 | Cluster Incl. AF037989:Homo sapiens STAT-induced STAT inhibitor-2 mRNA, complete cds /cds=(317,913) /gb=AF037989 /gi=3265032 /ug=Hs.110776 /len=1937 | 53 |  |
| 29 | 430_at | NP | X00737 /FEATURE=cds /DEFINITION=HSPNP Human mRNA for purine nucleoside phosphorylase (PNP; EC 2.4.2.1) | 53 |  |
| 30 | 41442_at | CBFA2T3 | Cluster Incl. AB010419:Homo sapiens mRNA for MTG8-related protein MTG16a, complete cds /cds=(158,2119) /gb=AB010419 /gi=3256263 /ug=Hs.110099 /len=4221 | 52 | Y |
| 31 | 307_at | ALOX5 | J03600 /FEATURE= /DEFINITION=HUMLOX5 Human lipoxygenase mRNA, complete cds | 49 |  |
| 32 | 37416_at | RHOH | Cluster Incl. Z35227:H.sapiens TTF mRNA for small G protein /cds=(579,1154) /gb=Z35227 /gi=609016 /ug=Hs.109918 /len=1427 | 44 |  |
| 33 | 32174_at | SLC9A3R1 | Cluster Incl. AF015926:Homo sapiens ezrin-radixin-moesin binding phosphoprotein-50 mRNA, complete cds /cds=(212,1288) /gb=AF015926 /gi=3220018 /ug=Hs.184276 /len=1984 | 43 | Y |
| 34 | 38578_at | CD27 | Cluster Incl. M63928:Homo sapiens T cell activation antigen (CD27) mRNA, complete cds /cds=(100,882) /gb=M63928 /gi=180084 /ug=Hs.180841 /len=1204 | 43 |  |
| 35 | 37543_at | ARHGEF6 | Cluster Incl. D25304:Human mRNA for KIAA0006 gene, partial cds /cds=(0,2323) /gb=D25304 /gi=435445 /ug=Hs.79307 /len=4804 | 42 |  |
| 36 | 33819_at | LDHB | Cluster Incl. X13794:H.sapiens lactate dehydrogenase B gene exon 1 and 2 (EC 1.1.1.27) (and joined CDS) /cds=(84,1088) /gb=X13794 /gi=34314 /ug=Hs.234489 /len=1272 | 41 |  |
| 37 | 41425_at | FLI1 | Cluster Incl. M98833:Human ERGB transcription factor (FLI-1 homolog) mRNA, complete cds /cds=(172,1527) /gb=M98833 /gi=182188 /ug=Hs.108043 /len=2954 | 40 | Y |
| 38 | 2047_s_at | JUP | M23410 /FEATURE= /DEFINITION=HUMPLAKO Human plakoglobin (PLAK) mRNA, complete cds | 39 | Y |
| 39 | 36383_at | ERG | Cluster Incl. M17254:Human erg2 gene encoding erg2 protein, complete cds /cds=(0,1388) /gb=M17254 /gi=182186 /ug=Hs.159432 /len=1389 | 38 | Y |
| 40 | 40745_at | AP1B1 | Cluster Incl. L13939:Homo sapiens beta adaptin (BAM22) mRNA, complete cds /cds=(46,2895) /gb=L13939 /gi=4079593 /ug=Hs.89576 /len=3859 | 36 |  |
| 41 | 32979_at | GAB1 | Cluster Incl. U43885:Human Grb2-associated binder-1 mRNA, complete cds /cds=(121,2205) /gb=U43885 /gi=1199617 /ug=Hs.239706 /len=2467 | 36 |  |
| 42 | 34194_at | CLIC5 | Cluster Incl. AL049313:Homo sapiens mRNA; cDNA DKFZp564B076 (from clone DKFZp564B076) /cds=UNKNOWN /gb=AL049313 /gi=4500086 /ug=Hs.21103 /len=2190 | 36 |  |
| 43 | 39829_at | ARL4C | Cluster Incl. AB016811:Homo sapiens mRNA for ADP ribosylation factor-like protein, complete cds /cds=(22,549) /gb=AB016811 /gi=4514625 /ug=Hs.111554 /len=1397 | 35 |  |
| 44 | 38124_at | MDK | Cluster Incl. X55110:Human mRNA for neurite outgrowth-promoting protein /cds=(25,456) /gb=X55110 /gi=35086 /ug=Hs.82045 /len=786 | 33 |  |
| 45 | 39755_at | XBP1 | Cluster Incl. Z93930:Human DNA sequence from clone 292E10 on chromosome 22q11-12. Contains the XBP1 gene for X-box binding protein 1 (TREB5), ESTs, STSs, GSSs and a putative CpG island /cds=(30,815) /gb=Z93930 /gi=4775603 /ug=Hs.149923 /len=1802 | 32 |  |
| 46 | 41213_at | PRDX1 | Cluster Incl. X67951:H.sapiens mRNA for proliferation-associated gene (pag) /cds=(60,659) /gb=X67951 /gi=287640 /ug=Hs.180909 /len=937 | 30 |  |
| 47 | 34780_at | PLXNB2 | Cluster Incl. AB002313:Human mRNA for KIAA0315 gene, partial cds /cds=(0,5526) /gb=AB002313 /gi=2280475 /ug=Hs.3989 /len=6252 | 30 |  |
| 48 | 577_at | MDK | M94250 /FEATURE=expanded_cds /DEFINITION=HUMMKXX Human retinoic acid inducible factor (MK) gene exons 1-5, complete cds | 30 |  |
| 49 | 32035_at | HLA-DRB1 | Cluster Incl. M16942:Human MHC class II HLA-DRw53-associated glycoprotein beta- chain mRNA, complete cds /cds=(28,828) /gb=M16942 /gi=188352 /ug=Hs.155122 /len=1141 | 29 |  |
| 50 | 41200_at | SCARB1 | Cluster Incl. Z22555:H.sapiens encoding CLA-1 mRNA /cds=(69,1598) /gb=Z22555 /gi=397606 /ug=Hs.180616 /len=2552 | 29 |  |

**Table S19** Description of 50 top-ranked genes for the colon tumor dataset

| No. | Access No. | Gene symbol | Description | Frequency | Is cancer gene? |
| --- | --- | --- | --- | --- | --- |
| 1 | M80815 | FUCA1 | H.sapiens a-L-fucosidase gene, exon 7 and 8, and complete cds. | 246 |  |
| 2 | R87126 | MYO5A | MYOSIN HEAVY CHAIN, NONMUSCLE (Gallus gallus) | 218 |  |
| 3 | J05032 | DARS | Human aspartyl-tRNA synthetase alpha-2 subunit mRNA, complete cds. | 151 |  |
| 4 | H77597 | MT2A | H.sapiens mRNA for metallothionein (HUMAN); | 129 |  |
| 5 | M26383 | IL8 | Human monocyte-derived neutrophil-activating protein (MONAP) mRNA, complete cds. | 113 |  |
| 6 | M22382 | FXN | MITOCHONDRIAL MATRIX PROTEIN P1 PRECURSOR (HUMAN); | 89 |  |
| 7 | H43887 | C3 | COMPLEMENT FACTOR D PRECURSOR (Homo sapiens) | 79 |  |
| 8 | M36634 | VIP | Human vasoactive intestinal peptide (VIP) mRNA, complete cds. | 78 |  |
| 9 | H64489 | CD37 | LEUKOCYTE ANTIGEN CD37 (Homo sapiens); | 66 |  |
| 10 | X54942 | CKS2 | H.sapiens ckshs2 mRNA for Cks1 protein homologue. | 65 |  |
| 11 | D16294 | ACAA2 | Human mRNA for mitochondrial 3-oxoacyl-CoA thiolase, complete cds. | 55 |  |
| 12 | T92451 | TPM2 | TROPOMYOSIN, FIBROBLAST AND EPITHELIAL MUSCLE-TYPE (HUMAN); | 54 |  |
| 13 | **M76378** | **CRIP2** | Human cysteine-rich protein (CRP) gene, exons 5 and 6. | 42 |  |
| 14 | **M76378** | **CRIP2** | Human cysteine-rich protein (CRP) gene, exons 5 and 6. | 42 |  |
| 15 | H20709 | MYL6 | MYOSIN LIGHT CHAIN ALKALI, SMOOTH-MUSCLE ISOFORM (HUMAN); | 41 |  |
| 16 | X14958 | HMGA1 | Human hmgI mRNA for high mobility group protein Y. | 37 | Y |
| 17 | R36977 | GTF3A | P03001 TRANSCRIPTION FACTOR IIIA; | 34 |  |
| 18 | T51571 | S100A11 | P24480 CALGIZZARIN. | 31 | Y |
| 19 | Z50753 | GUCA2B | H.sapiens mRNA for GCAP-II/uroguanylin precursor. | 30 |  |
| 20 | T51023 | HSP90AB1 | HEAT SHOCK PROTEIN HSP 90-BETA (HUMAN). | 30 |  |
| 21 | R59202 | MEF2A | MYOCYTE-SPECIFIC ENHANCER FACTOR 2, ISOFORM MEF2 (Homo sapiens) | 25 |  |
| 22 | J02854 | MYL6 | MYOSIN REGULATORY LIGHT CHAIN 2, SMOOTH MUSCLE ISOFORM (HUMAN);contains element TAR1 repetitive element; | 23 |  |
| 23 | H40095 | MIF | MACROPHAGE MIGRATION INHIBITORY FACTOR (HUMAN); | 23 | Y |
| 24 | H87135 | IE | IMMEDIATE-EARLY PROTEIN IE180 (Pseudorabies virus) | 23 |  |
| 25 | X63629 | CDH3 | H.sapiens mRNA for p cadherin. | 21 |  |
| 26 | D63874 | HMGB1 | Human mRNA for HMG-1. | 21 | Y |
| 27 | R44301 | NR3C2 | MINERALOCORTICOID RECEPTOR (Homo sapiens) | 20 |  |
| 28 | R33367 | CASK | MEMBRANE COFACTOR PROTEIN PRECURSOR (Homo sapiens) | 20 |  |
| 29 | L41559 | PCBD1 | Homo sapiens pterin-4a-carbinolamine dehydratase (PCBD) mRNA, complete cds. | 19 |  |
| 30 | T40454 | CD47 | ANTIGENIC SURFACE DETERMINANT PROTEIN OA3 PRECURSOR (Homo sapiens) | 19 |  |
| 31 | M26697 | NPM1 | Human nucleolar protein (B23) mRNA, complete cds. | 18 | Y |
| 32 | H08393 | COL11A2 | COLLAGEN ALPHA 2(XI) CHAIN (Homo sapiens) | 18 |  |
| 33 | X86693 | SPARCL1 | H.sapiens mRNA for hevin like protein. | 18 |  |
| 34 | T95018 |  | 40S RIBOSOMAL PROTEIN S18 (Homo sapiens) | 17 |  |
| 35 | D31885 | ARL6IP1 | Human mRNA (KIAA0069) for ORF (novel proetin), partial cds. | 16 |  |
| 36 | U04953 | RPS18 | Human isoleucyl-tRNA synthetase mRNA, complete cds. | 16 |  |
| 37 | D14812 | MORF4L2 | Human mRNA for ORF, complete cds. | 16 | Y |
| 38 | T51261 | App | GLIA DERIVED NEXIN PRECURSOR (Mus musculus) | 15 |  |
| 39 | T51493 |  | Homo sapiens PP2A B56-gamma1 mRNA, 3'' end of cds. | 15 |  |
| 40 | X12466 | SNRPE | Human mRNA for snRNP E protein. | 15 |  |
| 41 | M58050 | CD46 | Human membrane cofactor protein (MCP) mRNA, complete cds. | 15 |  |
| 42 | T60155 | ACTA2 | ACTIN, AORTIC SMOOTH MUSCLE (HUMAN); | 14 | Y |
| 43 | U21090 | POLD2 | Human DNA polymerase delta small subunit mRNA, complete cds. | 14 |  |
| 44 | T84049 | SET | SET PROTEIN (Homo sapiens) | 14 | Y |
| 45 | T86749 | CDK4 | Human (clone PSK-J3) cyclin-dependent protein kinase mRNA, complete cds. | 14 |  |
| 46 | R84411 | SNRPB | SMALL NUCLEAR RIBONUCLEOPROTEIN ASSOCIATED PROTEINS B AND B'' (HUMAN); | 13 |  |
| 47 | U07695 | EPHB4 | Human tyrosine kinase (HTK) mRNA, complete cds. | 13 | Y |
| 48 | T57619 | RPS6 | 40S RIBOSOMAL PROTEIN S6 (Nicotiana tabacum) | 13 |  |
| 49 | X16356 | BGPc | Human mRNA for transmembrane carcinoembryonic antigen BGPC (part.) (formerly TM3-CEA). | 13 |  |
| 50 | U32519 | ASAP2 | Human GAP SH3 binding protein mRNA, complete cds. | 13 |  |

**Table S20**Description of 50 top-ranked genes for the DLBCL dataset

| No. | Probe No. | Gene symbol | Description | Frequency | Is cancer gene? |
| --- | --- | --- | --- | --- | --- |
| 1 | Z35227_at | RHOH | TTF mRNA for small G protein | 631 |  |
| 2 | X02152_at | LDHA | LDHA Lactate dehydrogenase A | 374 |  |
| 3 | M94880_f_at | HLA-A | HLA-A MHC class I protein HLA-A (HLA-A28,-B40, -Cw3) | 333 |  |
| 4 | D83597_at | CD180 | RP105 | 168 |  |
| 5 | L42324_at | NCOR2 | (clone GPCR W) G protein-linked receptor gene (GPCR) gene, 5'' end of cds | 168 |  |
| 6 | L25876_at | CDKN3 | Protein tyrosine phosphatase (CIP2)mRNA | 135 |  |
| 7 | D55716_at | MCM7 | DNA REPLICATION LICENSING FACTOR CDC47 HOMOLOG | 132 |  |
| 8 | X17567_s_at | SNRPB | SNRPB Small nuclear ribonucleoprotein polypeptides B and B1 | 130 |  |
| 9 | HG2279-HT2375_at | TPI1 | Triosephosphate Isomerase | 95 |  |
| 10 | L02426_at | PSMC1 | 26S PROTEASE REGULATORY SUBUNIT 4 | 91 |  |
| 11 | M63138_at | CTSD | CTSD Cathepsin D (lysosomal aspartyl protease) | 80 |  |
| 12 | D78134_at | CIRBP | YWHAZ Tyrosine 3-monooxygenase/tryptophan 5-monooxygenase activation protein, zeta polypeptide | 76 |  |
| 13 | D87119_at | TRIB2 | Cancellous bone osteoblast mRNA for GS3955 | 72 |  |
| 14 | D38076_at | RANBP1 | RANBP1 RAN binding protein 1 | 68 |  |
| 15 | M63835_at | FCGR1A | HIGH AFFINITY IMMUNOGLOBULIN GAMMA FC RECEPTOR I "A FORM" PRECURSOR | 56 |  |
| 16 | X12447_at | ALDOA | ALDOA Aldolase A | 50 |  |
| 17 | X67951_at | PRDX1 | PAGA Proliferation-associated gene A (natural killer-enhancing factor A) | 47 |  |
| 18 | Z21966_at | POU6F1 | POU6F1 POU homeobox protein | 40 |  |
| 19 | M22760_at | COX5A | CYTOCHROME C OXIDASE POLYPEPTIDE VA PRECURSOR | 38 |  |
| 20 | U28386_at | KPNA2 | RCH1 RAG (recombination activating gene) cohort 1 | 38 | Y |
| 21 | U81375_at | SLC29A1 | Placental equilibrative nucleoside transporter 1 (hENT1) mRNA | 36 |  |
| 22 | X16983_at | ITGA4 | ITGA4 Integrin, alpha 4 (antigen CD49D, alpha 4 subunit of VLA-4 receptor) | 33 | Y |
| 23 | X56494_at | PKM2 | PKM2 Pyruvate kinase, muscle | 32 |  |
| 24 | U48296_at | PTP4A1 | Protein tyrosine phosphatase PTPCAAX1 (hPTPCAAX1) mRNA | 30 |  |
| 25 | L03411_s_at | RDBP | RD Radin blood group | 29 |  |
| 26 | V00594_at | MT2A | Metallothionein isoform 2 | 29 |  |
| 27 | M25753_at | CCNB1 | G2/MITOTIC-SPECIFIC CYCLIN B1 | 29 |  |
| 28 | HG2874-HT3018_at | MRPL39 | Ribosomal Protein L39 Homolog | 28 |  |
| 29 | Z49099_at | SMS | Spermine synthase | 25 |  |
| 30 | L19437_at | TALDO1 | TALDO Transaldolase | 24 |  |
| 31 | X15183_at | HSP90AA1 | 60S RIBOSOMAL PROTEIN L13 | 23 |  |
| 32 | M14328_s_at | ENO1 | ENO1 Enolase 1, (alpha) | 22 | Y |
| 33 | M35878_at | IGF2 | INSULIN-LIKE GROWTH FACTOR BINDING PROTEIN 3 PRECURSOR | 21 |  |
| 34 | D31887_at | SLC39A14 | KIAA0062 gene, partial cds | 21 |  |
| 35 | U53347_at | SLC1A5 | Neutral amino acid transporter B mRNA | 21 |  |
| 36 | U70660_at | ATOX1 | Copper transport protein HAH1 (HAH1) mRNA | 20 |  |
| 37 | U09587_at | GARS | GARS Glycyl-tRNA synthetase | 19 |  |
| 38 | U29680_at | BCL2A1 | Bcl-2 related (Bfl-1) mRNA | 19 | Y |
| 39 | U24169_at | JTV1 | JTV-1 (JTV-1) mRNA | 18 |  |
| 40 | U14518_at | CENPA | CENPA Centromere protein A (17kD) | 18 |  |
| 41 | D82348_at | ATIC | 5-aminoimidazole-4-carboxamide-1-beta-D-ribonucleoti de transformylase inosinicase | 17 |  |
| 42 | M63379_at | CLU | CLU Clusterin (complement lysis inhibitor; testosterone-repressed prostate message 2; apolipoprotein J) | 17 | Y |
| 43 | HG4258-HT4528_at | CDKN1B | Kinase Inhibitor P27kip1, Cyclin-Dependent | 17 | Y |
| 44 | S80343_at | RARS | RARS Arginyl-tRNA synthetase | 16 |  |
| 45 | J04173_at | PGAM1 | PGAM1 Phosphoglycerate mutase 1 (brain) | 16 |  |
| 46 | L07956_at | GBE1 | GBE1 Glucan (1,4-alpha-), branching enzyme 1 (glycogen branching enzyme, Andersen disease, glycogen storage disease type IV) | 16 |  |
| 47 | M20471_at | CLTA | CLTA Clathrin light chain A | 16 |  |
| 48 | X62078_at | GM2A | GM2A GM2 ganglioside activator protein | 15 |  |
| 49 | X76534_at | GPNMB | NMB Neuromedin B | 15 |  |
| 50 | HG1980-HT2023_at | TUBB | Tubulin, Beta 2 | 15 | Y |

**Table S21** Description of 50 top-ranked genes for the leukemia dataset

| No. | Probe No. | Gene symbol | Description | Frequency | Is cancer gene? |
| --- | --- | --- | --- | --- | --- |
| 1 | L09209_s_at | APLP2 | APLP2 Amyloid beta (A4) precursor-like protein 2 | 700 |  |
| 2 | M23197_at | CD33 | CD33 CD33 antigen (differentiation antigen) | 324 |  |
| 3 | X95735_at | ZYX | Zyxin | 264 | Y |
| 4 | HG1612-HT1612_at | MARCKSL1 | Macmarcks | 170 |  |
| 5 | X68560_at | SP3 | SP3 Sp3 transcription factor | 117 |  |
| 6 | X62654_rna1_at | CD63 | ME491 gene extracted from H.sapiens gene for Me491/CD63 antigen | 102 |  |
| 7 | D84294_at | TTC3 | TPRD | 95 |  |
| 8 | L07633_at | PSME1 | INTERFERON GAMMA UP-REGULATED I-5111 PROTEIN PRECURSOR | 86 |  |
| 9 | M92287_at | CCND3 | CCND3 Cyclin D3 | 84 | Y |
| 10 | M27891_at | CST3 | CST3 Cystatin C (amyloid angiopathy and cerebral hemorrhage) | 82 |  |
| 11 | M31523_at | TCF3 | TCF3 Transcription factor 3 (E2A immunoglobulin enhancer binding factors E12/E47) | 81 | Y |
| 12 | U05259_rna1_at | CD79A | MB-1 gene | 80 |  |
| 13 | U77948_at | GTF2I | KAI1 Kangai 1 (suppression of tumorigenicity 6, prostate; CD82 antigen (R2 leukocyte antigen, antigen detected by monoclonal and antibody IA4)) | 78 |  |
| 14 | X51521_at | EZR | VIL2 Villin 2 (ezrin) | 77 |  |
| 15 | M11722_at | DNTT | Terminal transferase mRNA | 75 |  |
| 16 | X56468_at | YWHAQ | 14-3-3 PROTEIN TAU | 73 | Y |
| 17 | Y07604_at | NME4 | Nucleoside-diphosphate kinase | 68 |  |
| 18 | U94855_at | EIF3F | Translation initiation factor 3 47 kDa subunit mRNA | 61 |  |
| 19 | X63753_at | SON | SON SON DNA binding protein | 58 |  |
| 20 | U90549_at | HMGN4 | Non-histone chromosomal protein (NHC) mRNA | 56 |  |
| 21 | J05243_at | SPTAN1 | SPTAN1 Spectrin, alpha, non-erythrocytic 1 (alpha-fodrin) | 54 |  |
| 22 | J03589_at | UBL4A | UBIQUITIN-LIKE PROTEIN GDX | 50 |  |
| 23 | U49020_cds2_s_at | MEF2A | MEF2A gene (myocyte-specific enhancer factor 2A, C9 form) extracted from Human myocyte-specific enhancer factor 2A (MEF2A) gene, first coding | 49 |  |
| 24 | M63138_at | CTSD | CTSD Cathepsin D (lysosomal aspartyl protease) | 48 |  |
| 25 | U72936_s_at | ATRX | X-LINKED HELICASE II | 47 | Y |
| 26 | D42043_at | RFTN1 | KIAA0084 gene, partial cds | 43 |  |
| 27 | U62136_at | UBE2V2 | Putative enterocyte differentiation promoting factor mRNA, partial cds | 38 | Y |
| 28 | M60527_at | DCK | DCK Deoxycytidine kinase | 32 |  |
| 29 | U27460_at | UGP2 | Uridine diphosphoglucose pyrophosphorylase mRNA | 30 |  |
| 30 | X69111_at | ID3 | ID3 Inhibitor of DNA binding 3, dominant negative helix-loop-helix protein | 29 |  |
| 31 | M91432_at | ACADM | ACADM Acyl-Coenzyme A dehydrogenase, C-4 to C-12 straight chain | 27 |  |
| 32 | D26156_s_at | SMARCA4 | Transcriptional activator hSNF2b | 25 | Y |
| 33 | U16954_at | MLLT11 | (AF1q) mRNA | 24 |  |
| 34 | M96803_at | SPTBN1 | SPTBN1 Spectrin, beta, non-erythrocytic 1 | 23 |  |
| 35 | U77604_at | MGST2 | Microsomal glutathione S-transferase (GST-II) mRNA | 22 |  |
| 36 | X97267_rna1_s_at | PTPRCAP | LPAP gene | 22 |  |
| 37 | L20010_at | HCFC1 | HCF1 gene related mRNA sequence | 22 |  |
| 38 | M89957_at | CD79B | IGB Immunoglobulin-associated beta (B29) | 22 |  |
| 39 | M29696_at | IL7R | IL7R Interleukin 7 receptor | 21 |  |
| 40 | U22376_cds2_s_at | MYB | C-myb gene extracted from Human (c-myb) gene, complete primary cds, and five complete alternatively spliced cds | 21 | Y |
| 41 | X80230_at | CDK9 | mRNA (clone C-2k) mRNA for serine/threonine protein kinase | 21 |  |
| 42 | L05148_at | ZAP70 | Protein tyrosine kinase related mRNA sequence | 21 |  |
| 43 | U29175_at | SMARCA4 | Transcriptional activator hSNF2b | 20 | Y |
| 44 | U89922_s_at | LTB | LTB Lymphotoxin-beta | 19 |  |
| 45 | M12959_s_at | TCRA | TCRA T cell receptor alpha-chain | 19 |  |
| 46 | D63880_at | NCAPD2 | KIAA0159 gene | 19 |  |
| 47 | M28170_at | CD19 | CD19 antigen | 19 |  |
| 48 | J03473_at | PARP1 | ADPRT ADP-ribosyltransferase (NAD+; poly (ADP-ribose) polymerase) | 19 | Y |
| 49 | M84371_rna1_s_at | CD19 | CD19 gene | 18 |  |
| 50 | K01911_at | NPY | NPY Neuropeptide Y | 18 |  |

**Table S22** Description of 50 top-ranked genes for the prostate dataset

| No. | Probe No. | Gene symbol | Description | Frequency | Is cancer gene? |
| --- | --- | --- | --- | --- | --- |
| 1 | 41504_s_at | MAF | Cluster Incl. AF055376:Homo sapiens short form transcription factor C-MAF (c-maf) mRNA, complete cds /cds=(807,1928) /gb=AF055376 /gi=3335147 /ug=Hs.30250 /len=4246 | 1444 | Y |
| 2 | 37639_at | HPN | Cluster Incl. X07732:Human hepatoma mRNA for serine protease hepsin /cds=UNKNOWN /gb=X07732 /gi=32063 /ug=Hs.823 /len=2363 | 1407 |  |
| 3 | 2041_i_at | ABL1 | M14752 /FEATURE= /DEFINITION=HUMABLA Human c-abl gene, complete cds | 941 | Y |
| 4 | 40436_g_at | SLC25A6 | Cluster Incl. J03592:Human ADP/ATP translocase mRNA, 3 end, clone pHAT8 /cds=(0,788) /gb=J03592 /gi=339722 /ug=Hs.164280 /len=1116 | 496 |  |
| 5 | 41381_at | CHD9 | Cluster Incl. AB002306:Human mRNA for KIAA0308 gene, partial cds /cds=(0,3895) /gb=AB002306 /gi=2224556 /ug=Hs.10351 /len=6452 | 338 |  |
| 6 | 863_g_at | SERPINB5 | U04313 /FEATURE= /DEFINITION=HSU04313 Human maspin mRNA, complete cds | 283 | Y |
| 7 | 34840_at | [A2R6W1](http://www.uniprot.org/uniprot/A2R6W1) | Cluster Incl. AI700633:we38g03.x1 Homo sapiens cDNA, 3 end /clone=IMAGE-2343412 /clone_end=3 /gb=AI700633 /gi=4988533 /ug=Hs.4815 /len=565 | 182 |  |
| 8 | 34213_at | WWC1 | Cluster Incl. AB020676:Homo sapiens mRNA for KIAA0869 protein, partial cds /cds=(0,2667) /gb=AB020676 /gi=4240226 /ug=Hs.21543 /len=3408 | 121 |  |
| 9 | 32598_at | NELL2 | Cluster Incl. D83018:Homo sapiens mRNA for nel-related protein 2, complete cds /cds=(96,2546) /gb=D83018 /gi=1827484 /ug=Hs.79389 /len=3198 | 117 |  |
| 10 | 38634_at | RBP1 | Cluster Incl. M11433:Human cellular retinol-binding protein mRNA, complete cds /cds=(125,532) /gb=M11433 /gi=190947 /ug=Hs.101850 /len=716 | 104 |  |
| 11 | 36918_at | GUCY1A3 | Cluster Incl. Y15723:Homo sapiens mRNA for soluble guanylyl cyclase /cds=(523,2595) /gb=Y15723 /gi=3702146 /ug=Hs.75295 /len=2982 | 61 |  |
| 12 | 40024_at | STAC | Cluster Incl. D86640:Homo sapiens mRNA for stac, complete cds /cds=(39,1247) /gb=D86640 /gi=1799567 /ug=Hs.56045 /len=2963 | 56 |  |
| 13 | 41755_at | COBLL1 | Cluster Incl. AB023194:Homo sapiens mRNA for KIAA0977 protein, complete cds /cds=(216,3716) /gb=AB023194 /gi=4589597 /ug=Hs.182527 /len=4834 | 54 |  |
| 14 | 914_g_at | ERG | M21535 /FEATURE= /DEFINITION=HUMERG11 Human erg protein (ets-related gene) mRNA, complete cds | 52 | Y |
| 15 | 39366_at | PPP1R3C | Cluster Incl. N36638:yx88f05.r1 Homo sapiens cDNA, 5 end /clone=IMAGE-268833 /clone_end=5 /gb=N36638 /gi=1157780 /ug=Hs.12112 /len=543 | 52 |  |
| 16 | 36666_at | P4HB | Cluster Incl. M22806:Human prolyl 4-hydroxylase beta-subunit and disulfide isomerase (P4HB) gene /cds=(66,1592) /gb=M22806 /gi=487831 /ug=Hs.75655 /len=2438 | 51 | Y |
| 17 | 33386_at | H1F0 | Cluster Incl. Z97630:Human DNA sequence from clone 466N1 on chromosome 22q12-13 Contains H1F0(H1 histone family, member 0) gene, 2-amino-3-ketobutyrate -CoA ligase( nuclear gene encoding mitochondrial protein), GALR3 (galanin receptor) gene, ESTs, GSSs and CpG islands /cds=(381,965) /gb=Z97630 /gi=4582128 /ug=Hs.226117 /len=2527 | 46 |  |
| 18 | 37599_at | AOX1 | Cluster Incl. AF017060:untitled /cds=(298,4314) /gb=AF017060 /gi=2343154 /ug=Hs.81047 /len=5125 | 45 |  |
| 19 | 38291_at | PENK | Cluster Incl. J00123:Human enkephalin gene /cds=(0,803) /gb=J00123 /gi=182098 /ug=Hs.93557 /len=804 | 43 |  |
| 20 | 496_s_at | IL11RA | U32324 /FEATURE= /DEFINITION=HSU32324 Human interleukin-11 receptor alpha chain mRNA, complete cds | 42 |  |
| 21 | 35710_s_at | STRA13 | Cluster Incl. U95006:Human D9 splice variant A mRNA, complete cds /cds=(3,194) /gb=U95006 /gi=2071992 /ug=Hs.37616 /len=697 | 39 |  |
| 22 | 1708_at | MAPK10 | U07620 /FEATURE= /DEFINITION=HSU07620 Human MAP kinase mRNA, complete cds | 38 |  |
| 23 | 31509_at | RPL13 | Cluster Incl. X64707:H.sapiens BBC1 mRNA /cds=(51,686) /gb=X64707 /gi=29382 /ug=Hs.180842 /len=942 | 38 |  |
| 24 | 38429_at | FASN | Cluster Incl. U29344:Human breast carcinoma fatty acid synthase mRNA, complete cds /cds=(123,7652) /gb=U29344 /gi=915391 /ug=Hs.83190 /len=8460 | 36 | Y |
| 25 | 36589_at | AKR1B1 | Cluster Incl. X15414:Human mRNA for aldose reductase (EC 1.1.1.2) /cds=(45,995) /gb=X15414 /gi=28646 /ug=Hs.75313 /len=1367 | 36 | Y |
| 26 | 38028_at | LMO3 | Cluster Incl. AL050152:Homo sapiens mRNA; cDNA DKFZp586K1220 (from clone DKFZp586K1220) /cds=UNKNOWN /gb=AL050152 /gi=4884363 /ug=Hs.7974 /len=2821 | 36 |  |
| 27 | 1767_s_at | TGFB3 | X14885 /FEATURE=mRNA /DEFINITION=HSTGF31 H.sapiens gene for transforming growth factor-beta 3 (TGF-beta 3) exon 1 (and joined CDS) | 34 |  |
| 28 | 39799_at | FABP5 | Cluster Incl. M94856:Human fatty acid binding protein homologue (PA-FABP) mRNA, complete cds /cds=(48,455) /gb=M94856 /gi=182353 /ug=Hs.153179 /len=662 | 32 |  |
| 29 | 34050_at | ACSM2A | Cluster Incl. AC003034:Homo sapiens Chromosome 16 BAC clone CIT987SK-A-923A4 /cds=(27,713) /gb=AC003034 /gi=3219338 /ug=Hs.98732 /len=965 | 30 |  |
| 30 | 38908_s_at | REV3L | Cluster Incl. AL096744:Homo sapiens mRNA; cDNA DKFZp566H033 (from clone DKFZp566H033) /cds=UNKNOWN /gb=AL096744 /gi=5419873 /ug=Hs.198559 /len=2603 | 30 | Y |
| 31 | 39939_at | COL4A6 | Cluster Incl. D21337:Human mRNA for collagen /cds=(234,5270) /gb=D21337 /gi=466537 /ug=Hs.408 /len=6378 | 29 |  |
| 32 | 38087_s_at | S100A4 | Cluster Incl. W72186:zd69b10.s1 Homo sapiens cDNA, 3 end /clone=IMAGE-345883 /clone_end=3 /gb=W72186 /gi=1382635 /ug=Hs.81256 /len=598 | 28 | Y |
| 33 | 39798_at | RPS28 | Cluster Incl. R87876:yo45h01.r1 Homo sapiens cDNA, 5 end /clone=IMAGE-180913 /clone_end=5 /gb=R87876 /gi=946689 /ug=Hs.153177 /len=483 | 27 |  |
| 34 | 32747_at | ALDH2 | Cluster Incl. X05409:Human RNA for mitochondrial aldehyde dehydrogenase I ALDH I (EC 1.2.1.3) /cds=(36,1586) /gb=X05409 /gi=28605 /ug=Hs.195432 /len=1989 | 27 |  |
| 35 | 40167_s_at | WSB2 | Cluster Incl. AF038187:Homo sapiens clone 23714 mRNA sequence /cds=UNKNOWN /gb=AF038187 /gi=2795907 /ug=Hs.136644 /len=1642 | 27 |  |
| 36 | 33716_at | RAB22A | Cluster Incl. N95443:zb81c12.s1 Homo sapiens cDNA, 3 end /clone=IMAGE-310006 /clone_end=3 /gb=N95443 /gi=1267753 /ug=Hs.19180 /len=611 | 26 |  |
| 37 | 40074_at | MTHFD2 | Cluster Incl. X16396:Human mRNA for NAD-dependent methylene tetrahydrofolate dehydrogenase cyclohydrolase (EC 1.5.1.15) /cds=(15,1049) /gb=X16396 /gi=35070 /ug=Hs.154672 /len=2102 | 25 |  |
| 38 | 37068_at | PLA2G7 | Cluster Incl. U24577:Human LDL-phospholipase A2 mRNA, complete cds /cds=(216,1541) /gb=U24577 /gi=1314245 /ug=Hs.93304 /len=1561 | 25 |  |
| 39 | 37253_at |  | Cluster Incl. X92493:H.sapiens mRNA for STM-7 protein /cds=(419,2041) /gb=X92493 /gi=1045196 /ug=Hs.78406 /len=2764 | 24 |  |
| 40 | 33415_at | NME1 | Cluster Incl. X58965:H.sapiens RNA for nm23-H2 gene /cds=(72,530) /gb=X58965 /gi=35069 /ug=Hs.227823 /len=670 | 24 | Y |
| 41 | 32695_at | HTATSF1 | Cluster Incl. Z97632:dJ196E23.2 (HIV-1 transcriptional elongation factor TAT cofactor TAT-SF1) /cds=(111,2378) /gb=Z97632 /gi=2808417 /ug=Hs.171595 /len=2712 | 24 |  |
| 42 | 41671_at | EML1 | Cluster Incl. U97018:Homo sapiens echinoderm microtubule-associated protein homolog HuEMAP mRNA, complete cds /cds=(362,2515) /gb=U97018 /gi=2104768 /ug=Hs.12451 /len=3962 | 24 |  |
| 43 | 37730_at | SND1 | Cluster Incl. U22055:Human 100 kDa coactivator mRNA, complete cds /cds=(267,2924) /gb=U22055 /gi=799176 /ug=Hs.79093 /len=3480 | 23 |  |
| 44 | 36587_at | EEF2 | Cluster Incl. Z11692:H.sapiens mRNA for elongation factor 2 /cds=(0,2576) /gb=Z11692 /gi=31107 /ug=Hs.75309 /len=3080 | 23 |  |
| 45 | 38406_f_at | At4g25845 | Cluster Incl. AI207842:ao89h09.x1 Homo sapiens cDNA, 3 end /clone=IMAGE-1953089 /clone_end=3 /gb=AI207842 /gi=3769784 /ug=Hs.8272 /len=771 | 22 |  |
| 46 | 41485_at | LDHA | Cluster Incl. X02152:Human mRNA for lactate dehydrogenase-A (LDH-A, EC 1.1.1.27) /cds=(97,1095) /gb=X02152 /gi=34312 /ug=Hs.2795 /len=1661 | 22 |  |
| 47 | 39608_at | SIM2 | Cluster Incl. U80456:Human transcription factor SIM2 long form mRNA, complete cds /cds=(92,2095) /gb=U80456 /gi=2062416 /ug=Hs.27311 /len=3921 | 22 |  |
| 48 | 35720_at | WDR47 | Cluster Incl. AB020700:Homo sapiens mRNA for KIAA0893 protein, complete cds /cds=(223,2982) /gb=AB020700 /gi=4240274 /ug=Hs.3830 /len=4195 | 21 |  |
| 49 | 39154_at | GADD45G | Cluster Incl. AI952982:wp98b06.x1 Homo sapiens cDNA, 3 end /clone=IMAGE-2469779 /clone_end=3 /gb=AI952982 /gi=5745292 /ug=Hs.9701 /len=816 | 21 | Y |
| 50 | 1980_s_at | NME1 | X58965 /FEATURE= /DEFINITION=HSNM23H2G H.sapiens RNA for nm23-H2 gene | 21 | Y |

# 6 Pathway analysis of the genes selected by HBSA-KNN

The top 50 genes for each datasetare selected and analyzed in terms of its biological pathways on the website http://vortex.cs.wayne.edu/projects.htm. The Tables S23-S28 are the results of the most significant pathways involved in the selected genes.

**Table S23**Ten pathways with the smallest *p*-values in the Prostate dataset

| No. | Pathways | Pathway Genes  in the Chip | Input Genes  in the Chip | Pathway  Genes | *p*-Values |
| --- | --- | --- | --- | --- | --- |
| 1 | Cell cycle | 100 | 3 | 112 | 0.015007 |
| 2 | Insulin signaling pathway | 123 | 3 | 138 | 0.025858 |
| 3 | p53 signaling pathway | 53 | 2 | 68 | 0.030855 |
| 4 | Ribosome | 60 | 2 | 91 | 0.038737 |
| 5 | Pancreatic cancer | 71 | 2 | 73 | 0.052473 |
| 6 | Chronic myeloid leukemia | 72 | 2 | 76 | 0.053797 |
| 7 | Colorectal cancer | 74 | 2 | 84 | 0.056481 |
| 8 | ErbB signaling pathway | 80 | 2 | 87 | 0.064807 |
| 9 | Biosynthesis of unsaturated fatty acids | 18 | 1 | 23 | 0.089698 |
| 10 | MAPK signaling pathway | 216 | 3 | 265 | 0.101732 |

**Table S24**Ten pathways with the smallest *p*-values in the DLBCL dataset

| No. | Pathways | Pathway Genes  in the Chip | Input Genes  in the Chip | Pathway  Genes | *p*-Values |
| --- | --- | --- | --- | --- | --- |
| 1 | Cell cycle | 100 | 3 | 112 | 0.017714 |
| 2 | Antigen processing and presentation | 70 | 2 | 88 | 0.05713 |
| 3 | Hematopoietic cell lineage | 83 | 2 | 88 | 0.076972 |
| 4 | Leukocyte transendothelial migration | 95 | 2 | 116 | 0.096918 |
| 5 | Cell adhesion molecules (CAMs) | 104 | 2 | 133 | 0.112738 |
| 6 | Huntington''s disease | 26 | 1 | 30 | 0.134546 |
| 7 | DNA replication | 32 | 1 | 35 | 0.16298 |
| 8 | Graft-versus-host disease | 32 | 1 | 42 | 0.16298 |
| 9 | Allograft rejection | 33 | 1 | 38 | 0.167629 |
| 10 | Notch signaling pathway | 33 | 1 | 46 | 0.167629 |

**Table S25**Ten pathways with the smallest *p*-values in the leukemia dataset

| No. | Pathways | Pathway Genes in the Chip | Input Genes  in the Chip | Pathway  Genes | p-Values |
| --- | --- | --- | --- | --- | --- |
| 1 | Primary immunodeficiency | 32 | 4 | 35 | 2.26E-05 |
| 2 | Hematopoietic cell lineage | 83 | 4 | 88 | 9.48E-04 |
| 3 | B cell receptor signaling pathway | 59 | 3 | 64 | 0.003715 |
| 4 | $hsa05131$ | 40 | 2 | 51 | 0.018965 |
| 5 | Pathogenic Escherichia coli infection | 40 | 2 | 51 | 0.018965 |
| 6 | $hsa03450$ | 9 | 1 | 13 | 0.046836 |
| 7 | Cell cycle | 100 | 2 | 112 | 0.098625 |
| 8 | Basal transcription factors | 27 | 1 | 34 | 0.134154 |
| 9 | Base excision repair | 27 | 1 | 33 | 0.134154 |
| 10 | Jak-STAT signaling pathway | 123 | 2 | 153 | 0.138677 |

**Table S26**Ten pathways with the smallest *p*-Values in the colon tumor dataset

| No. | Pathways | Pathway Genes  in the Chip | Input Genes  in the Chip | Pathway  Genes | *p*-Values |
| --- | --- | --- | --- | --- | --- |
| 1 | Base excision repair | 27 | 2 | 33 | 0.008211 |
| 2 | Bladder cancer | 42 | 2 | 42 | 0.019192 |
| 3 | Ribosome | 60 | 2 | 91 | 0.037245 |
| 4 | Complement and coagulation cascades | 64 | 2 | 69 | 0.041884 |
| 5 | ECM-receptor interaction | 76 | 2 | 87 | 0.056995 |
| 6 | Tight junction | 101 | 2 | 135 | 0.093326 |
| 7 | Mismatch repair | 21 | 1 | 22 | 0.101763 |
| 8 | Glycan structures - degradation | 23 | 1 | 30 | 0.110909 |
| 9 | Homologous recombination | 23 | 1 | 27 | 0.110909 |
| 10 | DNA replication | 32 | 1 | 35 | 0.150951 |

**Table S27**Ten pathways with the smallest *p*-values in the ALL dataset

| No. | Pathways | Pathway Genes  in the Chip | Input Genes  in the Chip | Pathway  Genes | *p*-Values |
| --- | --- | --- | --- | --- | --- |
| 1 | Asthma | 27 | 2 | 30 | 0.008211 |
| 2 | Hematopoietic cell lineage | 83 | 3 | 88 | 0.008544 |
| 3 | Primary immunodeficiency | 32 | 2 | 35 | 0.011417 |
| 4 | Graft-versus-host disease | 32 | 2 | 42 | 0.011417 |
| 5 | Allograft rejection | 33 | 2 | 38 | 0.012115 |
| 6 | Cell adhesion molecules (CAMs) | 104 | 3 | 133 | 0.015725 |
| 7 | Type I diabetes mellitus | 39 | 2 | 44 | 0.016677 |
| 8 | Autoimmune thyroid disease | 44 | 2 | 53 | 0.020952 |
| 9 | $hsa03450$ | 9 | 1 | 13 | 0.044924 |
| 10 | Antigen processing and presentation | 70 | 2 | 88 | 0.049225 |

**Table S28**Ten pathways with the smallest *p*-values in the SRBCT dataset

| No. | Pathways | Pathway Genes  in the Chip | Input Genes  in the Chip | Pathway  Genes | p-Values |
| --- | --- | --- | --- | --- | --- |
| 1 | Cell adhesion molecules (CAMs) | 104 | 3 | 133 | 0.017624 |
| 2 | p53 signaling pathway | 53 | 2 | 68 | 0.032078 |
| 3 | Mismatch repair | 21 | 1 | 22 | 0.105956 |
| 4 | Homologous recombination | 23 | 1 | 27 | 0.115454 |
| 5 | Asthma | 27 | 1 | 30 | 0.134154 |
| 6 | Thyroid cancer | 27 | 1 | 29 | 0.134154 |
| 7 | DNA replication | 32 | 1 | 35 | 0.156986 |
| 8 | Graft-versus-host disease | 32 | 1 | 42 | 0.156986 |
| 9 | Allograft rejection | 33 | 1 | 38 | 0.161481 |
| 10 | Nucleotide excision repair | 39 | 1 | 43 | 0.187963 |

# 7 Comparison of classification accuracy for three experimental methods

We adopt three experimental methods to evaluate the classification performance of the selected gene list. The three methods are HBSA-SVM(Biased), HBSA-KNN-SVM(Biased) and HBSA-KNN-SVM(Unbiased), respectively, which are described in the main text. We find thatthe classification accuracy of the HBSA-KNN-SVM(Unbiased) is usually slightly lower than that of the HBSA-KNN-SVM(Biased).

|  |  |
| --- | --- |
|  |  |
|  |  |

**Fig.S2**. Classification accuracy of different number of the top-ranked genes for the six test sets.

# 8 Comparison of experimental results with 0-1 normalization

We find that adopting different normalization method might obtain different experimental results. Table S29 lists the accuracy of top-ranked genes with 0-1 normalization method for three methods: HBSA-KNN, PAM and ClaNC. Note that for our HBSA-KNN method we just apply 0-1 normalization method to normalize dataset in obtaining prediction accuracy on test set. In other words, the procedure of gene selection presented here is the same as the HBSA-KNN in main body, that is, the same z-score method is adopted to normalize dataset in gene selection using the HBSA-KNN except predicting accuracy on test set. The results indicate that PAM is sensitive to different normalization method and it is also not suitable for the cross-platform dataset, PAM is inferior to ClaNC in classification performance.

Although the top-ranked genes paly a crucial role in the development of tumor, there exist many redundent genes among these important genes, which also leads to the drop of classification accuracy. For the prostate dataset, although the first two genes MAF and HPN in Table S22 selected by the HBSA-KNN can obtain 88.24% prediction accuracy, in fact, only the single gene (the second gene HPN) can obtain 97.06% prediction accuracy. Moreover, for the prostate dataset the gene subset consisting of the second, third and fourth genes (HPN, ABL1 and SLC25A6) can obtain 100% prediction accuracy. Therefore, our HBSA-KNN method is still consistently superior to the ClaNC method in accuracy on the six test sets when the number of gene subset selected is smallenough. Our results indicate that the small top-ranked gene subsets include more important tumor-related genes.

**Table S29** Comparisons with the PAM and ClaNC methods in accuracy obtained on test set after adopting another normalization method 0-1 normalization preprocess.

| No. | Methods | Dataset | Number of the top-ranked genes | | | | | | | | | |
| --- | --- | --- | --- | --- | --- | --- | --- | --- | --- | --- | --- | --- |
|  |  |  | 2 | 3 | 4 | 5 | 6 | 7 | 8 | 20 | 40 | 60 |
| 1 | HBSA-KNN | Leukemia | 84.62 | 98.08 | 92.31 | 94.23 | 80.77 | 80.77 | 80.77 | 82.69 | 84.62 | 88.46 |
|  |  | DLBCL | 90.48 | 90.48 | 90.48 | 80.95 | 85.71 | 90.48 | 90.48 | 90.48 | 90.48 | 95.24 |
|  |  | Prostate | 88.24 | 82.35 | 91.18 | 88.24 | 85.29 | 85.29 | 85.29 | 82.35 | 85.29 | 79.41 |
|  |  | SRBCT | 80 | 95 | 95 | 100 | 95 | 90 | 90 | 95 | 95 | 95 |
|  |  | ALL | 64 | 76 | 82 | 87 | 92 | 94 | 94 | 95 | 97 | 99 |
|  |  | Colon | 65 | 75 | 75 | 75 | 75 | 75 | 75 | 75 | 75 | 75 |
| 2 | PAM | Dataset | Number of the selected genes | | | | | | | | | |
|  |  |  | 2 | 4 | 6 | 8 | 10 | 12 | 16 | 20 | 40 | 60 |
|  |  | Leukemia | 46.15 | 61.54 | 67.31 | 71.15 | 80.77 | 80.77 | 80.77 | 80.77 | 86.54 | 90.38 |
|  |  | DLBCL | 66.67 | 66.67 | 66.67 | 66.67 | 66.67 | 71.43 | 71.43 | 71.43 | 71.43 | 85.71 |
|  |  | Prostate | 97.06 | 94.12 | 94.12 | 94.12 | 97.06 | 97.06 | 97.06 | 97.06 | 97.06 | 97.06 |
|  |  | SRBCT | 40 | 45 | 45 | 55 | 55 | 55 | 75 | 90 | 90 | 90 |
|  |  | ALL | 43 | 43 | 46 | 56 | 69 | 82 | 82 | 85 | 86 | 86 |
|  |  | Colon | 60 | 60 | 70 | 70 | 70 | 75 | 75 | 75 | 75 | 75 |
| 3 | ClaNC | Dataset | Number of the selected genes per subclass | | | | | | | | | |
|  |  |  | 1×*k** | 2×*k* | 3×*k* | 4×*k* | 5×*k* | 6×*k* | 7×*k* | 8×*k* | 9×*k* | 10×*k* |
|  |  | Leukemia | 78.85 | 86.54 | 82.69 | 82.69 | 76.92 | 82.69 | 86.54 | 90.38 | 90.38 | 90.38 |
|  |  | DLBCL | 85.74 | 76.19 | 76.19 | 80.95 | 90.48 | 80.95 | 90.48 | 85.71 | 80.95 | 80.95 |
|  |  | Prostate | 79.41 | 97.06 | 94.12 | 94.12 | 97.06 | 94.12 | 94.12 | 94.12 | 94.12 | 94.12 |
|  |  | SRBCT | 85 | 90 | 95 | 95 | 95 | 95 | 95 | 95 | 95 | 95 |
|  |  | ALL | 87 | 93 | 95 | 97 | 97 | 95 | 96 | 96 | 96 | 96 |
|  |  | Colon | 65 | 65 | 70 | 70 | 75 | 80 | 80 | 75 | 75 | 75 |

** k denotes the number of the tumor subclasses for each dataset, which ranges from two to six. For example, the number of the selected gene ranges from two to sixty for ALL dataset.*

*For the prostate dataset, although the first two genes MAF and HPN in Table S22 selected by HBSA-KNN can obtain 88.24% prediction accuracy, in fact, only the second gene HPN can obtain 97.06% prediction accuracy. Moreover, for the prostate dataset the gene subset consisting of the second, third and fourth genes (HPN, ABL1 and SLC25A6) can obtain 100% prediction accuracy.*

# 9 Partial results on the colon tumor dataset

It was found that genes with similar expression pattern as features might degrade the classification performance in some cases. For the colon tumor dataset, two top-ranked genes {M80815, R87126} selected by HBSA-KNN can obtain only 65% prediction accuracy on the corresponding test set, while the single gene R87126, ranked the second as shown in Table S19, can obtain 80% prediction accuracy. The similar expression pattern of the two genes {M80815, R87126} can be seen from their respective scatter plots on the training set and test set, shown in Fig. S2. It suggests that one gene is enough for obtaining the highest prediction accuracy for this dataset, and the genes with similar expression pattern might degrade the classification performance.

|   Training set (42 samples) |   Test set (20 samples) |
| --- | --- |

**Fig. S3.**Scatter plot of top two genes {M80815, R87126} selected by the HBSA-KNN for the colon tumor dataset. Here, label 1 denotes tumor state and label 2 denotes the normal state.

To analyze the reliability of classifying colon tumor dataset, the confidence levels of 20 test samples are shown in Table S30 obtained by using HBSA-SVM(Biased). We find that the samples 9 and 13 have very high confidence levels, 2.8961 and 29, respectively, which indicates that the two samples might be labeled mistakenly. The samples 7 and 8 are narrowlycorrectly classified owing to their low confidence levels, 1.0833 and 1.0408, respectively.

**Table S30** Confidence levels of 20 test samplesby HBSA-SVM(Biased)-based ensemble classifier on colon dataset.

| 20 samples  (No.) * | #Tumor subclass votes | #Normal subclass votes | Confidence level | Correct? ** |
| --- | --- | --- | --- | --- |
| 1 (43) | 91 | 209 | 2.2967 | C |
| 2 (44) | 299 | 1 | 299 | C |
| 3 (45) | 215 | 85 | 2.5294 | C |
| 4 (46) | 300 | 0 | 300 | C |
| 5 (47) | 298 | 2 | 149 | C |
| 6 (48) | 111 | 189 | 1.7027 | C |
| 7 (49) | 156 | 144 | 1.0833 | C |
| 8 (50) | 147 | 153 | 1.0408 | C |
| 9 (51) | 223 | 77 | 2.8961 | **E** |
| 10 (52) | 300 | 0 | 300 | C |
| 11 (53) | 288 | 12 | 24 | C |
| 12 (54) | 24 | 276 | 11.5 | C |
| 13 (55) | 290 | 10 | 29 | **E** |
| 14 (56) | 160 | 140 | 1.1429 | C |
| 15 (57) | 235 | 65 | 3.615 | C |
| 16 (58) | 244 | 56 | 4.3571 | C |
| 17 (59) | 279 | 21 | 13.2857 | C |
| 18 (60) | 101 | 199 | 1.9703 | C |
| 19 (61) | 300 | 0 | 300 | C |
| 20 (62) | 130 | 170 | 1.3077 | C |

** The number inparentheses denotes the serial number of sample in original colon tumor dataset.*

*** “C” means the sample classified correctly and “E” means the sample classified mistakenly.*

# 10 Functional analysis of the top-ranked genes selected by HBSA-SVM

Biologically the experimental results also provedthatthe selected genes with high classification accuracy are functionally related to carcinogenesis or tumor histogenesis. Thus we could infer that a few top-ranked genes (see Supplementary Tables S5-S10) may be very important for tumor diagnosis.

For the leukemia dataset, CD33 (M23197) is expressed on the surface of normal myeloid cells and on the malignant blast cells in most cases of acute myeloid leukemia (AML) but not on normal hematopoietic pluripotent stem cells [73]. Using a humanized anti-CD33 antibody conjugated with calicheamicin, the effectiveness of in vivo ablation of CD33+ cellsto treat patients with acute myeloid leukemiawere proved by a higher portion of remission[74]. Zyxin (X95735) is a gene correlated to leukemia of ALL and Zyxin protein possesses LIM domain which is known to interact with leukemogenic bHLH proteins [100]. It is also localized at focal contacts in adherent erythroleukemia cells [101]. TCF3 (M31523) is involved in 19p13 chromosome rearrangement andacts as a tumor suppressor gene in B-cell precursor acute lymphoblastic leukemia[78]. CCND3 (M92287_at) is involved in cell development and adhesion. TOP2B (Z15115) is a target of the antileukemia drug etoposide [63]. In addition, the CD63 (X62654)and CD81 (M33680)genes belong to a newly defined family of genes formembrane proteins including CD33, which was recognized by monoclonal antibodies inhibitory to human T cell leukemia virus type 1-induced syncytium formation[77]. The EIF3F(U94855) gene,located at human chromosome band 11p15.4,plays an important role in translation initiation. Chromosomal abnormalities at 11p15 have been seen in leukemia[102]. In aggressive disease, the chronic lymphocytic leukemia cells usually express an unmutated immunoglobulin heavy-chain variable-region gene and the 70-kD zeta-associated protein (ZAP70)[103]. [Vinante](http://www.ncbi.nlm.nih.gov/sites/entrez?Db=pubmed&Cmd=Search&Term=%22Vinante%20F%22%5BAuthor%5D&itool=EntrezSystem2.PEntrez.Pubmed.Pubmed_ResultsPanel.Pubmed_DiscoveryPanel.Pubmed_RVAbstractPlus)*et al*. [104] demonstrated that leukemic cells in acute myeloid leukemia are equipped with the functional apparatus for IL8 production. Since IL-8 displays a wide range of biological activities, including the regulation of some membrane molecules relevant to adhesion and migration processes, its production by acute myeloid leukemia blasts might be of relevance to the pattern of leukemic growth. ZAP70 (L05148) is solely expressed in poor prognosis chronic lymphocytic leukemia and implicated in enhanced B cells receptor signaling.Its expression may provide targets for therapies [105]. We can also infer that APLP2 (L09209) can be linked with leukemia from our experimental results, although it was reported that APLP2 is not relevant to leukemia.

For the SRBCT dataset,neurofibromatosis 2(NF2) is an autosomal dominant disease characterized by tumors called schwannomas involving in the acoustic nerve. The disorder is caused by mutations of the NF2 (769716) gene resulting in the absence or inactivation of the protein product. The protein product of NF2 is commonly called merlin (but also Neurofibromin 2 and Schwannomin) and functions as a tumor suppressor. However, the mechanism by which merlin suppresses [cell proliferation](javascript:if(window.name=='')%20%7b%7b%20window.location.href='./nil';%20%7d%7d%20else%20%7b%7b%20NPEml('MeSH',23267);%20%7d%7d) is not fully understood[106]. FCGRT (770394) is a EWS-specific signature. It is well established that caveolin-1(377461) isa tumor suppressor gene. Caveolin-1 can also function as a tumor metastasispromotingmolecule, which is unrelated to its function of cell growth inhibition[107].Caveolin-1 can promote the malignant phenotype in EWS carcinogenesis[108]. The interaction of integrin-linked kinase (ILK) and caveolin-1 may be a useful target for genetic screening of human neuroblastoma cells[109]. Antigen identified by monoclonal antibodies 12E7 (1435862) is a sensitive marker for the Ewing's sarcoma/peripheral neuroectodermal group of tumors and is useful in distinguishing them from neuroblastoma and blastema-rich nephroblastoma[110]. AF1Q (812105) is a myeloid/lymphoid or mixed-lineage leukemia marker, which is necessary for neuronal differentiation [111]. SCGA (796258) has been linked to the onset of mammary tumorigenesis [112].

For the ALL dataset, LAIR1 (37470_at) is shown to be absent in high-risk CLL and expresseddifferently on intermediate- and low-risk CLL and the intensity of its expression, which is always significantly lower than that in healthy donors, correlates with disease stage and progression[113]. PARP-1 (41146_at) is important in human leukemia cells to connect cell cycle progression and control of differentiation. Expression of the gene AKAP12 (37680_at) wasdecreased in the samples of acute leukemia and associated with an inferior overall survival[114]. The TEL-AML1 expressing line PER-145 shows high expression of PCLO (37780_at) and I DI1 (36985_at), which is a prominent feature of leukemia cells with t(1;19) translocation [115]. Pottier *et al*. [116] identified nuclear protein poly (ADP-ribose) polymerase family, member 1 (PARP1, 1287_at) as a nuclear protein binding to the SMARCB1 promoter and showed that the -228 SNP significantly increased reporter activity in human ALL (acute lymphoblastic leukemia) cell lines and altered PARP1 binding affinity. The somatic loss of BLNK (38242_at) and concomitant mutations result in constitutive activation of Jak/STAT5 pathway which lead to the generation of pre-B-cell leukemia[117]. Some other genes such as MPP1 (32207_at) and PTP4A3 (36008_at)also correlate with tumor genesis. These genes might participate in the process of leukemia.

For the colon tumor dataset, IL-8 (M26383), a pro-inflammatory cytokine and immunomodulatory mediator, plays important roles in[angiogenesis](http://www.ncbi.nlm.nih.gov/entrez/utils/fref.fcgi?http://amigo.geneontology.org/cgi-bin/amigo/go.cgi?view=details&depth=1&query=1525)[118], [cell cycle arrest](http://www.ncbi.nlm.nih.gov/entrez/utils/fref.fcgi?http://amigo.geneontology.org/cgi-bin/amigo/go.cgi?view=details&depth=1&query=7050), [intracellular signaling cascade](http://www.ncbi.nlm.nih.gov/entrez/utils/fref.fcgi?http://amigo.geneontology.org/cgi-bin/amigo/go.cgi?view=details&depth=1&query=7242), [negative regulation of cell proliferation](http://www.ncbi.nlm.nih.gov/entrez/utils/fref.fcgi?http://amigo.geneontology.org/cgi-bin/amigo/go.cgi?view=details&depth=1&query=8285), and [regulation of cell adhesion](http://www.ncbi.nlm.nih.gov/entrez/utils/fref.fcgi?http://amigo.geneontology.org/cgi-bin/amigo/go.cgi?view=details&depth=1&query=30155)[119]. It was noticedthat IL-8 (M26383) is over expressed in some of the colon carcinoma cells and stimulated by some factors, such as TNF [120,121], hPepT1[122],suggesting that it is implicated in the aggressiveness and metastasis of colon cancer cells, immune responses associated with tumor growth of colon carcinoma. Páez De La Cadena, et al. [123] demonstrated that the α-L-fucosidase content (either as enzymatic activity or as enzymatic protein) is lower in primary colorectal tumours at advanced stages than in primaries at early stages.M76378,encoding human cysteine-rich protein (CRP), as a cancer marker, was reported to be lower expressed and involved in many types of cancers including colon cancer [124].GCAP-II gene (Z50753)has a high level of expression in human colon, which indicates a pivotal role in cGMP-mediated functions of the colon.It stimulates cGMP generation in T84 cells (colonic carcinoma cell line)[125].CKS2 is expressed at significantly higher levels incorrelation withprogression andaggressiveness of colon cancer[126].VIP (M36634) was characterized and localized in the neoplasticcells ofcolonic cancer [127]. As an interesting target to promote apoptosis in cancer cells, CSNK2A1 (M55265) is one of the catalytic subunitsof the Casein kinase 2[128]. Zhou *et al*. [129] found that the 1q31.3-32.1 region might harbor one or more colorectal cancer related tumor suppressor gene(s) through detailed deletion mapping, and presented the first evidence that CSRP1 (M76378) might be involved in the progression of colorectal cancerby microarray-based high-throughput screening of candidate genes located in this region and by subsequent database searching.

For the DLBCL dataset, aberrant somatic hypermutation of the first gene, RhoH (Z35227), is associated with diffuse large B cell lymphoma[130]. CIRBP (D78134_at) is significantly over-expressed for the FL subtype and MCM7 (D55716_at) is obviously under-expressed for the FL subtype [66]. TRIB2 (D87119)plays an important role in survival factor withdrawal-induced apoptosis of TF-1 erythroleukemia cells[131].There were 3 discrete subsets ofDLBCL—“oxidative phosphorylation,” “B-cellreceptor/proliferation,” and “host response”(HR)--identified characterized usinggene set enrichment analysis andconfirmed in an independent series[132].HRtumors had more abundant monocyte/macrophage and dendriticcellsthat transcriptmolecules required for efficient antigenprocessing including certain HLA class I antigens, such as HLA-A (M94880). RanBP1, a small cytosolic protein, is a major regulator of the Ran GTPase that controls several cellular processes including nucleo-cytoplasmic transport, RNA processing, cell cycle progression, mitotic spindle formation, and post-mitotic nuclear assembly[133]. RanBP1 (D38076) is over-expressed in several transformed cell lines. Because the RanBP1 gene is a regulatory target of E2F- and retinoblastoma-related factors deregulated in many tumors, up-regulation of RanBP1 may be part of a regulatory mechanism altered during oncogenesis.ATRX(U72935) modifies gene expression by affecting chromatin. Mutationsin ATRX cause changes in the DNA methylation pattern. Underexpressionof ATRX may favor proliferationofAML andDLBCL blasts[134]. Further, some other genes participate in the immune system activity, which has some linkage with the lymphomas (see Supplementary Table S8).

Most genes with the highest frequency by our method on prostate cancer dataset are demonstrated to be connected with prostate in previous studies, and among the first 50 highest frequently selected genes by HSBA-SVM, 13 genes are known cancer genes as listed in Supplementary Table S22. Study on the association of 11 single nucleotide polymorphisms (SNPs) in the ranked first gene, HEPSIN gene (HPN), with prostate cancer in men of European ancestry demonstrate that a major 11-locus haplotype is significantly associated with prostate cancer, which supports that HPN (X07732) is a potentially important candidate gene involved in prostate cancer susceptibility [79]. Another gene, ERG (M21535), a known cancer gene, ranked 14th, whose alterations in the onset and progression of a large subset of prostate cancer [135] plays critical roles. TSPAN1 (34775), ranked 15th, is a new member of the tetraspanin superfamily 4, which plays an important role in cell signal transmission, regulation, adherence, mobility, proliferation and differentiation. It can be expressed in many kinds of human prostate tumor [136]. It has been shown that S100A4 (38087), ranked the 21st, is over-expressed during progression of cancer of the prostate gland in humans. Saleem et al. [137] provided evidence to support the hypothesis that S100A4 plays a role in invasiveness of human cancer of the prostate gland through the transcriptional regulation of matrix metalloproteinase (MMP)-9.

# 11Network analysis of the top 10 genes selected by HBSA-KNN

Since most protein function through protein-protein interactions, a protein’s function can be represented by its parterners. Network-based analyses of the top 10 genes for the leukemia and prostate datasets are presented in Fig. S3, and that of the SRBCT, Colon, ALL and DLBCL datasets are shown in Figs. S4-S6.

| 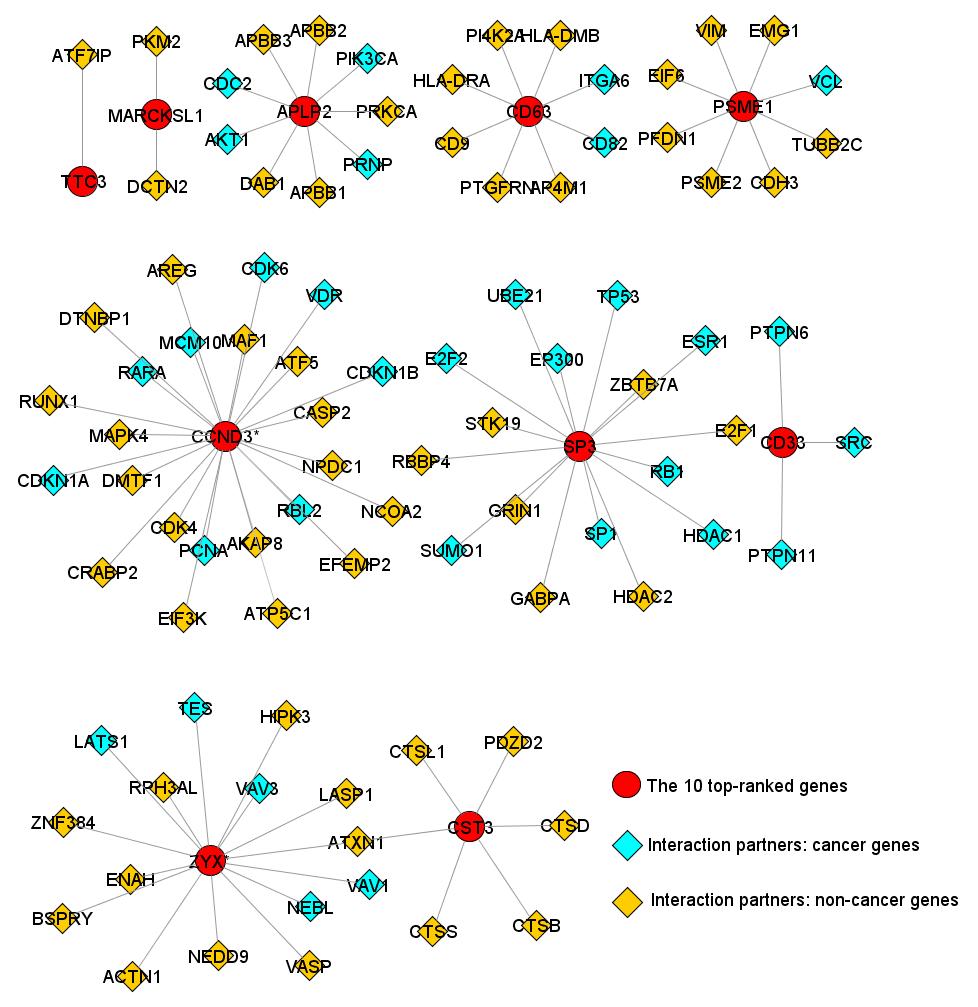 | 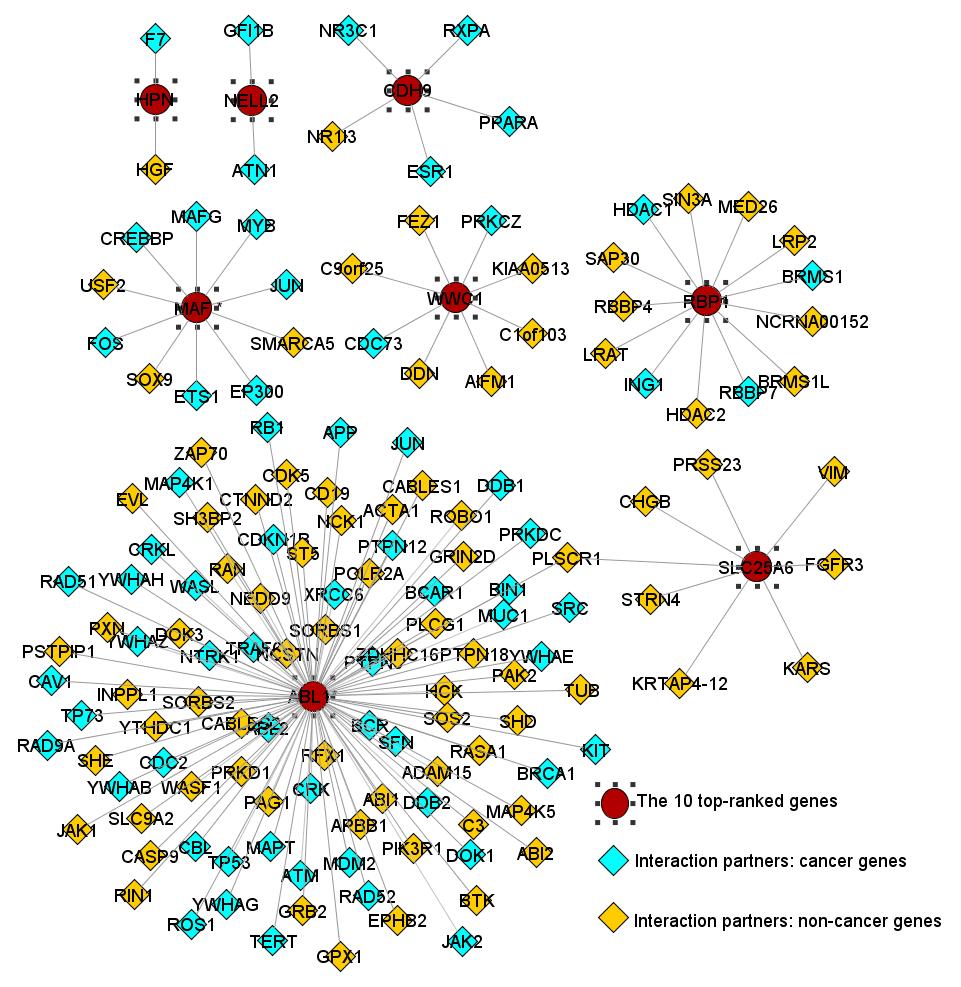 |
| --- | --- |

**Fig. S4**. Protein-protein interaction networks associated with the respective top 10 genes ofleukemia (left) and prostate cancer (right) datasets. The red-circle nodes represent the top 10 genes selected by our method, of which, those listed with an asteroid sign have been identified to be known cancer genes. The diamonds represent the direct interaction partners of the selected genes, of which, the blue diamond nodes are known cancer susceptibility genes.

Among the 10 top-ranked genes for the SRBCT dataset, two (CAV1 and NF2) are cancer genes with their respective CLD of 8 and 26; sevenother genes(CD99,IGF2,Lsp1,FCGRT,CDH2,MAP1B and ELF1) , which are respectiveley ranked first, third, fifth, seventh, eighth, ninth and tenth as shown in Table S17, are directly linked with cancer genes. The cancer linker degrees of the seven genes(the number of the directed interacting cancer proteins), are 2, 7, 1, 2, 6, 6 and 5, respectively. We conclude that these nine genes are cancer related. The remaining one gene, MLLT11, which expresses in leukemia cells, has no linkage in Human Protein Reference Database (HPRD). Chang et al. [138]provide functional evidences that overexpression of [AF1Q](javascript:if(window.name=='')%20%7b%7b%20window.location.href='./nil';%20%7d%7d%20else%20%7b%7b%20open_HOME('/UniPub/iHOP/go?ID1=95997');%20%7d%7d%20)(a synonym of MLLT11) leads to a more progression in human [breast cancer](javascript:if(window.name=='')%20%7b%7b%20window.location.href='./nil';%20%7d%7d%20else%20%7b%7b%20NPEml('MeSH',261);%20%7d%7d%20). Interestingly, CAV1a possible cancer hub gene, directly links with the two cancer genes:CDH2 and NF2,which may be useful for further exploring the cancer related pathways.

For the ALL dataset, PARP1, ranked sixth, is a known cancer genes. Five other genes, i.e. BLANK, MPP1, LAIR1, DNTT and PTTG1IP, ranked third, fourth, fifth, ninth and tenth, have a corresponding CLD of 7, 2, 1, 2 and 1, respectively. Therefore, these five genes are likely cancer biomarkers.IDI1 (Human homolog of yeast IPP isomerase)were also identified as a discriminative gene for pediatric accuate lymphoblastic leukemia by Ross et al. [63,138]. Leukemia cells stimulated with GM-CSF were blocked in the G0/G1 phase of the cell cycle and underwent apoptosis within 4 days after the engagement of LAIR-1(leukocyte-associated Ig-like receptor-1).LRMP (lymphoid-restricted membrane protein (Jaw1)) is downregulated during lymphoid differentiation.The relationship with ALL cancer of thethree other genes includingIDI1, LRMP,and 33821_at probe for two novel ribosomal proteins requires further study.

For the colon tumor dataset, Fig. S5 shows that five genes, i.e. DARS, IL8, VIP, CD37 and CKS2, ranked third, fifth, eighth, ninth and tenth (shown in Table S19), have a direct interaction with known cancer genes with CLD of 1, 1, 1,1 and 3, respectively.FUCA1, MT2A and FXN, ranked first, fourth and sixth, respectively, have no interaction parterners in HPRD. FUCA1 encodesalpha-L-fucosidase,a lysosomal enzyme involved in the degradation offucose-containing glycoproteins and glycolipids.Evidence indicates that the presence of aberrant *α*1→2fucosylation pathways is responsible for the accumulation of large quantities of Leb and Y antigens in human colorectal carcinoma[139]. Metallothioneins encoded by MT2A have a high content of cysteine residues that bind various heavy metals and are transcriptionally regulated by both heavy metals and glucocorticoids.FXNregulates mitochondrial iron transport and respirationand anti-apoptotic process by preventing mitochondrial damage and reactive oxygen species (ROS) production. Schulz*et al*.[140]found that induction of oxidative metabolism by mitochondrial [frataxin](javascript:if(window.name=='')%20%7b%7b%20window.location.href='./nil';%20%7d%7d%20else%20%7b%7b%20open_HOME('/UniPub/iHOP/go?ID1=88335');%20%7d%7d%20) inhibits cancer growth,which supports the view that an increase in oxidative metabolism induced by mitochondrial [frataxin](javascript:if(window.name=='')%20%7b%7b%20window.location.href='./nil';%20%7d%7d%20else%20%7b%7b%20open_HOME('/UniPub/iHOP/go?ID1=88335');%20%7d%7d%20) may inhibit cancer growth in mammals. The mRNA expression of myosin Va is increased in a number of highly metastatic cancer cell lines and metastatic colorectal cancer tissues[141]. CD37 is involved in TCR signaling pathway [142] that prevents autoimmune responses of many cancer cells. It is reasonable to infer that CD37 may be involved in the immune escape of the cancer cells [143]. As mentioned above, all the top 10 genes are closely related to colon cancers.


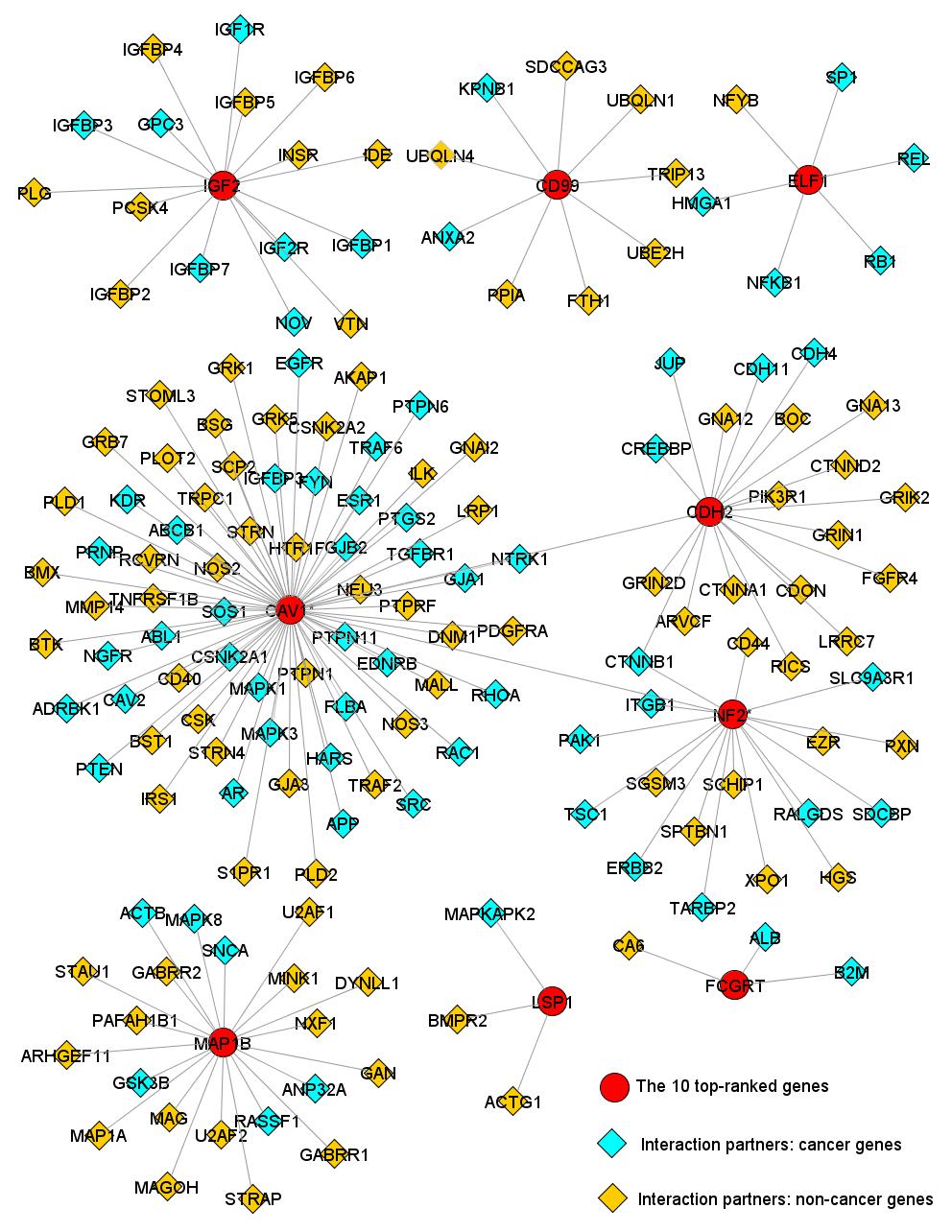


**Fig. S5.** Protein-protein interaction networks associated with the respective ten top-ranked genes ofthe SRBCT dataset. As illustrated in Fig.7 of the main text, the red-ellipse nodes represent the 10 top-ranked genes selected by our method.The diamond nodesrepresent the direct interaction partners of the selected genes, of which, the blue diamond nodesare knowncancer susceptibility genes.


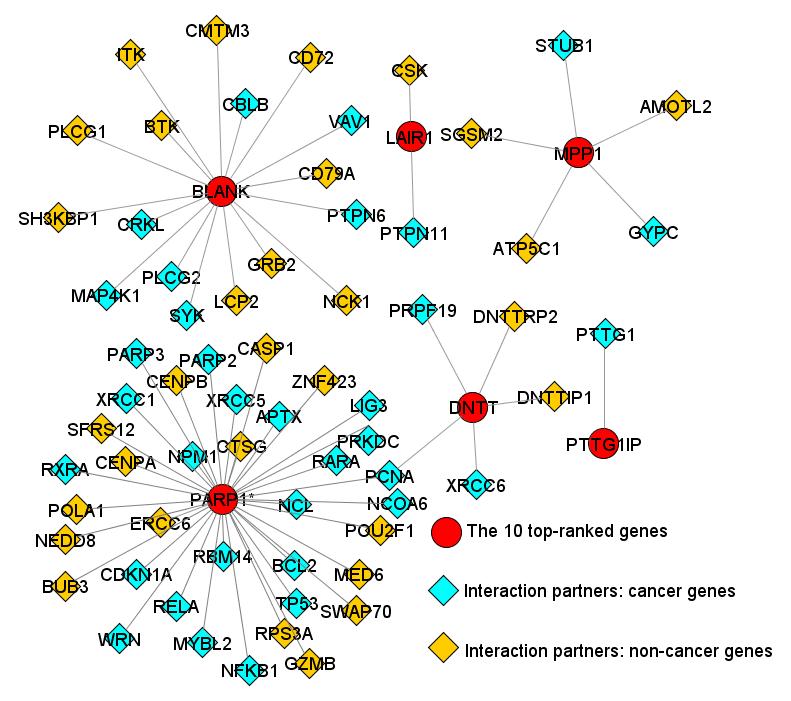


**Fig. S6.** Protein-protein interaction networks associated with the respective ten top-ranked genes ofthe ALL dataset.


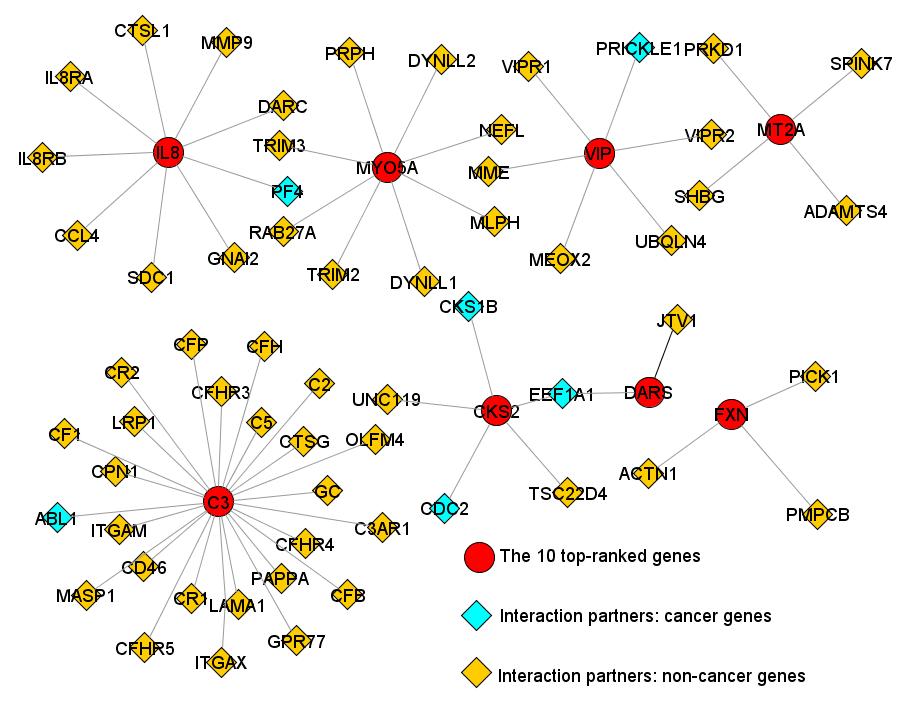


**Fig. S7.** Protein-protein interaction networks associated with the respective ten top-ranked genes ofthe colon tumor dataset.

For the DLBCL dataset, eight genes (RHOH, HLA-A, NCOR2, CDKN3, MCM7, SNRPB, TPI1 and PMSC1) are identified to link with cancer genes with CLD of 1, 7, 31, 4, 13, 2, 1 and 1, respectively.For the remaining two genes of the top 10, CD180 probably cooperates with MD-1 and TLR4 to mediate the innate immune response to bacteriallipopolysaccharide (LPS) in B-cell andleads to NF-kappa-B activation and thelife/death decision of B-cells.Polson et al.[144]identified CD180 as one target of the seven antigens (CD19, CD20, CD21, CD22, CD72, CD79b, and CD180) for potential treatment of non–Hodgkin's lymphoma (NHL) withAntibody-drug conjugates.DLBCL is categorized as one of the aggressive non-Hodgkin's lymphomas (NHLs). The serum Lactate dehydrogenase (LDHA) is incoporated into the International Prognostic Index widely usedfor prediction of outcome in patients with aggressive NHL[145].Mutations in LDHA have been linked to exertionalmyoglobinuria.SNRPB, NCOR2 and MCM7 are linked together via DDX20 and NFKBIA, which may be useful to explore the possible DLBCL cancer related subnetwork and even pathways.


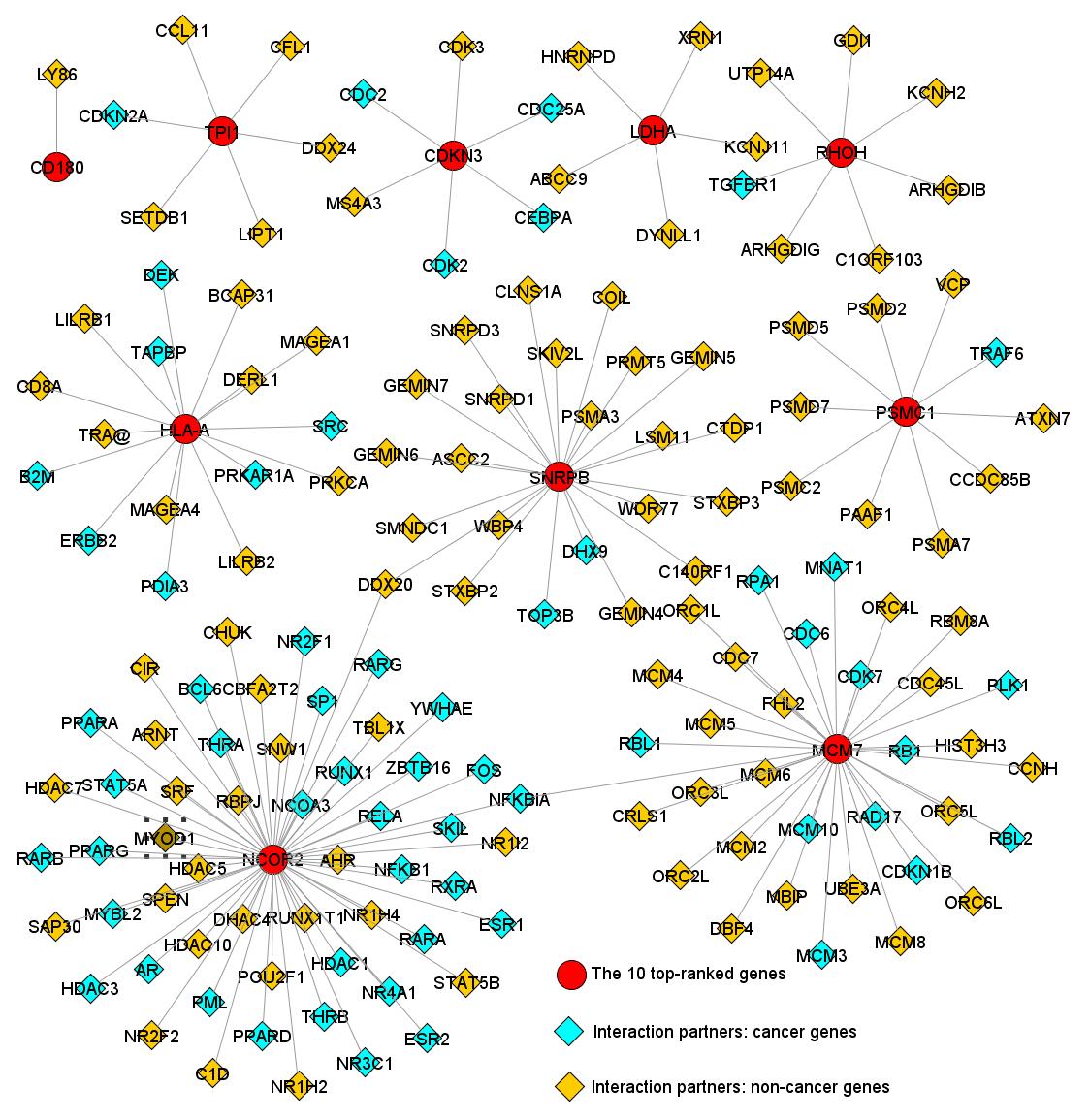


**Fig. S8.** Protein-protein interaction networks associated with the respective ten top-ranked genes ofthe DLBCL dataset.
